# Pollen calendars and maps of allergenic pollen in North America

**DOI:** 10.1007/s10453-019-09601-2

**Published:** 2019-07-17

**Authors:** Fiona Lo, Cecilia M. Bitz, David S. Battisti, Jeremy J. Hess

**Affiliations:** 1 Department of Atmospheric Sciences, College of the Environment, University of Washington, Seattle, WA USA; 2 Department of Emergency Medicine, School of Medicine, University of Washington, 4225 Roosevelt Way NE #100, Suite 2330, Box 354695, Seattle, WA 98105 USA; 3 Department of Environmental and Occupational Health Sciences, School of Public Health, University of Washington, Seattle, WA USA; 4 Department of Global Health, Schools of Medicine and Public Health, University of Washington, Seattle, WA USA

**Keywords:** Allergy, Aeroallergens, *Quercus*, Start date, Duration, Latitude

## Abstract

Pollen is a common allergen that causes significant health and financial impacts on up to a third of the population of the USA. Knowledge of the main pollen season can improve diagnosis and treatment of allergic diseases. Our objective in this study is to provide clear, quantitative visualizations of pollen data and make information accessible to many disciplines, in particular to allergy sufferers and those in the health field. We use data from 31 National Allergy Bureau (NAB) pollen stations in the continental USA and Canada from 2003 to 2017 to produce pollen calendars. We present pollen season metrics relevant to health and describe main pollen season start and end dates, durations, and annual pollen integrals for specific pollen taxa. In most locations, a small number of taxa constitute the bulk of the total pollen concentration. Start dates for tree and grass pollen season depend strongly on latitude, with earlier start dates at lower latitudes. Season duration is correlated with the start dates, such that locations with earlier start dates have a longer season. NAB pollen data have limited spatiotemporal coverage. Increased spatiotemporal monitoring will improve analysis and understanding of factors that govern airborne pollen concentrations.

**Electronic supplementary material:**

The online version of this article (10.1007/s10453-019-09601-2) contains supplementary material, which is available to authorized users.

## Introduction

Pollen allergies are widespread and associated with several chronic conditions, including allergic rhinitis, allergic conjunctivitis, and allergic asthma, with allergic rhinitis the most common (Pawankar et al. 2011). The Centers for Disease Control and Prevention’s 2016 National Health Interview Survey (Centers for Disease Control and Prevention 2016) estimated allergic rhinitis prevalence in the USA at 21.5 million (6.5% of adults and 7.5% of children), though estimates using self-reported symptoms approach 30% for the total US population (Wheatley and Togias 2015). Allergic rhinitis is a risk factor for asthma, and the two diseases are highly correlated, though allergic asthma is less prevalent (Bousquet et al. 2008). Altogether, allergic diseases impose a significant financial burden in the USA, with direct cost of treatment and medications estimated at $11.2 billion in 2005 (Meltzer and Bukstein 2011), and substantial indirect costs from lower workplace productivity, adverse school performance, and reduced quality of life (Lamb et al. 2006; Marcotte 2015; Nathan 2007). This burden is a significant public health concern.

Pollen allergy is a regionally variable disease driven by numerous environmental factors, including local flora, weather, climate, and air pollution (i.e., Sung et al. 2017; Lou 2017; Silverberg et al. 2015; De Weger et al. 2013; Ziska et al. 2003). Prior pollen exposure drives disease sensitization, while current pollen exposure drives exacerbation of disease among those who are sensitized (Kihlström et al. 2002; Jantunen et al. 2012). The temporal and spatial distributions of allergenic pollen types are important to allergic disease epidemiology and in diagnosis and management of allergic diseases. Pollen calendars are useful for visualizing and understanding the distribution, timing, and concentration of different pollen taxa at given locations and can help allergy sufferers and clinicians identify potential triggers, guide diagnostic testing, and initiate appropriate therapies (Katotomichelakis et al. 2015). Pollen calendars can also help public health officials assess exposure, develop early warning systems, improve guidance to limit exposure, and promote therapy in advance of high pollen loads. Although some pollen grains can be transported hundreds to thousands of kilometers in the atmosphere (Rogers and Levetin 1998; Campbell et al. 1999; Sofiev et al. 2006), local pollen emissions are the principal driver of pollen concentrations in a given area (Keynan et al. 1991; Ranta et al. 2006). Pollen calendars are thus location specific, with pollen concentrations closely linked to the local distribution of flora, meteorology, and climate.

To understand pollen concentrations on a continental scale, large-scale coordinated studies are necessary. Summarizing pollen calendar research in Europe, D’Amato et al. (1998) concluded that a continent-wide understanding of pollen concentrations was not possible due to inconsistent methods across studies and regionally fragmented sampling. There have been some single-station pollen calendar studies in the continental USA and Canada (Kosisky et al. 2010; Levetin 1998; Fuhrmann et al. 2016; Rogers 1997). A few studies examine the large-scale distribution of pollen in North America (Solomon and Platts-Mills 1998; Rogers 2001); however, recent studies have focused on changes over time rather than on regional pollen distributions (Zhang et al. 2014a). Our work updates main pollen season characteristics by describing the seasonal dynamics, timing, and regional variations of major allergenic pollen concentrations across the continental USA and Canada.

## Methods

### Pollen data

We obtained pollen data from the National Allergy Bureau (NAB), a section of the American Academy of Allergy Asthma and Immunology’s (AAAAI) Aeroallergen Network. The NAB aggregates and manages distribution of pollen data collected at the NAB stations. Pollen stations are run by AAAAI member volunteers and are self-funded.

A station in the NAB network is required to collect pollen samples at a minimum of 3 days per week from an unobstructed rooftop at least one story above ground with no local pollen sources. Pollen counts are collected with a Burkard volumetric air sampler or a Rotorod rotation impaction sampler. The Burkard collects higher counts than the Rotorod, particularly for smaller particles, and is more sensitive to wind speed (Frenz 1999; Crisp et al. 2013). Nonetheless, daily pollen counts using the two methods are positively and significantly correlated, and the absolute difference associated with the sampling instruments is small enough that it may not be meaningful from a clinical standpoint (Crisp et al. 2013). We will use and compare pollen counts sampled from both devices. Daily pollen counts are reported as daily average pollen concentrations (pollen grains/m^3^) which is the number of pollen grains divided by the volume of the air sampled over 24 h.

The NAB provided data from 51 stations for 2003–2017: 50 stations in the continental USA and one station (London, ON) in Canada. For simplicity, we will refer to the region covered by these stations as the Continental USA and Southern Canada (CUSSC). For stations to be included in our study, we required at least 2 years of data and with an average of three or more days per week of data between March 1 and October 1 for all years sampled. We excluded individual years of station data for a given taxon if the annual sum of the daily pollen concentration was 10 pollen grain*day/m^3^ or less, or if sampling began on or after June 1 of that year. Cumulative pollen concentrations are integrals of concentration over time, so are given in units of pollen grain*day/m^3^.

The NAB pollen data are grouped into 43 pollen categories: 38 for specific genera and families and five other composite categories: “Total Pollen,” “Other Tree Pollen,” “Other Weed Pollen,” “Other Grass Pollen,” and “Unidentified Pollen.”

### Pollen calendars

We created pollen calendars by taking the daily average pollen concentrations for eligible years. Average annual pollen integral concentrations of less than 150 pollen grain*day/m^3^ were considered to have insufficient collection of data for a particular taxon, so pollen calendars only include pollen taxa with an average annual integral concentration greater than 150 pollen grain*day/m^3^.

### Pollen season indices

Pollen season indices describe characteristics of the main pollen season. We chose to use pollen indices relevant to health: annual pollen integral (APIn), season start and end dates, and season duration. APIn is correlated with allergy symptom severity among sensitized individuals (Bastl et al. 2016). Knowledge of start dates is important for initiating medical therapy because antihistamine and anti-inflammatory allergy medications can take 1–4 weeks to be fully effective. This information can also be used to modify immunotherapy: patients in immunotherapy are exposed to increasing allergen doses and may be at risk of anaphylaxis if immunotherapy dosing is advanced when ambient pollen concentrations are increasing. Knowledge of end dates is useful for public health surveillance and for deciding when medical therapy can be discontinued.

#### Annual pollen integral (APIn)

The APIn is the integral of the daily pollen concentration for a specific taxon over the pollen year. A pollen year is a year that includes one complete pollen season, beginning when the plant is dormant. In most regions of CUSSC, the pollen year begins with the calendar year on January 1, but in warmer regions some pollen taxa are present in the atmosphere before January 1, in which case the pollen year begins earlier. Most *Ambrosia* species are short-day plants and they flower when the duration of daylight begins to decrease. However, there are some *Ambrosia* species in the Southwestern USA, southern California and coastal Florida that flower in the spring. We do not have pollen data from these areas and no data on spring-flowering *Ambrosia*, and so we define the pollen year for *Ambrosia*, using the more common fall-flowering species, to begin on the summer solstice, June 21. For other taxa, we assessed pollen concentrations to determine their dormant periods. Using these criteria, we define the pollen years to be January 1–December 31, except for stations in California, Texas, Georgia, and Oklahoma, where pollen years are September 1–August 31 for *Cupressaceae*, November 1–October 31 for *Fraxinus*, and December 1–November 30 for all other taxa.

#### Start date of the main pollen season

A variety of approaches to defining start and end dates of the main pollen season have been taken (Jato et al. 2006). A common approach is to define a start date as the date when the integral of the pollen concentration over the pollen year exceeds threshold percentage of the APIn for a given year. However, this approach has several disadvantages. First, it is necessarily retrospective, so the start date cannot be computed until the pollen year is over and the APIn is known. Second, because the threshold value is a percentage of the APIn, it varies year to year with fluctuating APIns. Third, it is location specific and makes interpretation of start date over a large region difficult. We chose our metric to avoid these pitfalls and to allow for a priori calculation based on historical APIns.

Studies have found that mild allergy symptoms are observed at relatively low pollen concentrations of ~ 10–20 pollen grains/m^3^, moderate symptoms at ~ 50–90 pollen grains/m^3^, and severe symptoms at ~ 80–90 pollen grains/m^3^ (Rapiejko et al. 2007; Negrini et al. 1992; Frenz 2001; de Weger et al. 2013). For most taxa, we define the start date as the day when the integral of pollen concentration over that pollen year reaches a threshold of 50 pollen grain*day/m^3^. Sensitive allergic individuals likely experience symptoms below this threshold. Due to the priming effects of allergens, a phenomenon in which increased allergic response is observed with daily sequential exposure (Sin and Togias 2011; Bruin-Weller et al. 1999; Connell 1968), allergic symptoms may occur at a cumulative threshold of 50 pollen grain*day/m^3^. For taxa with APIn below 2000 pollen grain*day/m^3^, we define the start date as the date on which the integral reaches a threshold of 2.5% of the historical mean APIn. The start date of the main pollen season is computed for each pollen taxon at each station location for every year.

NAB pollen taxon categories are either families or genera, and they can be composed of many species. As a result, there may be a diverse range of timing for pollen release for different species within a taxon. Calculations of the start date of the main pollen season for a specific taxon will be the start date of the species that releases pollen first and may not be indicative of the start date for other species within that taxon.

To evaluate the interannual variability, the standard deviation of start date was calculated for each important allergenic pollen. This was done by (1) obtaining the anomalous start dates for each station by subtracting the long-term mean start date for that station, and then (2) stringing together the anomalous start dates from all stations for which there were reliable start dates for each taxon at each station (which ensures a stable long-term mean value at each station).

#### End date of the main pollen season

The end date is calculated in a similar manner to the start date. For taxa with high APIn (> 2000 pollen grain*day/m^3^), the end date is defined as the date at which the integral of pollen concentration from that date to the end of the pollen year is less than 50 pollen grain*day/m^3^. If the long-term mean APIn is less 2000 pollen grain*day/m^3^, then the end date threshold is calculated as the date at which accumulated pollen concentration reaches 97.5% of the long-term mean APIn.

#### Duration of the main pollen season

Duration of the main pollen season is the number of days between the start date and the end date of main pollen season inclusive.

## Results

### Pollen data used in analysis

#### Pollen sampling characteristics

**Fig. 1:**
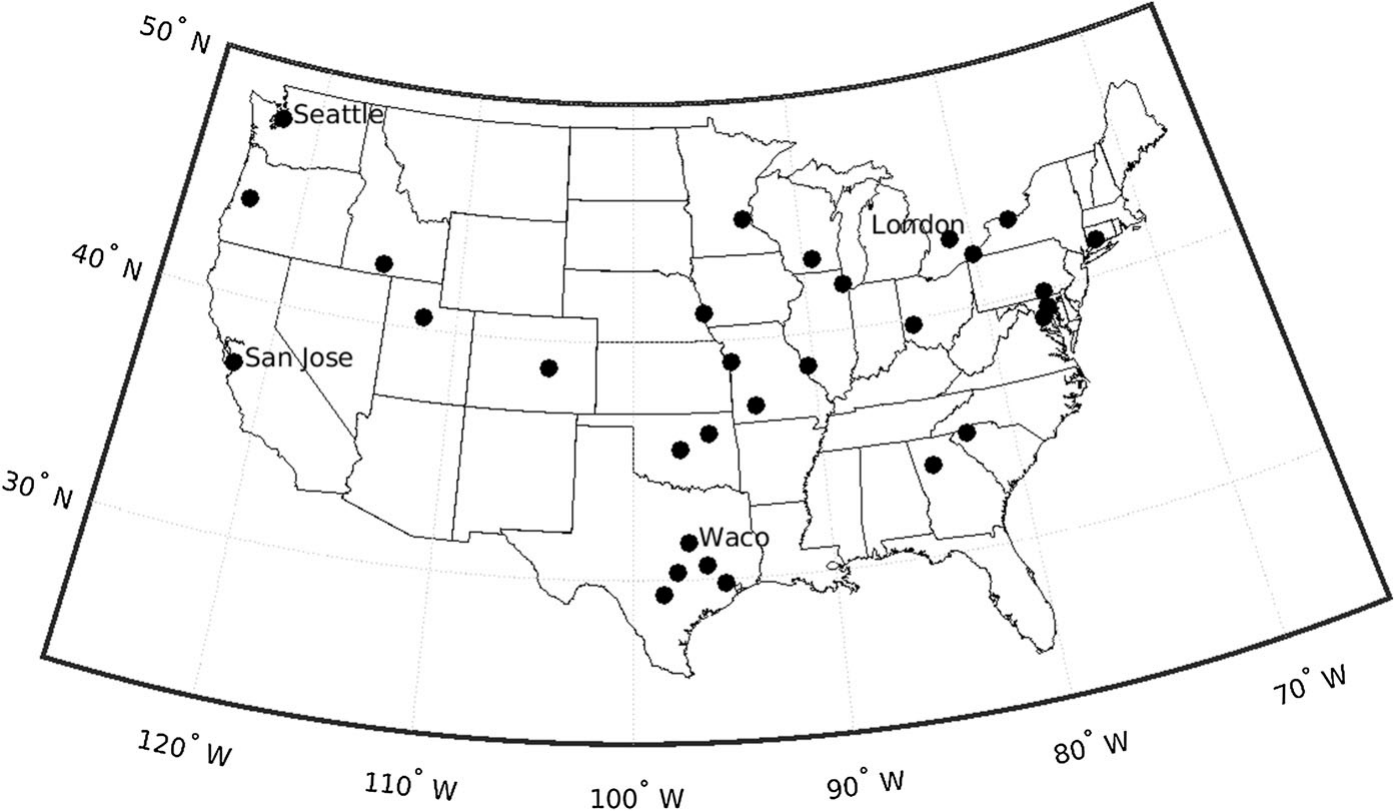
National Allergy Bureau (NAB) station locations that meet inclusion criteria

Of the 51 stations received from the NAB, 31 stations met inclusion criteria (Fig. 1, Table 1).

**Table 1:** National Allergy Bureau (NAB) station locations, air sampler used to collect pollen, average sampling range over the calendar year, and average percentage of days sampled

Location of station	Latitude (°N)	Longitude (°W)	Air Sampler	Years	Average first sampling date	Average last sampling date	Percentage of days sampled between average first and last sampling date	Percentage of days sampled over calendar year
Atlanta, GA*	33.8	84.4	Burkard	2003–2017	Jan 03	Dec 29	69.8	68.9
Austin, TX*	30.3	97.8	Burkard	2003–2017	Jan 06	Dec 29	71.0	69.5
Baltimore, MD*	39.3	76.6	Rotorod	2003–2017	Mar 02	Oct 24	90.0	58.1
Bellevue, NE*	41.1	95.9	Burkard	2003–2017	Feb 15	Dec 03	91.0	72.5
Charlotte, NC	35.2	80.8	Rotorod	2012–2017	Feb 15	Nov 10	30.4	22.3
Coeur d’Alene, ID	47.7	116.8	Burkard	2011–2017	Mar 29	Sep 02	26.6	11.4
College Station, TX*	30.6	96.3	Burkard	2003–2017	Jan 01	Dec 28	66.0	65.2
Colorado Springs, CO*	38.8	104.7	Rotorod	2006–2017	Feb 15	Nov 06	92.3	66.7
Dayton, OH*	39.7	84.2	Burkard	2003–2017	Jan 08	Dec 23	66.2	63.4
Draper, UT*	40.5	111.9	Burkard	2003–2017	Mar 08	Oct 16	61.2	37.1
Erie, PA*	42.1	80.1	Burkard	2003–2017	Apr 08	Oct 16	62.8	32.8
Eugene, OR*	44.0	123.1	Burkard	2003–2016	Jan 08	Dec 25	51.5	49.4
Findlay, OH	41.0	83.7	Burkard	2014–2016	May 17	Jul 24	30.4	5.6
Greenville, SC*	34.9	82.4	Burkard	2003–2017	Feb 04	Dec 09	61.1	51.6
Houston, TX*	29.8	95.4	Burkard	2011–2017	Jan 03	Dec 29	67.6	66.7
Kansas, City MO*	39.1	94.5	Burkard	2003–2017	Feb 28	Nov 04	67.5	46.1
Knoxville, TN	36.0	84	Burkard	2003–2016	Mar 10	Oct 07	29.4	17.0
La Crosse, WI	43.9	91.2	Rotorod	2003–2016	Mar 25	Sep 28	48.3	24.7
London, ON*	43.0	81.2	Burkard	2003–2017	Feb 09	Oct 28	95.1	68.1
Louisville, KY	38.2	85.7	Burkard	2003–2016	Jan 06	Dec 29	90.6	88.7
Madison, WI*	43.1	89.4	Rotorod	2003–2017	Mar 21	Oct 22	54.9	32.3
Melrose Park, IL*	41.9	87.8	Burkard	2003–2017	Mar 25	Oct 16	69.1	38.6
Midland, TX	31.9	102.1	Burkard	2014–2017	Jan 26	Dec 13	24.1	21.2
Minneapolis, MN*	45.0	93.4	Rotorod	2010–2017	Mar 19	Nov 02	76.1	47.4
Mount Laurel, NJ	40.0	74.9	Burkard	2003–2016	Mar 09	Oct 30	44.7	28.7
New Castle, DE	39.6	75.6	Burkard	2005–2017	Mar 10	Oct 25	36.3	22.8
Oklahoma City1, OK*	35.5	97.5	Burkard	2003–2017	Jan 04	Dec 21	67.1	64.5
Oklahoma City2, OK*	35.5	97.5	Burkard	2003–2017	Jan 12	Dec 21	54.2	50.9
Oklahoma City3, OK	35.5	97.5	Burkard	2015–2017	Apr 28	Dec 22	38.7	25.1
Olean, NY	42.1	78.4	Burkard	2003–2017	Apr 01	Nov 05	39.8	23.7
Philadelphia, PA	40.0	75.1	Burkard	2003–2017	Mar 14	Oct 21	46.7	28.2
Pleasanton, CA	37.7	121.9	Burkard	2003–2017	Jan 12	Dec 18	26.1	24.3
Pueblo, CO	38.2	104.6	Rotorod	2012–2016	Mar 13	Oct 19	42.2	25.3
Rochester, NY*	43.2	77.6	Burkard	2003–2017	Mar 11	Oct 23	70.6	43.6
Roseville, CA	38.8	121.2	Burkard	2007–2017	Jan 08	Dec 25	13.8	13.3
Saint Louis, MO*	38.6	90.3	Burkard	2003–2016	Jan 07	Dec 29	68.9	67.3
San Antonio2, TX*	29.4	98.5	Burkard	2010–2017	Jan 21	Dec 27	93.1	86.7
San Antonio3, TX	29.4	98.5	Burkard	2014–2017	Jan 01	Dec 30	99.6	99.1
San Jose, CA*	37.2	121.7	Burkard	2003–2017	Jan 09	Dec 22	82.6	78.5
Seattle, WA*	47.6	122.3	Burkard	2003–2017	Jan 26	Aug 25	85.6	49.6
Sparks, NV	40.1	119.6	Rotorod	2003–2017	Feb 16	Oct 25	19.7	13.5
Springfield, MO*	37.2	93.3	Burkard	2009–2017	Mar 28	Oct 30	69.2	40.9
Sylvania, OH	41.7	83.7	Burkard	2014–2017	Mar 11	Nov 08	91.5	60.6
Tampa, FL	27.9	82.5	Burkard	2003–2017	Jan 18	Nov 12	32.7	26.7
Tulsa, OK*	36.1	96.0	Burkard	2003–2017	Jan 09	Dec 19	45.9	43.3
Twin Falls, ID*	42.4	114.6	Rotorod	2003–2017	Mar 13	Oct 17	47.9	28.6
Waco, TX*	31.6	97.2	Burkard	2003–2017	Jan 06	Dec 29	65.7	64.3
Washington, DC*	38.9	77.0	Burkard	2003–2016	Jan 07	Dec 27	56.8	55.1
Waterbury, CT*	41.4	73.0	Burkard	2003–2017	Mar 31	Sep 29	69.2	34.2
Waukesha, WI	43.0	88.3	Burkard	2003–2016	Mar 31	Oct 16	21.6	11.8
York, PA*	40.0	76.7	Rotorod	2003–2017	Mar 15	Oct 19	66.0	39.3

Asterisk (*) indicates stations that meet inclusion criteria

#### Important allergenic pollen taxa

We elected to focus on eleven important allergenic pollen taxa in the CUSSC region as determined by their abundance in CUSSC (Table 2) and guided by previous studies (Lewis et al. 1983; Park et al. 2014; de Weger et al. 2013; Emberlin 2009). In the past, *Pinaceae* pollen has been considered a mild allergen and disregarded as an important allergenic pollen. Recent studies suggest that rates of allergic reactivity to *Pinaceae* pollen are on the rise (Park et al. 2014) and that the abundance of *Pinaceae* pollen and cross-reactivity of *Pinaceae* pollen with *Poaceae* pollen warrant *Pinaceae* pollen to be considered potentially allergenic (Gastaminza et al. 2009). We will refer to pollen taxa by their scientific names, and some of their common names are also provided in Table 2.

**Table 2:** Description and allergenic potential of 11 most important pollen taxa in the CUSSC region ranked by percent abundance relative to the sum of all pollen taxa over 31 NAB stations that meet inclusion criteria, 2003–2017

Rank	Scientific name (taxon)	Common name(s)	Pollen group	Percent abundance	Description
1	*Quercus* (genus)	Oak	Tree	19.6	Most *Quercus* trees produce heavy pollen loads. *Quercus* genus has many species found all over the CUSSC. They are commonly found in residential areas, parks, and forests
2	*Cupressaceae* (family)	Cypress, Juniper, Cedar	Tree	19.4	Most allergenic species in this family are evergreen conifers, including the genera *Cupressus* (Cypress) and *Juniperus* (Juniper). All *Cupressaceae* shed profuse amounts of pollen. Reactions to *Cupressaceae* pollen are often severe
3	*Ambrosia* (genus)	Ragweed	Weed	7.2	*Ambrosia* typically grows in areas that have been disturbed and left bare (e.g., agricultural borders and river banks). *Ambrosia* pollen is the primary cause of late summer, early fall allergic symptoms. *Ambrosia* is found throughout the CUSSC; it is most common in the Midwest
4	*Morus* (genus)	Mulberry	Tree	6.7	Allergenic reaction to *Morus* pollen is often severe. *Morus* is found throughout the CUSSC
5	*Pinaceae* (family)	Pine	Tree	4.5	*Pinaceae* family consists of evergreen coniferous trees and shrubs. They release large amounts of pollen. The pollen is considered to be low allergenic potential because the grains are large and contain fewer number of allergens, but *Pinaceae* pollen are potentially allergenic where abundant
6	*Ulmus* (genus)	Elm	tree	4.6	All *Ulmus* produce allergenic pollen and release significant amounts of pollen. Deciduous *Ulmus* produce pollen in early spring. *U. parvifolia* (Chinese elms) are evergreen in areas with warm winters and their pollen is very allergenic
7	*Fraxinus* (genus)	Ash	Tree	3.7	Deciduous *Fraxinus* trees can produce copious amounts of potent pollen. *Fraxinus* trees are widespread in the CUSSC.
8	*Betula* (genus)	Birch	Tree	3.8	*Betula* are usually smaller trees, so do not produce a large volume of pollen but *Betula* pollen is a well-known aeroallergen. They have a short pollen season as *Betula* blooms for only a few days
9	*Poaceae*, *Gramineae* (family)	Grass	Grass	3.7	Grass lawns are found throughout the country and used for urban landscaping in gardens and parks. Popular lawn grasses include *Poa* pratensis (Bluegrass), *Phleum pretense* (Timothy Grass), *Cynodon dactylon* (Burmuda) and *Lolium* (Ryegrass). *Poaceae* is also grown for pasture and hay. *Poaceae* pollen is highly allergenic
10	*Acer* (genus)	Maple	Tree	3.7	*Acer* are deciduous trees with a large number of species. Many species cause allergies, but not all. *Acer* are commonly found in the eastern CUSSC region and are also popular for ornamental and street plantings
11	*Populus* (genus)	Poplar, aspen, cottonwood	Tree	2.5	*Populus* trees such as poplars, aspens, and cottonwoods are large, deciduous trees. They are found throughout the CUSSC

#### Reliability of main pollen season start date calculation

**Fig. 2:**
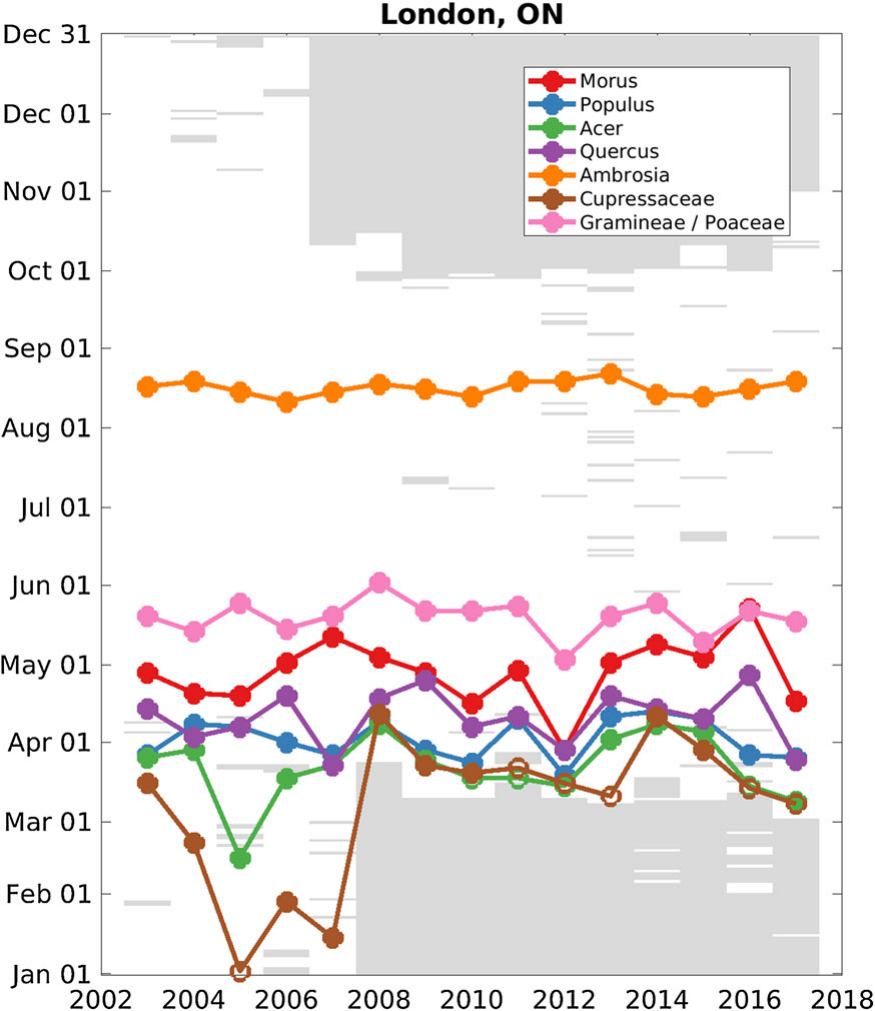
Start date of the main pollen season at London, ON, for the taxa: *Cupressaceae* (brown), *Acer* (green), *Populus* (blue), *Quercus* (purple), *Morus* (red), *Poaceae* (pink), and *Ambrosia* (orange). Open circles indicate unreliable start dates (calculated start dates that occur within 7 sampled days of the first sampling date). Filled circles indicate reliable start dates. Grayed out areas are dates at which pollen was not sampled

Our choice of start date metrics balances sensitivity to relatively low pollen concentrations, an important consideration from a health standpoint, with robustness to missing values, a significant consideration with this pollen dataset. As explained in “Methods,” our start date is defined based on an integral of pollen concentrations that begin on the first day of the pollen year (usually January 1) and days with missing data do not contribute. Unfortunately, it is not uncommon to have measurements begin in a calendar year after pollen is already present in the atmosphere. In these cases, the calculated start date is biased late. We illustrate this sampling problem with time series of start date for seven of the most abundant pollen taxa from London, ON (Fig. 2). The station began sampling pollen around January 1 in 2003–2007 and in mid-March in 2008–2017. The start dates for *Cupressaceae* pollen in the period of 2008–2017 almost immediately follow the date of the first sampling in those calendar years and are much later than in the period 2003–2007, suggesting that data collection between 2008 and 2017 sometimes began after the *Cupressaceae* main pollen season had already started.

*Cupressaceae* can be one of the earliest tree pollen taxa to emit pollen, with observations as early as August in Waco, TX (see Sect. 3.3.1). A start date is considered unreliable if the calculated start date occurs within 7 sampled days of the first sampling date. Only 28% of station-years have reliable start dates for *Cupressaceae* pollen (not shown). Unreliable start dates are not further included in our study. Start dates can be determined with confidence for taxa that emit pollen later in the season, such as *Quercus*.

### Proportional distribution of allergenic pollen taxa

**Fig. 3:**
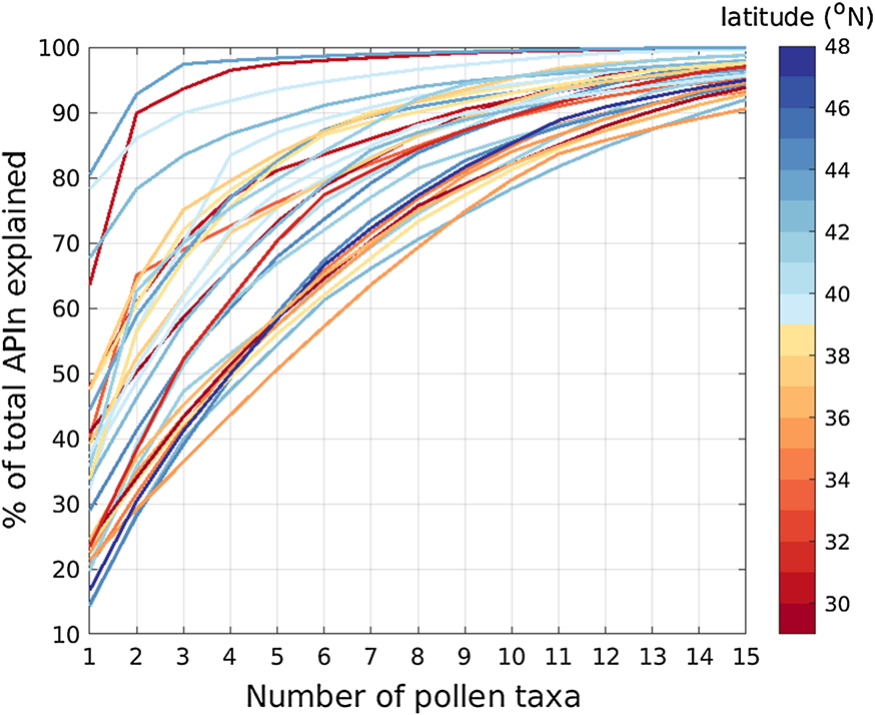
Cumulative percentage of APIn by number of pollen taxa. Each line represents a station; the color of the line indicates latitude

The number of dominant pollen taxa varies among stations. For all stations in the CUSSC region, 70% of the APIn at a location is comprised of eight or fewer pollen taxa (Fig. 3). Relative abundances of the important allergenic taxa show that *Quercus* and *Cupressaceae* are the most abundant pollen taxa in the CUSSC (Table 2, stacked bar chart and radar charts in supplementary materials).

### Pollen calendars

The airborne pollen season varies in time and space depending on the pollen taxon. We selected four methods for describing the main pollen season to present both the location perspective and the taxon perspective. We first present two types of pollen calendars. The first calendar type describes the long-term mean of all observed allergenic pollen taxa, using four locations as examples. These locations are chosen to highlight regional variability and chosen on the basis of data completeness. Pollen calendars for other locations are available in supplementary materials. The second type of pollen calendar describes taxon-specific seasonal characteristics over the CUSSC region. Again, we present a subset of available results, with complete results available in supplementary materials. Third, we show maps of the long-term average start dates and season duration for specific pollen types to demonstrate the spatial variability of the main pollen season. Lastly, we present taxon-specific start dates for a station to show the year-to-year variability.

#### Location-specific pollen calendars

We describe pollen calendars for four stations in different climate and ecological regions in the CUSSC: Seattle, Washington; San Jose, California; Waco, Texas; and London, Ontario.

**Fig. 4:**
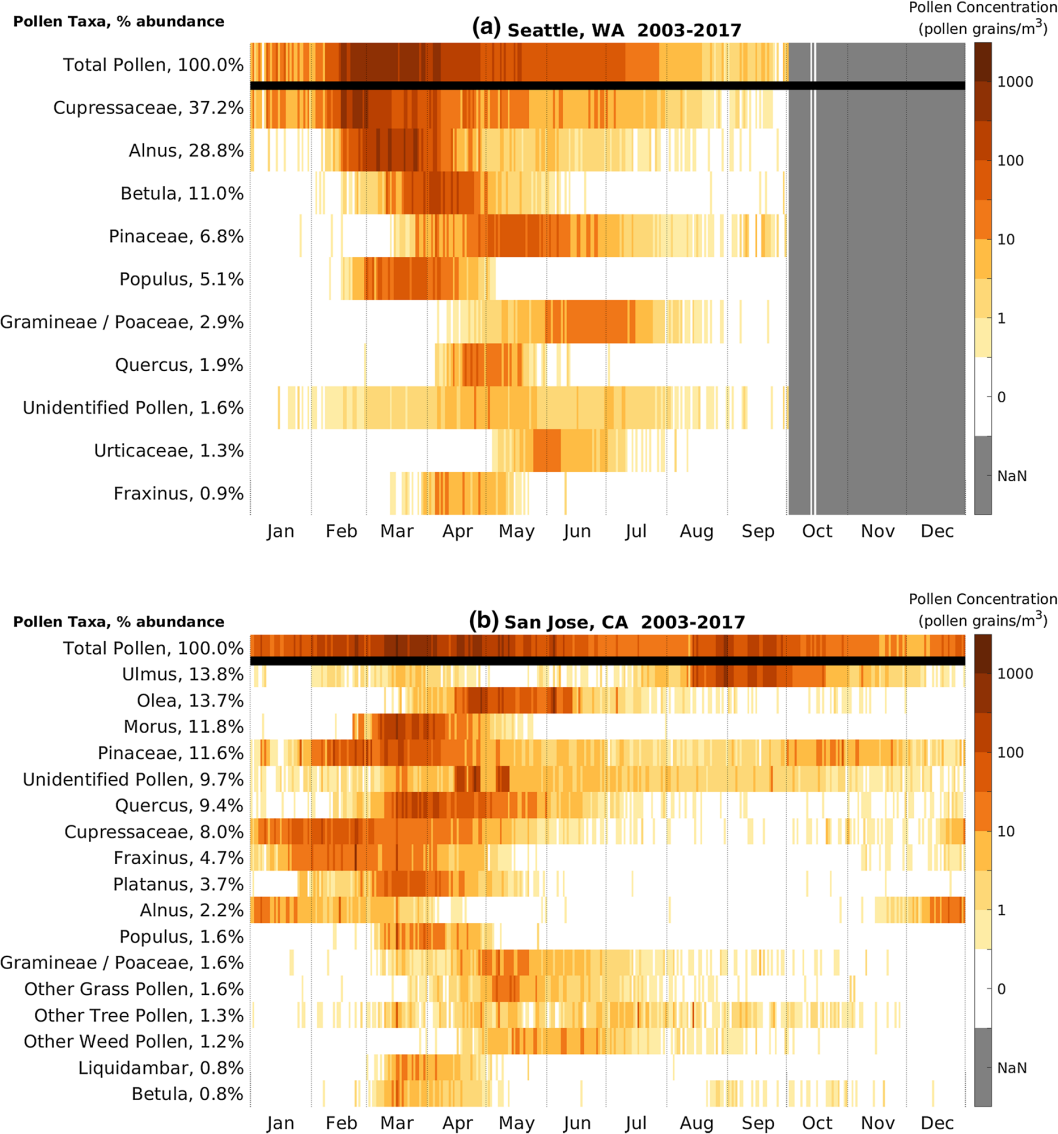

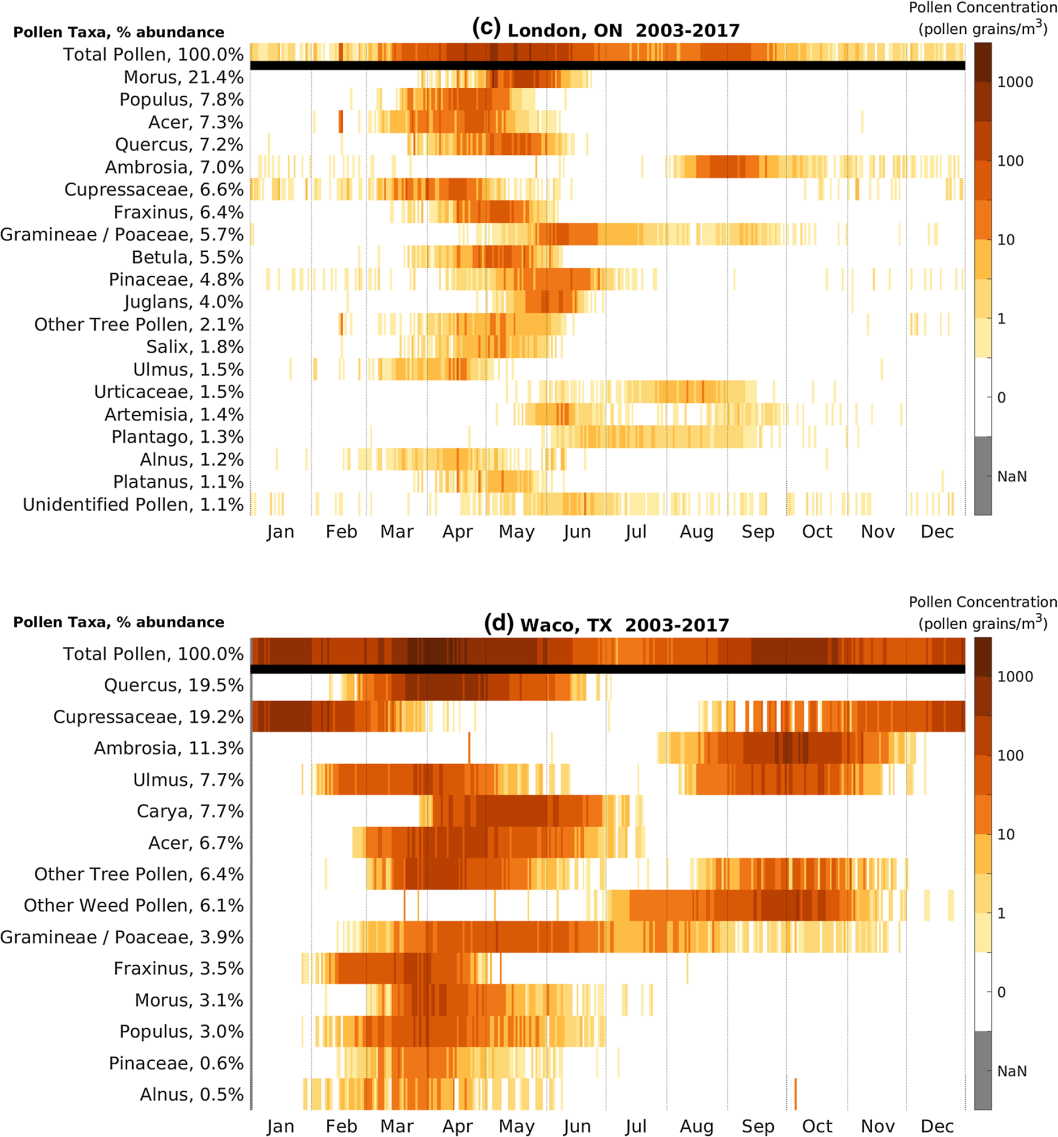
Pollen calendar for **a** Seattle, Washington, **b** San Jose, California, **c** London, Ontario, and **d** Waco, Texas. Daily long-term mean of pollen concentration by pollen taxa, 2003–2017. Percent abundance is the ratio of that taxon’s APIn to the sum of APIn over all pollen taxa. Only pollen taxa with average APIn over 150 pollen grain*day/m^3^ are shown. Missing data are shaded gray and denoted NaN in the color bar

Seattle, Washington, is located in the Pacific Northwest with a cool, moist climate with dry summers and wet, generally snowless winters. Evergreen trees dominate the region. We see evidence of the large number of evergreens in the airborne pollen composition with *Cupressaceae* as the most abundant pollen at 37% (Fig. 4a). Trees dominate the sample, with grass constituting 2.9% and weeds 1.3%. The two most abundant taxa, *Cupressaceae* and Alnus, comprise about two-thirds of the total APIn. *Cupressaceae* is the earliest pollen present; hence, the start of the Seattle main pollen season is primarily governed by *Cupressaceae* pollen. Unlike most other stations, Seattle detects very little pollen after July and thus has a relatively short pollen season.

San Jose, California, is located in central California with a warm, mild Mediterranean climate. The semiarid region receives most of its rainfall in the wintertime. San Jose is an urbanized area within a region of chaparral shrubland mixed with grassland and oak woodlands. San Jose is one of the few NAB stations that sample year-round, which is necessary because pollen is present in the atmosphere throughout the year (Fig. 4b).

Tree pollen is dominant, contributing 94% of the total sample of identified pollen. No one tree pollen dominates; *Ulmus* and *Olea* are most abundant, at 14% each. The double peak in *Ulmus* pollen concentration occurs because there are species that release pollen in the spring as well as in the late summer and early fall. *Olea* pollen is not observed by other NAB stations except in very small amounts; however, it is significant for this area because *Olea* pollen is a potent allergen (Elvira-Rendueles et al. 2017). *Morus* and *Pinaceae* pollen taxa are the next most abundant pollen taxa at almost 12%. *Pinaceae* pollen is a mild allergen; however, it may be considered an important pollen in San Jose because of its abundance and its presence throughout the year (Gastaminza et al. 2009).

London, Ontario, is located in the Great Lakes region with cold winters and humid summers. The land cover is characterized by a mix of agriculture, forest, wetlands, and glacial lakes. Of the total pollen sampled at the station (Fig. 4c), the pollen composition is 81% trees, 13% weeds, and 6% grasses. London tree pollen is diverse, with eight pollen taxa explaining 70% of the APIn. The most abundant is *Morus*, at 22%. London’s main pollen season runs from March through September. The end of the tree pollen season overlaps with the beginning of the grass pollen season in late May. *Ambrosia* is the most abundant weed pollen and has a distinct season from mid-August to mid-September.

Waco, Texas, is located in the south central part of the Great Plains and has a humid subtropical climate with hot dry summers and rains in mid to late spring. Much of the region was originally grassland but is now crops and rangeland. Waco is similar to other stations located in warmer climates in that it has pollen present in the atmosphere throughout the year. Waco’s APIn for all pollen taxa is very high (Fig. 4d) compared to many stations in the CUSSC region but is similar to other nearby stations (Austin and San Antonio, TX).

Waco’s lowest pollen concentrations occur in July, in contrast to more northern stations where the lowest pollen concentrations occur in winter. There are three peaks in the main pollen season in Waco. The earliest peak occurs in January when *Cupressaceae*, which is the second most abundant pollen at 19%, emits pollen. The main peak occurs between late March and late April, when *Quercus*, the most abundant pollen at 20%, peaks along with Carya, *Acer* and spring-pollinating *Ulmus*. The third peak occurs from mid-September through October, when *Ambrosia*, the third most abundant pollen at 11%, fall-pollinating *Ulmus*, and other weed pollen types release pollen.

#### Latitudinal dependence of the main pollen season

**Fig. 5:**
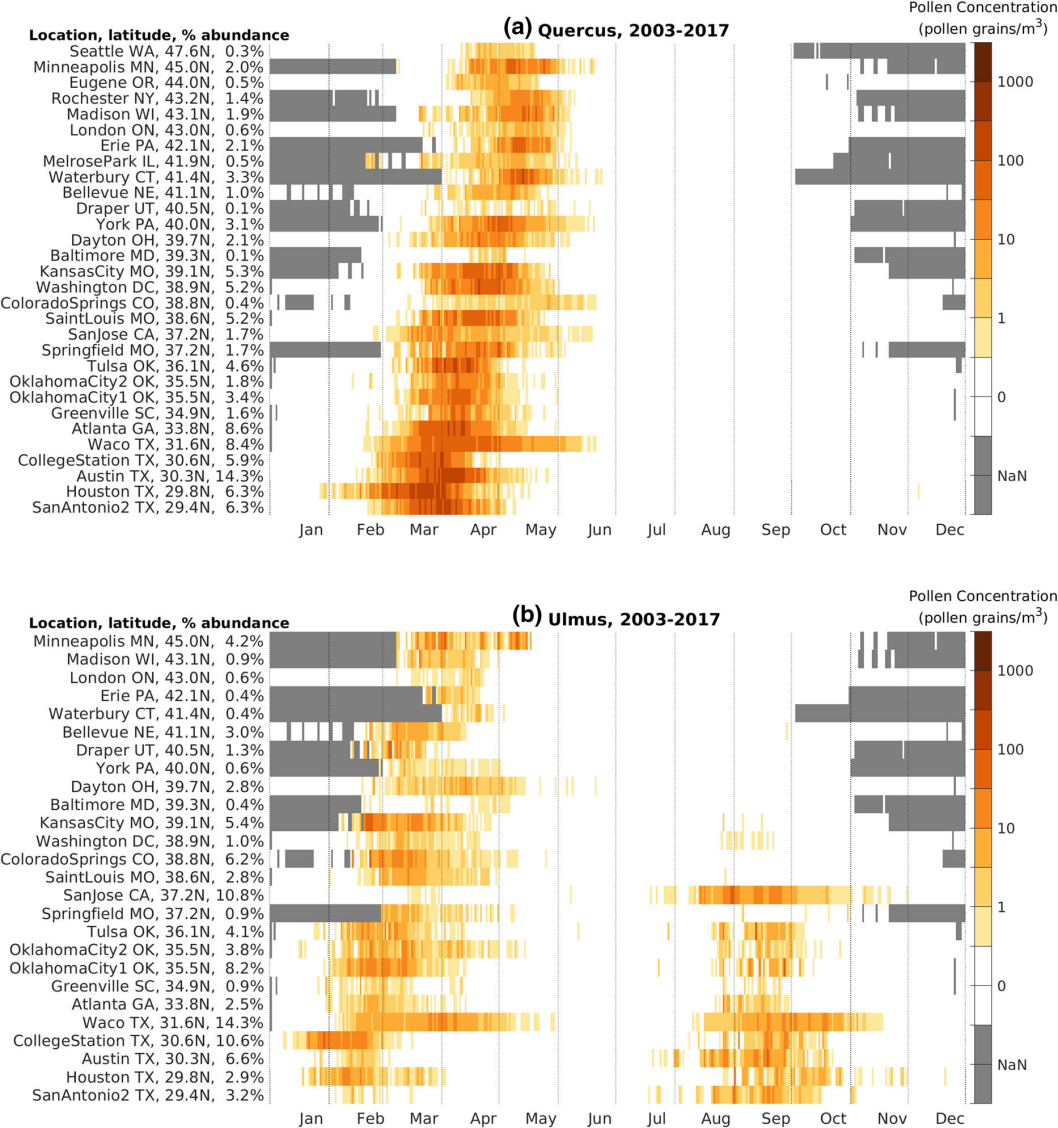

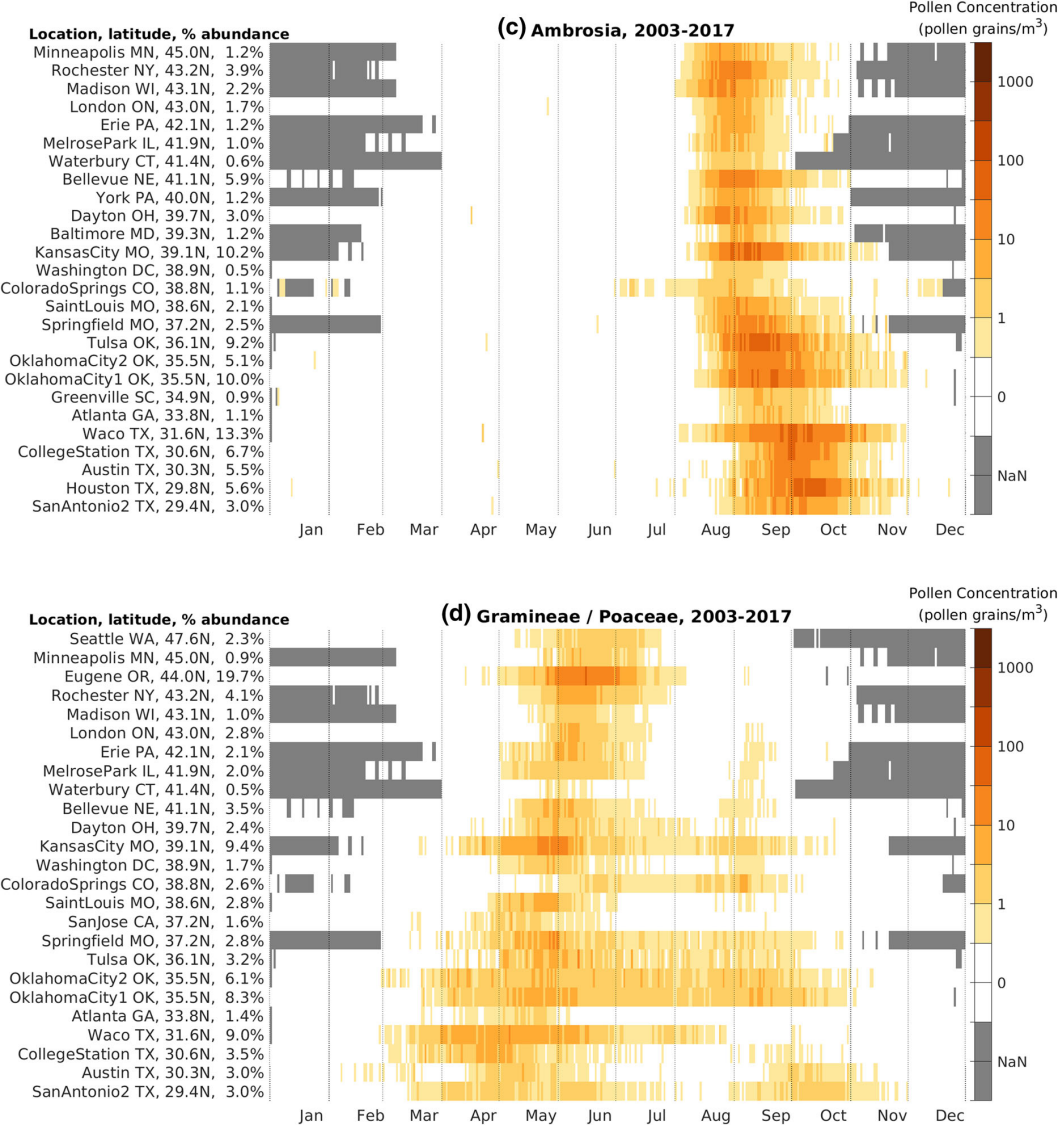
Pollen calendar for **a***Quercus*, **b***Ulmus*, **c***Ambrosia*, and **d***Poaceae*, 2003–2017. NAB stations are ordered by latitude. For each taxon, the percentage abundance is the ratio of the long-term mean APIn at that station to the APIn summed over all NAB stations with data. Missing data are shaded gray and denoted NaN in the color bar. Only NAB stations with average APIn over 150 pollen grain*day/m^3^ are shown

To better understand large-scale patterns of specific pollen taxa, we created taxon-specific pollen calendars with NAB stations ordered by latitude. We present pollen calendars for four of the important allergenic pollen taxa (Fig. 5). In general, stations at lower latitudes have an earlier start to the *Quercus* pollen season (Fig. 5a). In addition, the length of the *Quercus* pollen season is longer at lower latitudes. A similar latitudinal dependence on the start of the main pollen season is observed in all of the important allergenic pollen taxa, with the exception of *Ulmus* and *Ambrosia*.

The pollen calendar for *Ulmus* has a unique pattern, peaking twice annually at lower latitudes (Fig. 5b). This is because some *Ulmus* species release pollen in the spring and others in the late summer and early fall. The spring-pollinating species have a latitude dependence similar to other allergenic tree pollen taxa. The fall-pollinating *Ulmus* species are present only at latitudes south of 39°N and do not have a clear latitude dependence.

The *Ambrosia* pollen calendar (Fig. 5c) exhibits patterns distinct from allergenic trees. *Ambrosia* is most commonly a short-day plant that begins flowering when days begin to shorten, and *Ambrosia* produces pollen in late summer and early fall. At high latitudes, *Ambrosia* season start shows little latitudinal dependence, consistent with plant physiology and others’ findings (Sofiev and Bergmann 2013; Deen et al. 1998). However, the end of the *Ambrosia* season, determined by date of the first frost (Ziska et al. 2011), ends later at lower latitudes.

*Poaceae* are generally known to be a summertime allergen. However, the pollen calendar for *Poaceae* (Fig. 5d) shows that the season can range from March to November. The allergenic *Poaceae* family is comprised of many species, and this can be seen by the various pollen patterns at different stations: Oklahoma City, OK, has one long season; Eugene, OR, has one short season; and Austin, TX, has two distinct seasons in a year. In general, longer duration *Poaceae* seasons occur at lower latitudes, where the season starts earlier and ends later.

### Regional variability in the long-term mean start dates, end dates, and season duration

**Fig. 6:**
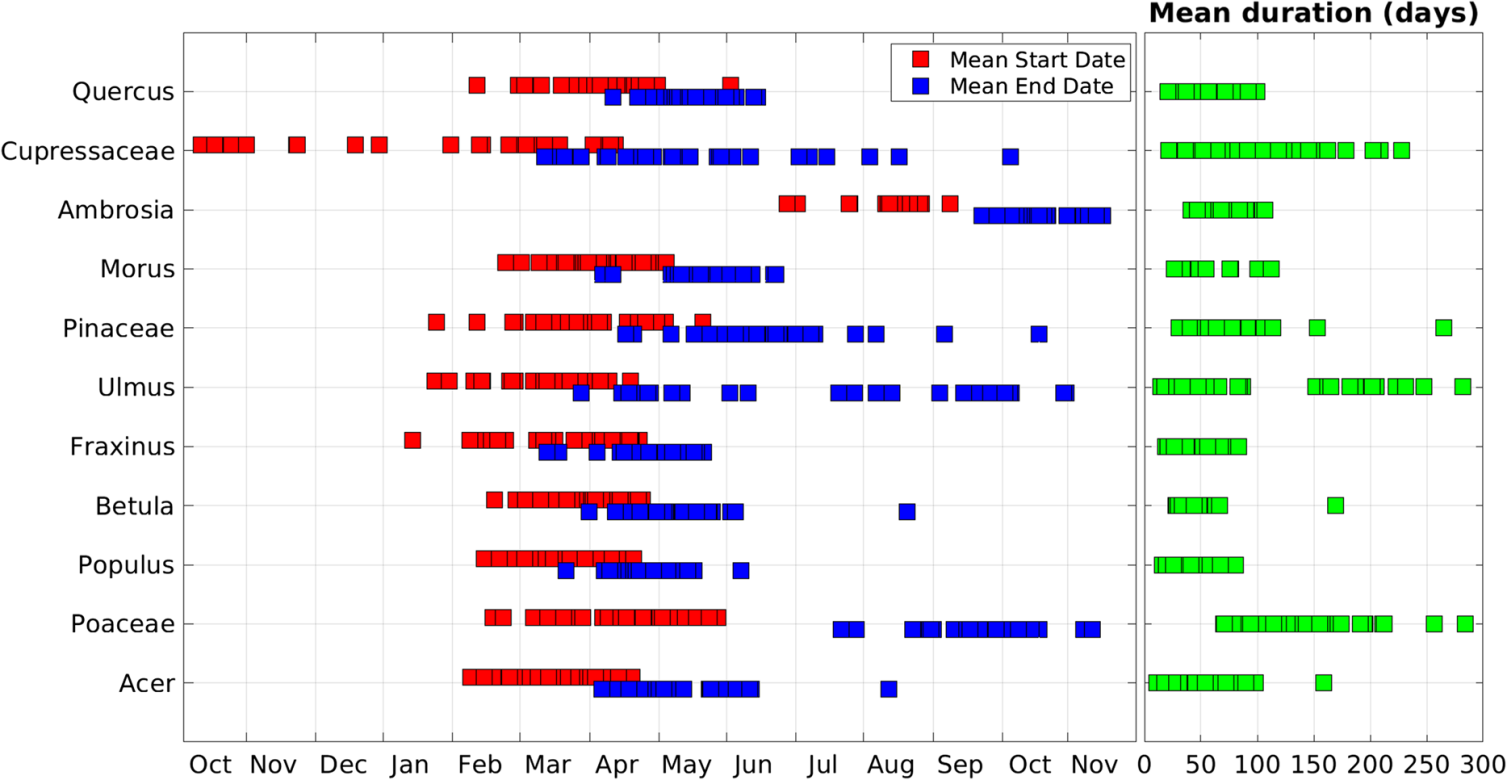
Range of long-term mean main pollen season start dates (red), end dates (blue), and duration (green) for important pollen taxa; each square represents the long-term mean of a NAB station

A summary of the spatial variability of the long-term mean start date, end date, and season duration for each of the 11 important allergenic pollen taxa is shown in Fig. 6. The start dates of *Betula*, *Populus*, and *Acer* have a relatively narrow range among locations, whereas *Cupressaceae*, *Ulmus*, and *Pinaceae* are more variable. Bias start dates due to sampling issues from some stations, and the large number of species in *Cupressaceae* family may contribute to the wide range of start dates for *Cupressaceae* (Sect. 3.1.3). The mean duration of the *Ulmus* main pollen season has two distinct groups corresponding to the presence or absence of fall-pollinating *Ulmus* (Sect. 3.3.2). The mean duration of *Fraxinus* and *Populus* main pollen seasons is relatively similar across the CUSSC, and the mean duration of the *Poaceae* season varies greatly.

#### Start date of the main pollen season

**Fig. 7:**
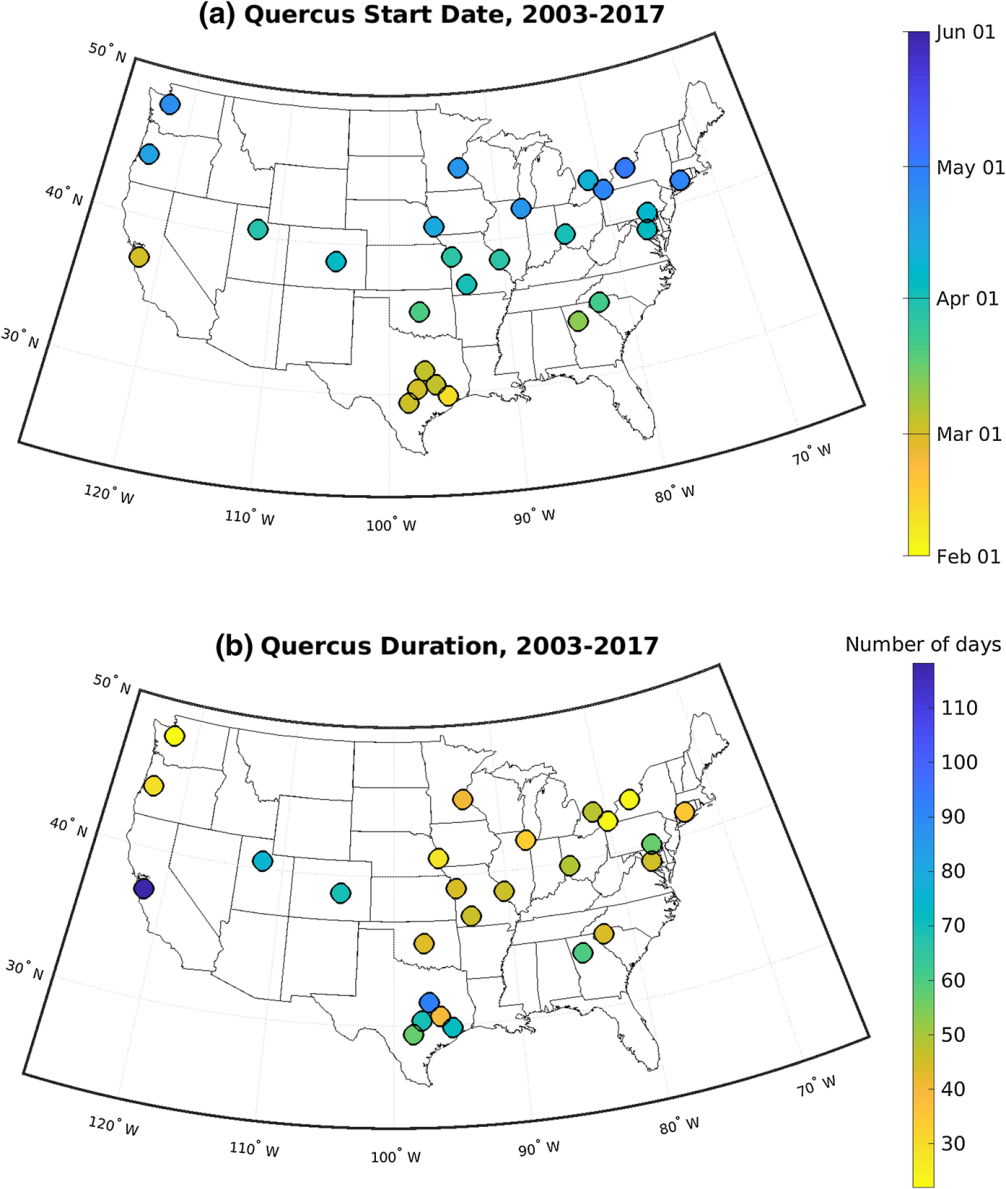
Map of **a** long-term mean start date and **b** long-term mean duration of the *Quercus* main pollen season

There are regional patterns in the mean start date of the main pollen season in the CUSSC. The dominant pattern is a latitudinal dependence in which higher-latitude stations have a later start date (e.g., Figs. 5a, 7a for *Quercus* pollen). *Quercus* pollen is representative of other important allergenic tree pollen taxa in that they all show latitudinal dependence on the mean start date. The mean start dates for *Quercus* range from February 11, at Houston, TX, to May 2, at Rochester, NY; this almost 3-month difference in the start date indicates that the regional differences in start date are large compared to the interannual variability and length of the season. Location is a very important factor in determining the start date of the season. Note that stations in the west coast (San Jose, CA, Eugene, OR, Seattle, WA) have an earlier start date than stations at the same latitude in the interior USA.

#### Duration of the main pollen season

**Fig. 8:**
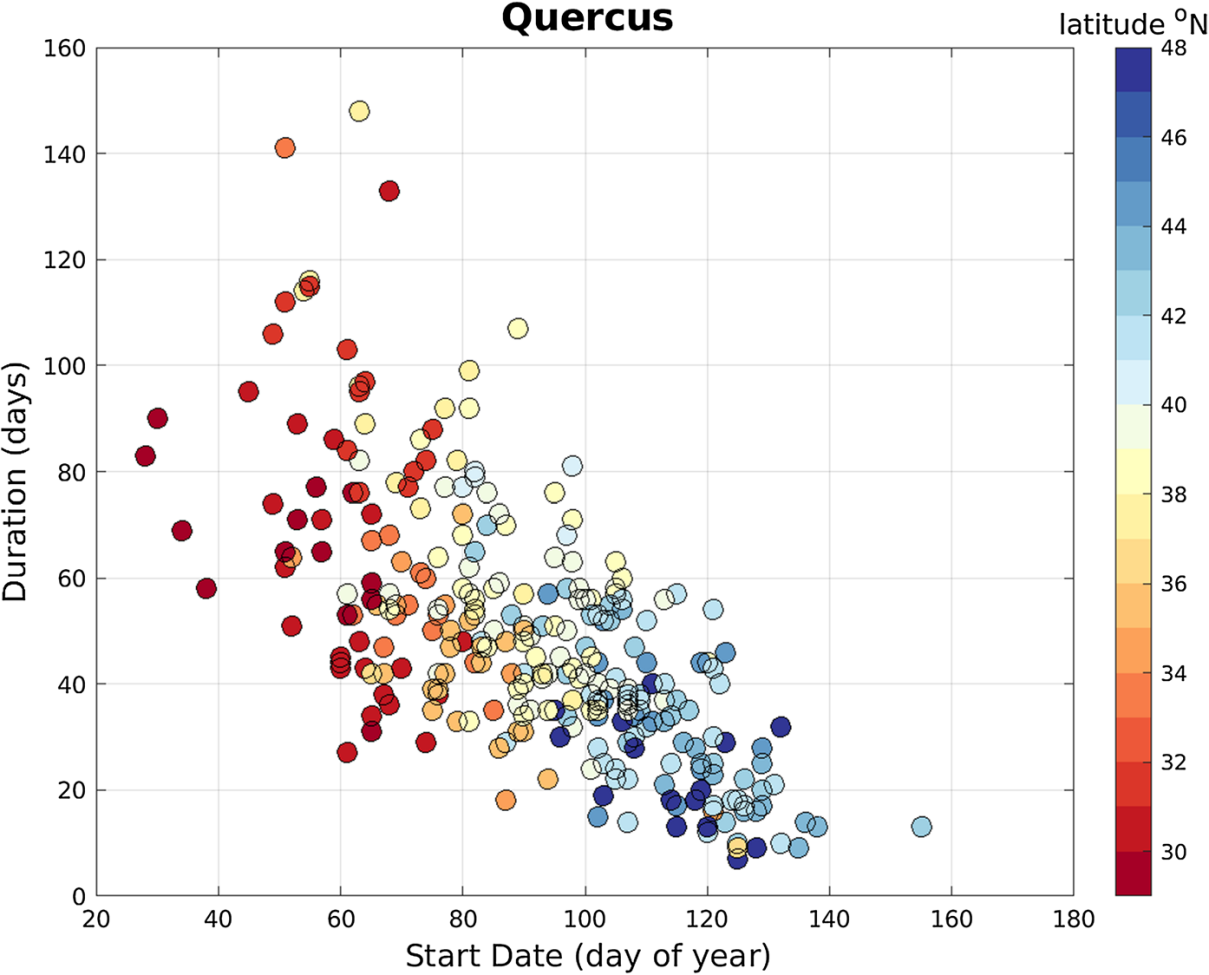
Scatter plot of start date of *Quercus* main pollen season with duration of *Quercus* main pollen season for all years for NAB stations. Colors indicate station’s latitude

The duration of the main pollen season also exhibits regional variation. Average *Quercus* season duration ranges from 23 days in Seattle, WA, to 103 days in San Jose, CA (Fig. 7b). In general, the duration of the season is also longer at lower latitudes for other taxa (not shown). The start date and duration of the *Quercus* main pollen season are significantly and negatively correlated at 99%, such that the higher-latitude stations have a later start date and a shorter season (Fig. 8). *Quercus* pollen is representative of the other allergenic tree pollen taxa in that they all have start dates significantly and negatively correlated with the duration of the main pollen season (not shown).

### Year-to-year variability in start date of the main pollen season

There is considerable interannual variability in main pollen season start dates. Figure 2 illustrates that start dates for six of the seven taxa from London, Ontario, can be assessed with reasonable reliability (see Sect. 3.3 for a discussion of the influence of first sampling date on the calculation of main pollen season start date). For these six taxa in London, standard deviation of start dates ranges from 3 to 13 days, with *Ambrosia* at the smallest and *Morus* and *Acer* at the largest.

The standard deviation of start date was calculated for each important allergenic pollen taxon for station-years with unbiased start dates. The standard deviation varies from 8.4 days for *Ambrosia* to 32.2 days for *Ulmus* pollen (Table 3). Larger variability is indicative of taxa with physiology that depends on many climatic factors such as temperature, precipitation, humidity, length of daylight, and soil moisture. Because the start of the *Ambrosia* main pollen season is strongly dependent on length of daylight, the start date for *Ambrosia* has relatively low variability compare to other pollen taxa.

**Table 3:** Standard deviation of start dates for important allergenic pollen taxa. Only years with reliable start dates from the 31 NAB stations are included

Taxa	Standard deviation of start date (days)	Number of station-years included
*Quercus*	11.8	232
*Cupressaceae*	25	63
*Ambrosia*	8.4	292
*Morus*	10.6	228
*Pinaceae*	13	230
*Ulmus*	32.2	112
*Fraxinus*	12.2	214
*Betula*	13.4	201
*Populus*	12.3	174
*Poaceae*	12.3	258
*Acer*	16.2	103

## Discussion

### Limitations and caveats

We have presented pollen calendars and analyses of pollen start dates and season duration. Our analyses are limited by the number of NAB stations available to us and by missing data. Only 31 of the 51 stations we received from NAB met our data inclusion criteria, thus limiting the spatial resolution of our analysis. Temporal limitations also limit our analyses: Many stations do not sample pollen every day and most do not sample year-round. The temporal limitations influenced our choice of start date definition: We chose a definition that was less sensitive to missing data. We are not able to draw conclusions related to the *Cupressaceae* pollen start date because it is likely that some stations do not begin sampling until after *Cupressaceae* pollen is already present in the atmosphere. This is unfortunate, as *Cupressaceae* pollen is widespread and is the second most abundant allergenic pollen in the USA. *Cupressaceae* was the only taxon to be clearly adversely affected by late sampling in this study. However, a trend of earlier start dates for many allergenic pollen taxa has been observed in past decades (van Vliet et al. 2002; Zhang et al. 2014b), and climate change is projected to continue to cause earlier start dates (van Vliet et al. 2002; Ariano et al. 2010; Galán et al. 2005; Garcia-Mozo et al. 2006). This trend in earlier start dates could potentially cause the sampling to begin too late to detect the start date of other allergenic taxa. Better monitoring and analysis of the main pollen season would be achieved with daily pollen concentration data collected year-round.

The NAB allows the use of two different pollen samplers, which sample pollen counts that are significantly correlated but not equal. Although the difference between the two is small and thought to have no clinical difference (Crisp et al. 2013), no quantitative comparison has been undertaken. In this study, we did use with reservation, the pollen data regardless of sampling method. In addition, lack of important station metadata makes it difficult to assess factors that could affect data quality; e.g., the Seattle station moved location in April 2, 2012 (Northwest Asthma & Allergy Center Web site) and the Atlanta station moved in June 2010 (e-mail communication with R. Panethere, Atlanta Allergy and Asthma Clinic, June 14, 2018), but these location changes are not noted in the NAB metadata. Local sources of pollen have a significant impact on sampled pollen, and a location change could produce different pollen concentrations (Sofiev et al. 2006).

The geographic distribution of the NAB stations is relatively sparse in the western half of the CUSSC region. This restricts our ability to analyze pollen characteristics on the continental scale. Pollen calendars are location dependent, so it is important to have enough stations to resolve the spatial variability of pollen. The general sparsity of western stations appears to have been worsened by a lower response rate to our data request among western stations. Requests for pollen data are brokered by the NAB, but each station has its own criteria for granting access, leading to different data coverage across data requests and applications that likely affect generalizability of research findings.

### Conclusions and recommendations

We have presented pollen calendars for four stations in the CUSSC region and created pollen calendars for the other 27 stations from the subset of the NAB dataset that met our data inclusion criteria (supplementary figures). Our focus is on documenting and characterizing the spatial and temporal structure of the main pollen season for allergenic pollen taxa across the CUSSC region. Our pollen calendars update and expand upon work done by Levetin (1998), Kosisky et al. (2010), and Zhang et al. (2014a). Pollen calendars are location dependent due to the regional nature of plant ecology and climate. A handful of allergenic taxa constitute the principal allergenic pollen load in most regions studied.

Despite the sparse spatial resolution of the NAB data, there is a clear latitudinal signal to the start date (e.g., for *Quercus* pollen, see Figs. 5a, 7a). Zhang et al. (2014b) observed the start dates in *Betula* and *Quercus* to be earlier at lower latitudes. We have expanded their study to include eleven important allergenic pollen taxa. The NAB stations show the same latitudinal dependence in all important allergenic tree pollen taxa with the exception of fall-pollinating *Ulmus*, which shows no latitude dependence. Latitude is a proxy for temperature and length of daylight. That said, the three stations on the west coast (San Jose, CA, Eugene, OR, and Seattle, WA) feature earlier start dates for tree pollen than is observed at other stations at the same latitude in the interior USA. This indicates that another factor, such as temperature, is influencing the start date. The west coast region is moderated by the transport of air from the Pacific Ocean and has a milder climate and warmer winters than the continental interior. Plant phenology, such as the time of pollen release, is highly dependent on temperature. Indeed, accumulated heat is used in many models that forecast main pollen season characteristics (Zhang et al. 2015; Galán et al. 1998; García-Mozo et al. 2008; Ritenberga et al. 2018).

The duration of the main pollen season for important allergenic pollen taxa is negatively correlated with the start date; hence, stations at lower latitudes with earlier start dates also have longer pollen seasons (e.g., see Fig. 8 for *Quercus*). The dependence of pollen season duration on latitude is less distinct than for start date, which suggests other environmental factors may contribute to the duration of the pollen season. Such factors could include weather prior to and during the pollen season: temperature, rain, wind, and sun. Other factors such as atmospheric carbon dioxide (CO_2_) concentration can affect the amount of pollen produced: *Ambrosia* has been observed to produce more pollen and more allergenic pollen under increased CO_2_ levels (Ziska et al. 2003; Ziska and Caulfield 2000; Singer et al. 2005).

It is often assumed that trees only release pollen in spring, grasses in summer, and weeds in fall. The pollen calendars for *Ulmus* (Fig. 5b) show that some species of *Ulmus* release pollen in the late summer and early fall; *Poaceae* pollen (Fig. 5d) is detected in the atmosphere from March through November; and *Cupressaceae* pollen (Fig. 4d and supplementary materials) can be found in atmosphere in the fall and winter. We can use these pollen calendars to help inform the allergy community to improve diagnosis and treatment.

The NAB pollen dataset has non-trivial amounts of missing data, and the number of stations is small in the western half of the CUSSC region. The limited spatiotemporal resolution of the pollen data affected the analyses we were able to conduct. Improvement in the spatiotemporal resolution of the data would lead to more complete analyses and a chance of better health outcomes for individuals with pollen allergies. We encourage the NAB to advocate for consistent year-round daily sampling of pollen concentrations and to increase the number of stations in the western CUSSC region.

### Electronic supplementary material

Below is the link to the electronic supplementary material.

Supplementary material 1 (PDF 12678 kb)

## NAB stations (professionals and clinics) used in this analysis

Stanley M Fineman, MD MBA FAAAAI, Atlanta Allergy and Asthma Clinic, Marietta (Atlanta), GA

Sheila Amar, MD, FAAAAI, FACAAI, Allergy and Asthma Center of Georgetown, Austin, TX

Jonathon Matz, MD, FAAAAI, and David Golden, MD, FAAAAI, Baltimore, MD

Linda Ford, MD, FAAAAI, The Asthma and Allergy Center, PC, Bellevue, NE

David Weldon, MD, FAAAAI, FACAAI, Scott and White Clinic, College Station, TX

Robert Nathan, MD, FAAAAI, and Daniel Soteres, MD, MPH, FAAAAI, Asthma and Allergy Associates, PC, Colorado Springs, CO

Donald Pulver, MD, FAAAAI, Allergy, Asthma and Immunology of Rochester, Rochester, NY

Andy Roth, RAPCA, Dayton, OH

Duane Harris, MD, FAAAAI, Intermountain Allergy and Asthma Clinic, Draper, UT

Philip Gallagher, MD, FAAAAI, Allergy and Asthma Associates of Northeastern Pennsylvania, Erie, PA

Kraig Jacobson, MD, FAAAAI, Allergy and Asthma Research Group, Eugene, OR

Neil Kao, MD, FAAAAI, Allergic Disease and Asthma Center, Greenville, SC

Tony Huynh, City of Houston, Houston, TX

Jay Portnoy, MD, FAAAAI, Children’s Mercy Hospital, Kansas City, MO

James Anderson, MLT, OSHTECH, London, ON

Robert Bush, MD, FAAAAI, University of Wisconsin Medical School, Madison, WI

Joseph Leija, MD, FAAAAI, Melrose Park, IL

Harold Kaiser, MD, FAAAAI, Clinical Research Institute, Minneapolis, MN

Warren Filley, MD, FAAAAI, OK Allergy Asthma Clinic, Inc., Oklahoma City, OK

Martha Tarpay, MD, Allergy and Asthma Center, Oklahoma City, OK

Wayne Wilhelm, Saint Louis County Health Department, St. Louis, MO

Robert Gomez, Wilford Hall Ambulatory Surgical Center, San Antonio, TX

Alan Goldsobel, MD, FAAAAI, and James Wolfe, MD, FAAAAI, Allergy and Asthma Associates of Northern California, San Jose, CA

Frank Virant, MD, FAAAAI, Northwest Asthma and Allergy Center, Seattle, WA

Rhizza Adams, Springfield-Greene County Health Department, Springfield, MO

James Love, Jr., MD, PhD, FAAAAI, Allergy Clinic of Tulsa, Tulsa, OK

Richard Henry, MD, Asthma and Allergy of Idaho, Twin Falls, ID

Pramila K. Daftary, MD, FAAAAI, Allergy and Asthma Care of Waco, Waco, TX

Susan E. Kosisky, MHA, US Army Garrison-Forest Glen, Silver Spring, MD (Washington, DC)

Christopher Randolf, MD, FAAAAI, Waterbury, CT

Michael Nickels, MD, PhD, Allergy and Asthma Consultants, Inc., York, PA
