# Supplementary material for: Pollen calendars and maps of allergenic pollen in North America
Source: Aerobiologia (Bologna). 2019 Jul 17;35(4):613–33. doi: 10.1007/s10453-019-09601-2 (PMC6934246; doi:10.1007/s10453-019-09601-2)
Supplement: Supplementary file 1 — Supplementary material 1 (PDF 12678 kb) [file 10453_2019_9601_MOESM1_ESM.pdf]

Location-specific Pollen Calendars

Pollen calendars for the station at the city and state or province listed in title of figure. Daily mean climatology of pollen concentration by pollen taxa, 2003-2017. Percent abundance is based on the sum of API over all pollen taxa. Only pollen taxa with average API over 150 grain-day/m3 are shown. Missing data are shaded grey and denoted NaN in the colorbar.

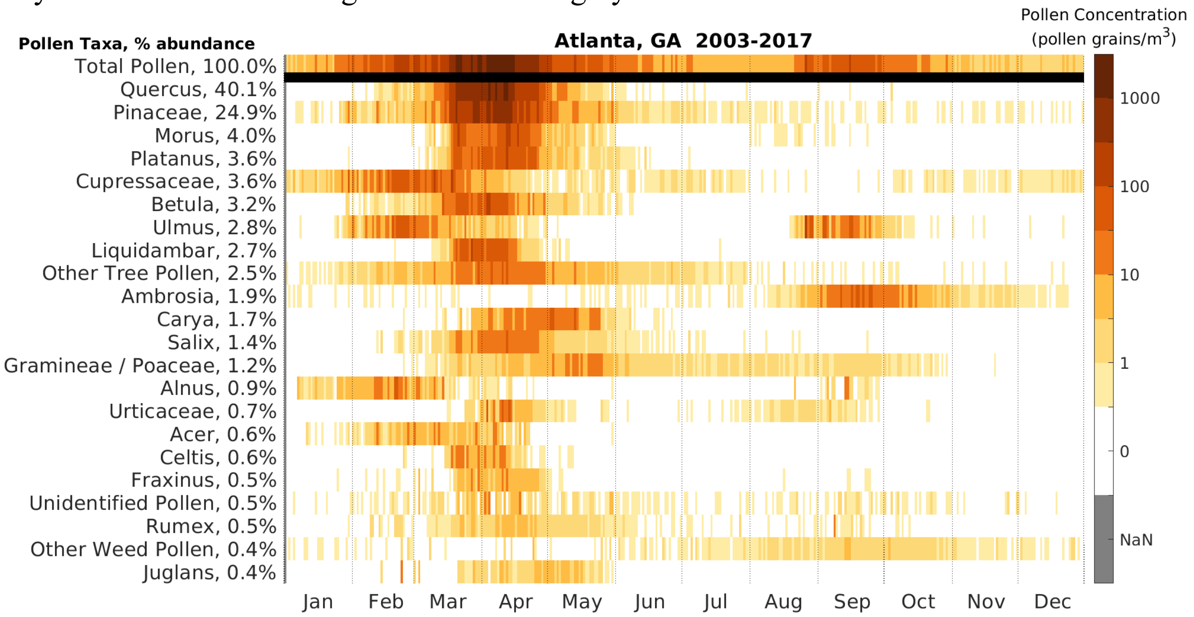

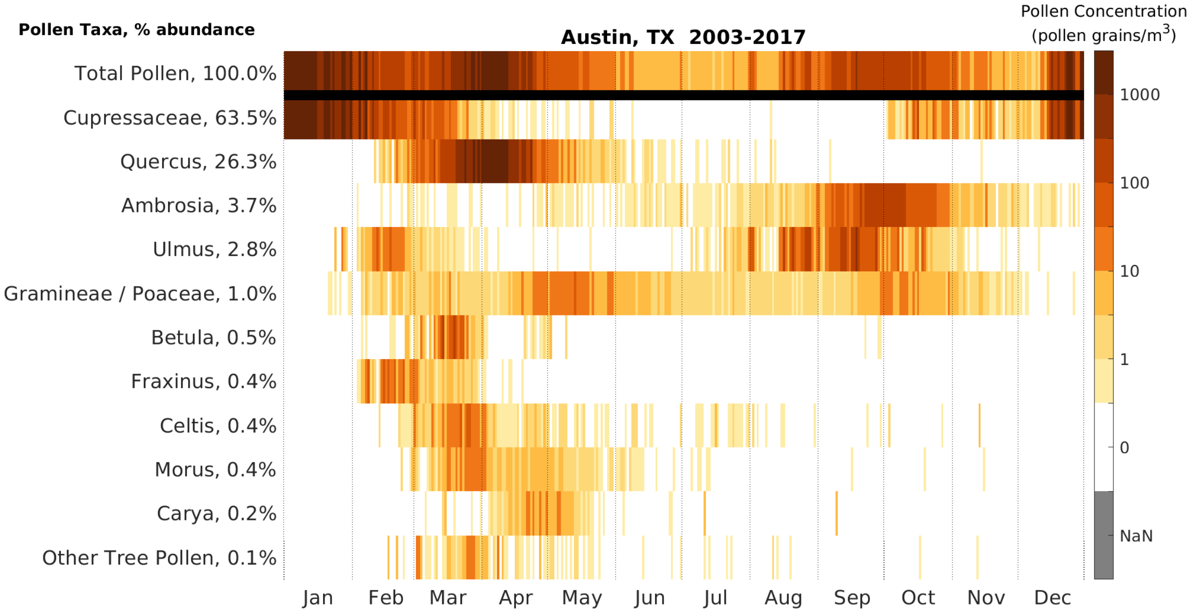

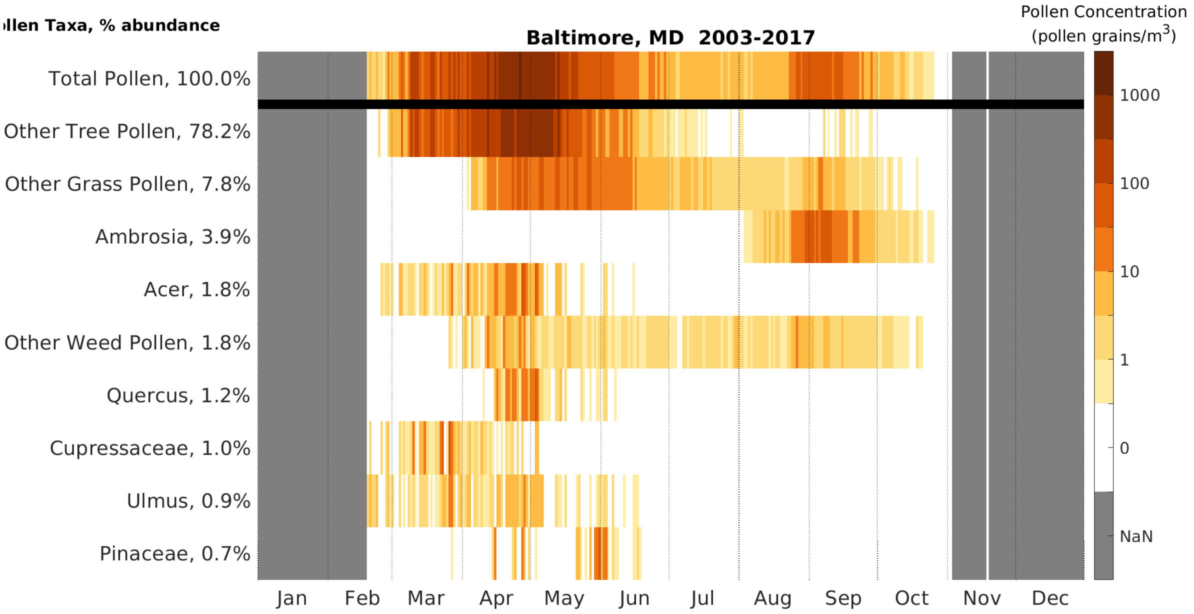

llen Taxa, % abundance
Baltimore, MD 2003-2017
Pollen Concentration
(pollen grains/m3)
Total Pollen, 100.0%
Other Tree Pollen, 78.2%
Other Grass Pollen, 7.8%
Ambrosia, 3.9%
Acer, 1.8%
Other Weed Pollen, 1.8%
Quercus, 1.2%
Cupressaceae, 1.0%
Ulmus, 0.9%
Pinaceae, 0.7%
Jan Feb Mar Apr May Jun Jul Aug Sep Oct Nov Dec
1000
100
10
1
0
NaN

9

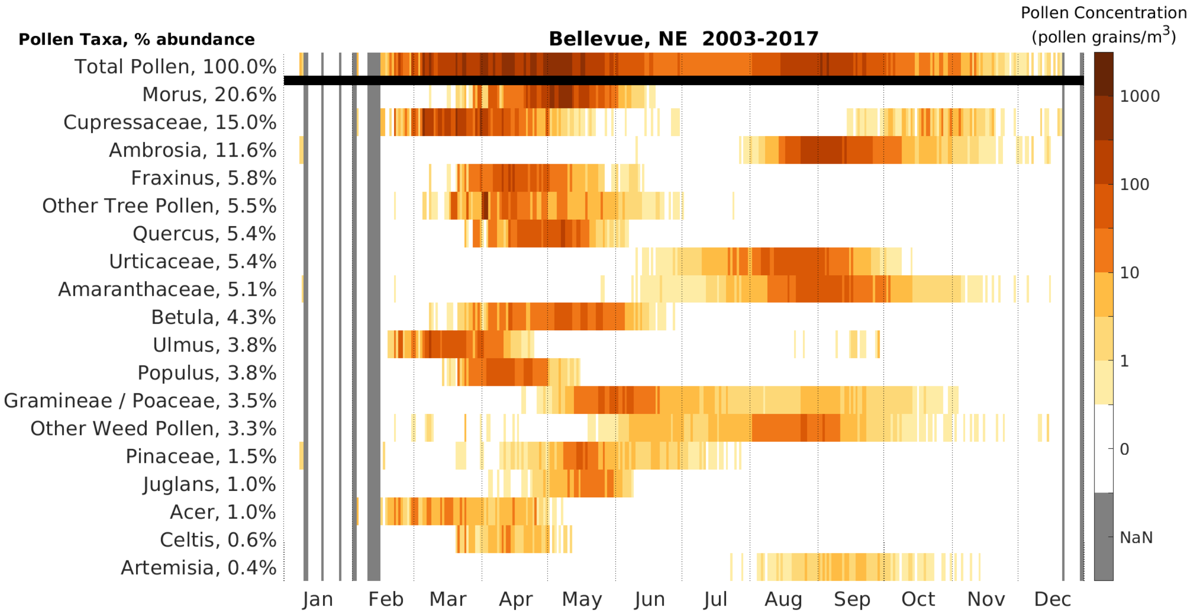

Pollen Taxa, % abundance
Bellevue, NE 2003-2017
Pollen Concentration
(pollen grains/m3)
Total Pollen, 100.0%
Morus, 20.6%
Cupressaceae, 15.0%
Ambrosia, 11.6%
Fraxinus, 5.8%
Other Tree Pollen, 5.5%
Quercus, 5.4%
Urticaceae, 5.4%
Amaranthaceae, 5.1%
Betula, 4.3%
Ulmus, 3.8%
Populus, 3.8%
Gramineae / Poaceae, 3.5%
Other Weed Pollen, 3.3%
Pinaceae, 1.5%
Juglans, 1.0%
Acer, 1.0%
Celtis, 0.6%
Artemisia, 0.4%
Jan Feb Mar Apr May Jun Jul Aug Sep Oct Nov Dec
1000
100
10
1
0
NaN

10

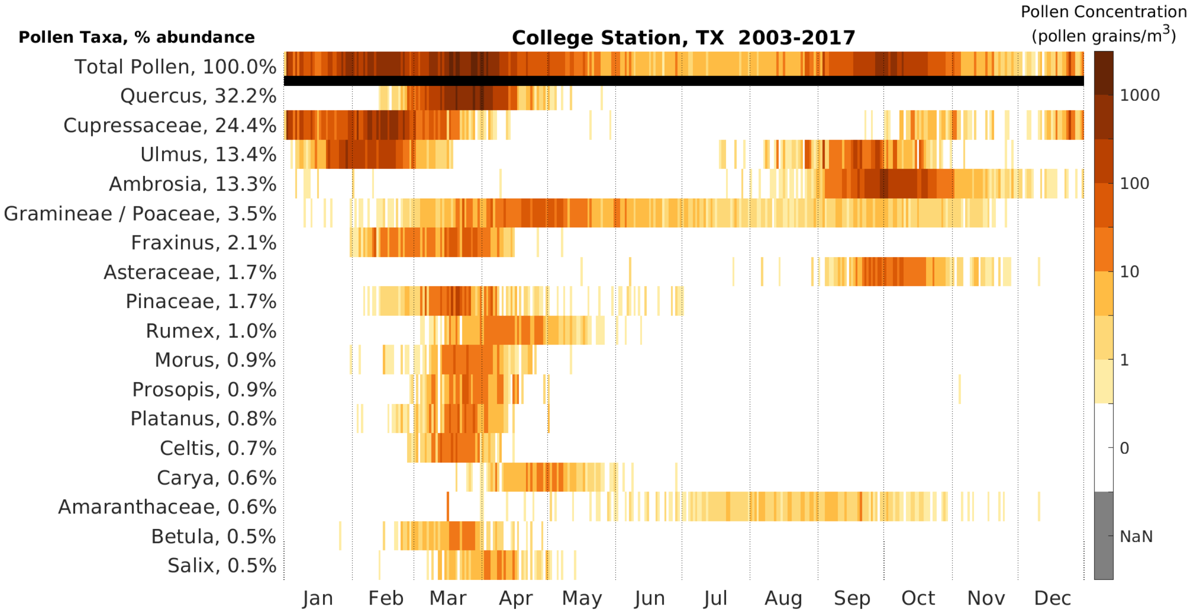
Pollen Taxa, % abundance
College Station, TX 2003-2017
Pollen Concentration (pollen grains/m3)
Total Pollen, 100.0%
Quercus, 32.2%
Cupressaceae, 24.4%
Ulmus, 13.4%
Ambrosia, 13.3%
Gramineae / Poaceae, 3.5%
Fraxinus, 2.1%
Asteraceae, 1.7%
Pinaceae, 1.7%
Rumex, 1.0%
Morus, 0.9%
Prosopis, 0.9%
Platanus, 0.8%
Celtis, 0.7%
Carya, 0.6%
Amaranthaceae, 0.6%
Betula, 0.5%
Salix, 0.5%
Jan Feb Mar Apr May Jun Jul Aug Sep Oct Nov Dec
1000
100
10
1
0
NaN

11

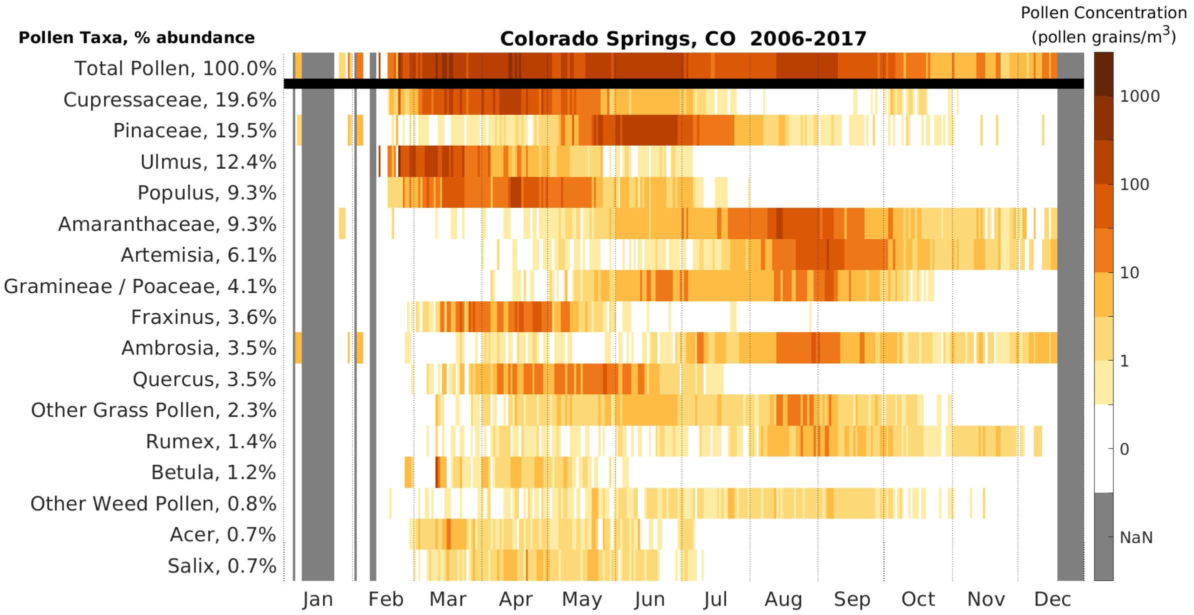
Pollen Taxa, % abundance
Colorado Springs, CO 2006-2017
Pollen Concentration (pollen grains/m3)
Total Pollen, 100.0%
Cupressaceae, 19.6%
Pinaceae, 19.5%
Ulmus, 12.4%
Populus, 9.3%
Amaranthaceae, 9.3%
Artemisia, 6.1%
Gramineae / Poaceae, 4.1%
Fraxinus, 3.6%
Ambrosia, 3.5%
Quercus, 3.5%
Other Grass Pollen, 2.3%
Rumex, 1.4%
Betula, 1.2%
Other Weed Pollen, 0.8%
Acer, 0.7%
Salix, 0.7%
Jan Feb Mar Apr May Jun Jul Aug Sep Oct Nov Dec
1000
100
10
1
0
NaN

12

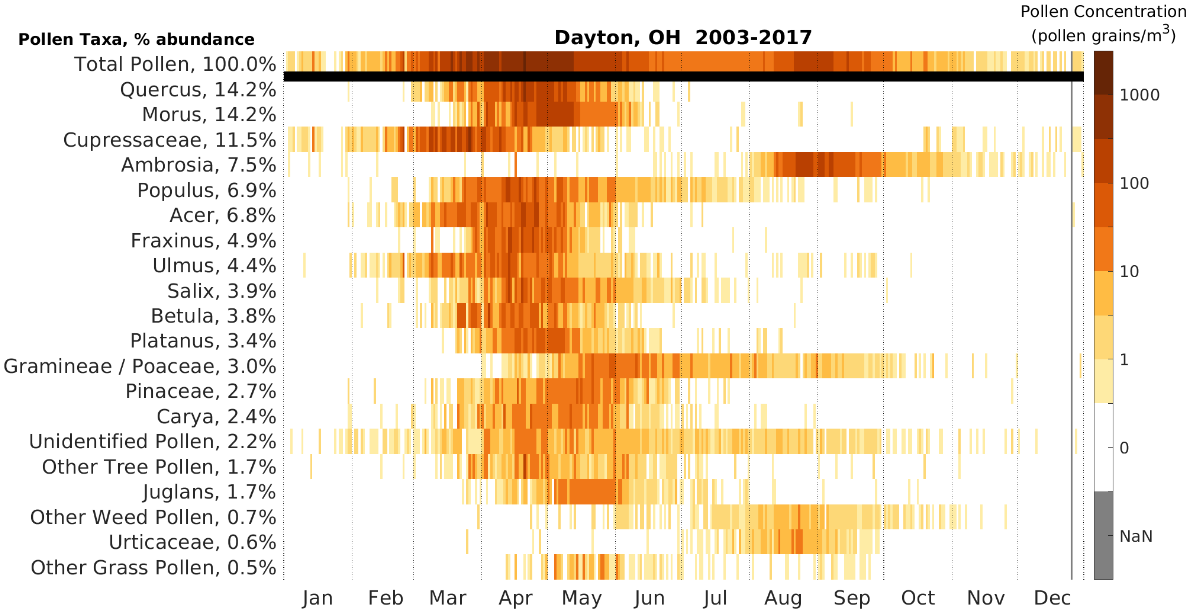

Pollen Taxa, % abundance
Dayton, OH 2003-2017
Pollen Concentration
(pollen grains/m3)
Total Pollen, 100.0%
Quercus, 14.2%
Morus, 14.2%
Cupressaceae, 11.5%
Ambrosia, 7.5%
Populus, 6.9%
Acer, 6.8%
Fraxinus, 4.9%
Ulmus, 4.4%
Salix, 3.9%
Betula, 3.8%
Platanus, 3.4%
Gramineae / Poaceae, 3.0%
Pinaceae, 2.7%
Carya, 2.4%
Unidentified Pollen, 2.2%
Other Tree Pollen, 1.7%
Juglans, 1.7%
Other Weed Pollen, 0.7%
Urticaceae, 0.6%
Other Grass Pollen, 0.5%
Jan Feb Mar Apr May Jun Jul Aug Sep Oct Nov Dec
1000
100
10
1
0
NaN

13

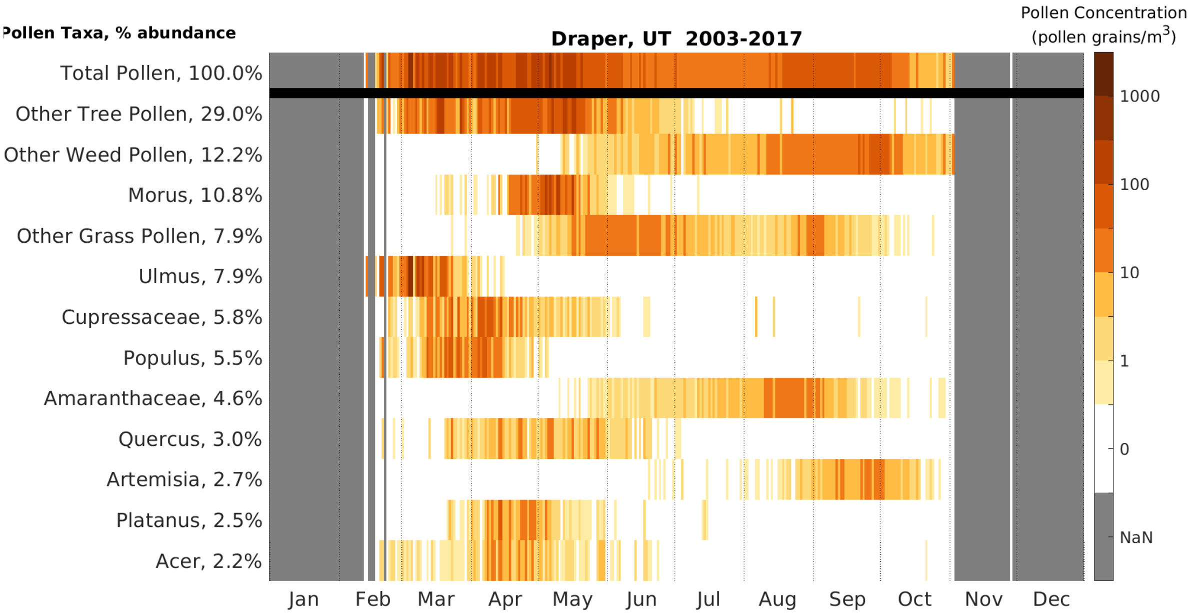

Pollen Taxa, % abundance
Draper, UT 2003-2017
Pollen Concentration
(pollen grains/m3)
Total Pollen, 100.0%
Other Tree Pollen, 29.0%
Other Weed Pollen, 12.2%
Morus, 10.8%
Other Grass Pollen, 7.9%
Ulmus, 7.9%
Cupressaceae, 5.8%
Populus, 5.5%
Amaranthaceae, 4.6%
Quercus, 3.0%
Artemisia, 2.7%
Platanus, 2.5%
Acer, 2.2%
Jan Feb Mar Apr May Jun Jul Aug Sep Oct Nov Dec
1000
100
10
1
0
NaN

14

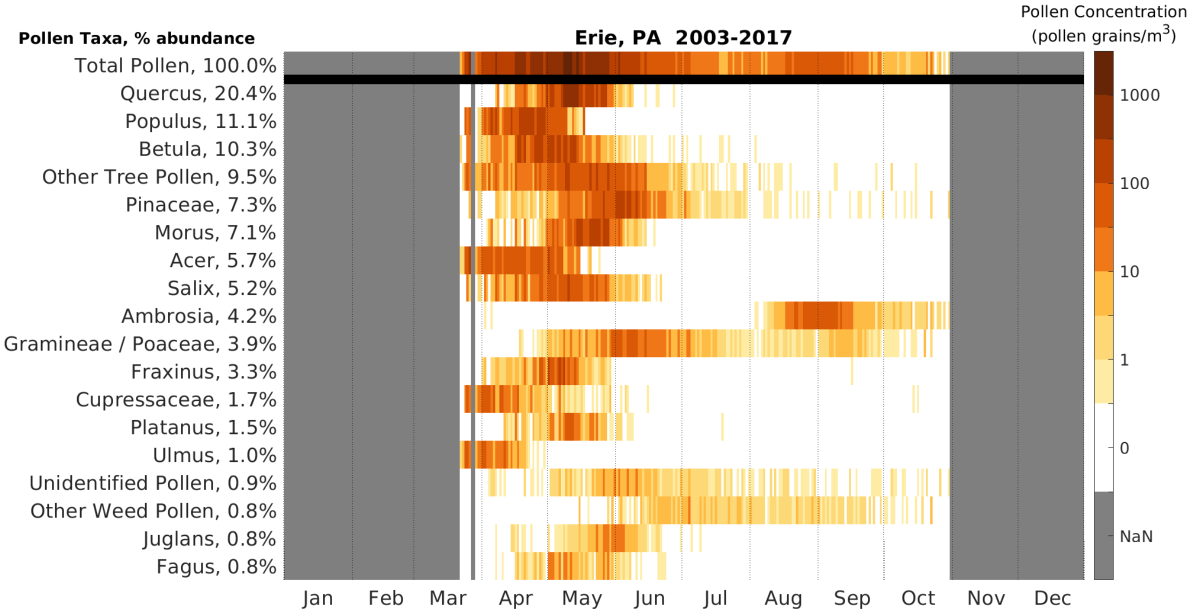
Pollen Taxa, % abundance
Erie, PA 2003-2017
Pollen Concentration (pollen grains/m^3)
Total Pollen, 100.0%
Quercus, 20.4%
Populus, 11.1%
Betula, 10.3%
Other Tree Pollen, 9.5%
Pinaceae, 7.3%
Morus, 7.1%
Acer, 5.7%
Salix, 5.2%
Ambrosia, 4.2%
Gramineae / Poaceae, 3.9%
Fraxinus, 3.3%
Cupressaceae, 1.7%
Platanus, 1.5%
Ulmus, 1.0%
Unidentified Pollen, 0.9%
Other Weed Pollen, 0.8%
Juglans, 0.8%
Fagus, 0.8%
Jan Feb Mar Apr May Jun Jul Aug Sep Oct Nov Dec
1000
100
10
1
0
NaN

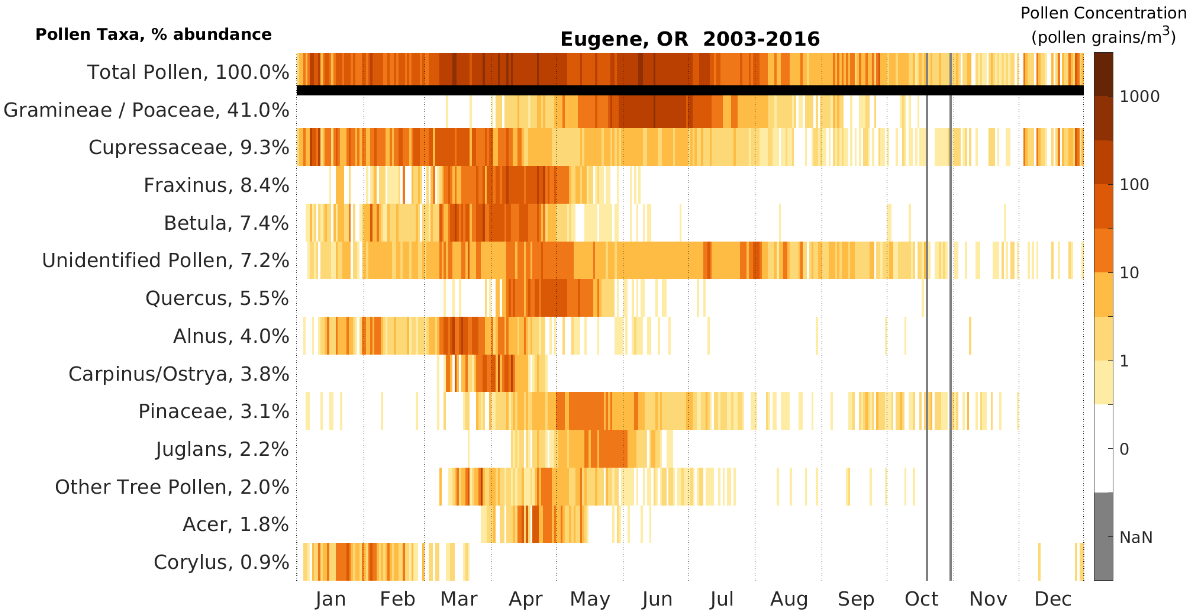
Pollen Taxa, % abundance
Eugene, OR 2003-2016
Pollen Concentration (pollen grains/m^3)
Total Pollen, 100.0%
Gramineae / Poaceae, 41.0%
Cupressaceae, 9.3%
Fraxinus, 8.4%
Betula, 7.4%
Unidentified Pollen, 7.2%
Quercus, 5.5%
Alnus, 4.0%
Carpinus/Ostrya, 3.8%
Pinaceae, 3.1%
Juglans, 2.2%
Other Tree Pollen, 2.0%
Acer, 1.8%
Corylus, 0.9%
Jan Feb Mar Apr May Jun Jul Aug Sep Oct Nov Dec
1000
100
10
1
0
NaN

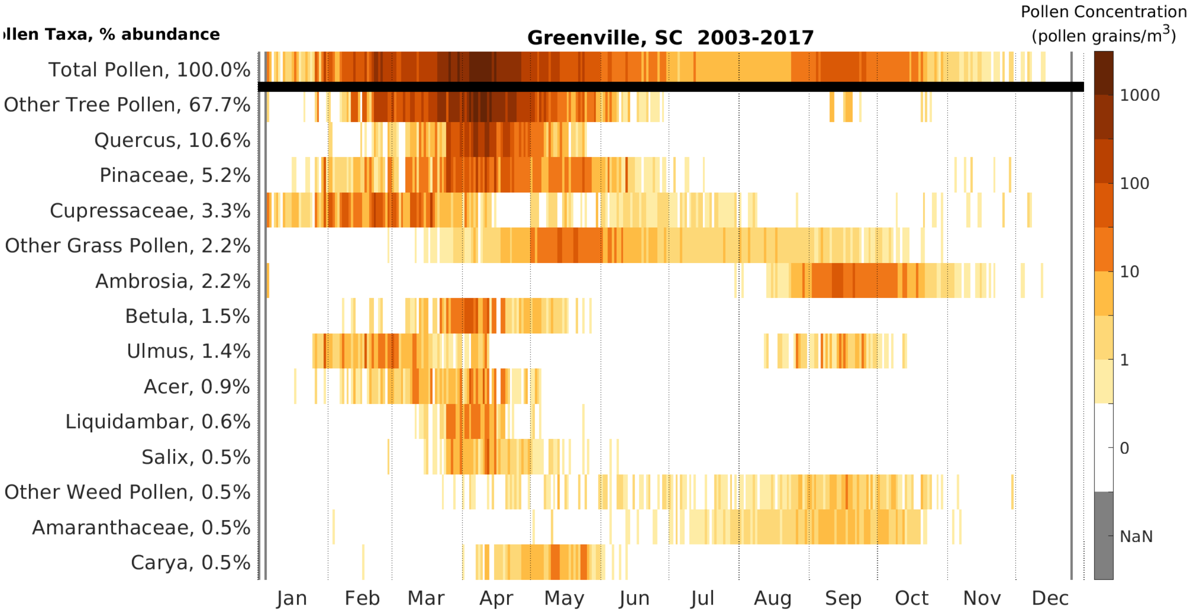
llen Taxa, % abundance
Greenville, SC 2003-2017
Pollen Concentration
(pollen grains/m3)
Total Pollen, 100.0%
Other Tree Pollen, 67.7%
Quercus, 10.6%
Pinaceae, 5.2%
Cupressaceae, 3.3%
Other Grass Pollen, 2.2%
Ambrosia, 2.2%
Betula, 1.5%
Ulmus, 1.4%
Acer, 0.9%
Liquidambar, 0.6%
Salix, 0.5%
Other Weed Pollen, 0.5%
Amaranthaceae, 0.5%
Carya, 0.5%
Jan
Feb
Mar
Apr
May
Jun
Jul
Aug
Sep
Oct
Nov
Dec
1000
100
10
1
0
NaN

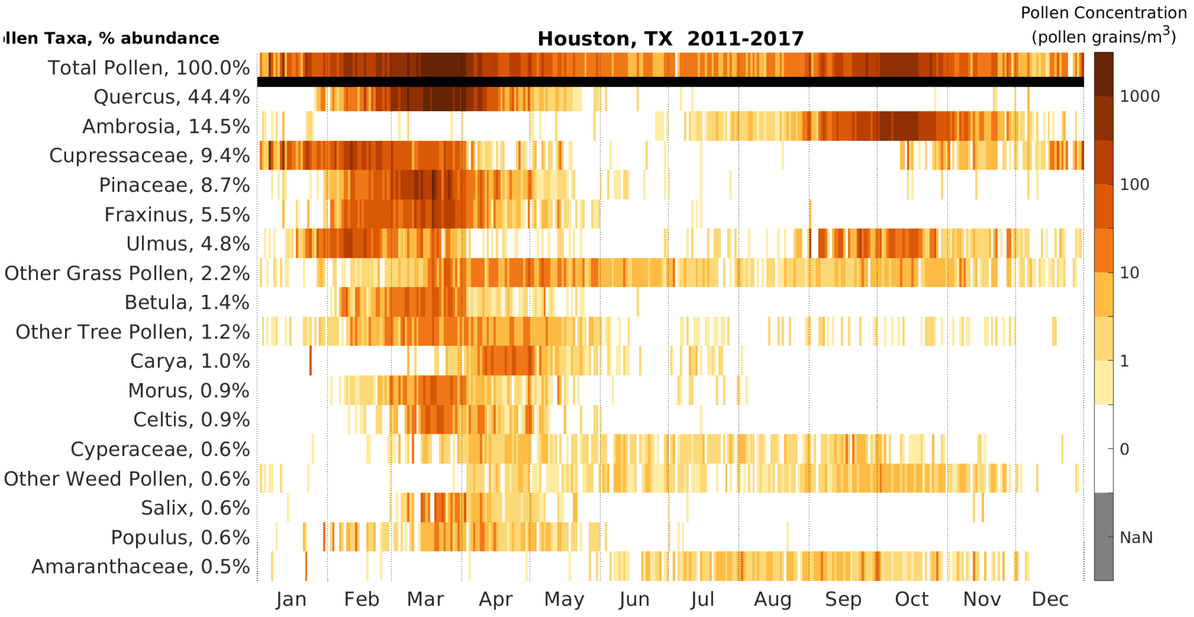
llen Taxa, % abundance
Houston, TX 2011-2017
Pollen Concentration
(pollen grains/m3)
Total Pollen, 100.0%
Quercus, 44.4%
Ambrosia, 14.5%
Cupressaceae, 9.4%
Pinaceae, 8.7%
Fraxinus, 5.5%
Ulmus, 4.8%
Other Grass Pollen, 2.2%
Betula, 1.4%
Other Tree Pollen, 1.2%
Carya, 1.0%
Morus, 0.9%
Celtis, 0.9%
Cyperaceae, 0.6%
Other Weed Pollen, 0.6%
Salix, 0.6%
Populus, 0.6%
Amaranthaceae, 0.5%
Jan
Feb
Mar
Apr
May
Jun
Jul
Aug
Sep
Oct
Nov
Dec
1000
100
10
1
0
NaN

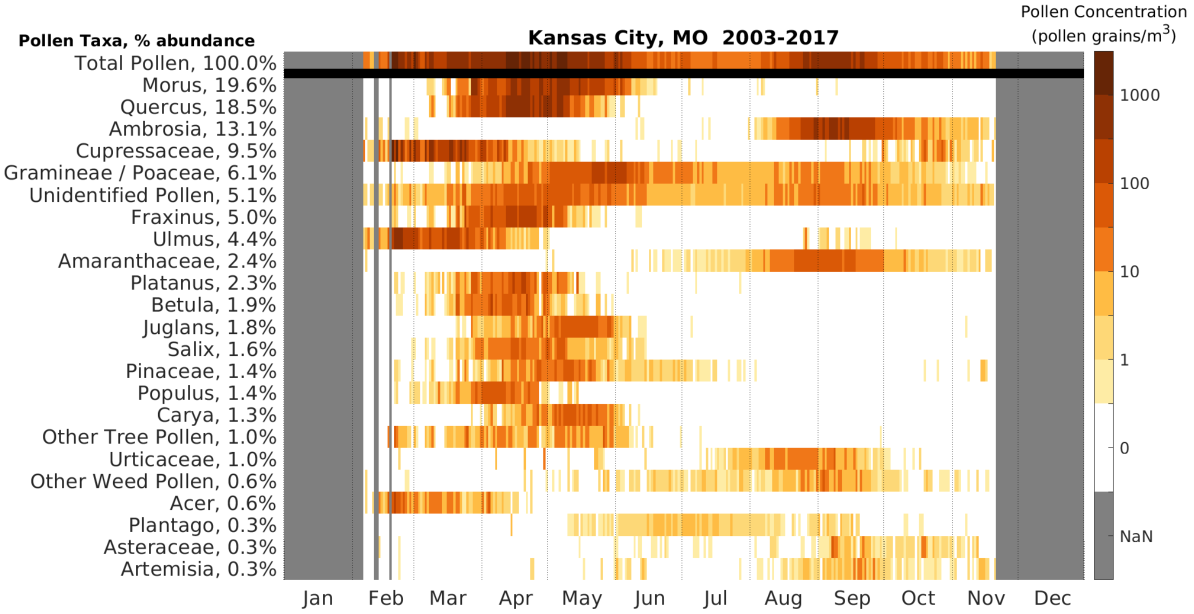

Pollen Taxa, % abundance
Kansas City, MO 2003-2017
Pollen Concentration (pollen grains/m^3)
Total Pollen, 100.0%
Morus, 19.6%
Quercus, 18.5%
Ambrosia, 13.1%
Cupressaceae, 9.5%
Gramineae / Poaceae, 6.1%
Unidentified Pollen, 5.1%
Fraxinus, 5.0%
Ulmus, 4.4%
Amaranthaceae, 2.4%
Platanus, 2.3%
Betula, 1.9%
Juglans, 1.8%
Salix, 1.6%
Pinaceae, 1.4%
Populus, 1.4%
Carya, 1.3%
Other Tree Pollen, 1.0%
Urticaceae, 1.0%
Other Weed Pollen, 0.6%
Acer, 0.6%
Plantago, 0.3%
Asteraceae, 0.3%
Artemisia, 0.3%
Jan Feb Mar Apr May Jun Jul Aug Sep Oct Nov Dec
1000
100
10
1
0
NaN

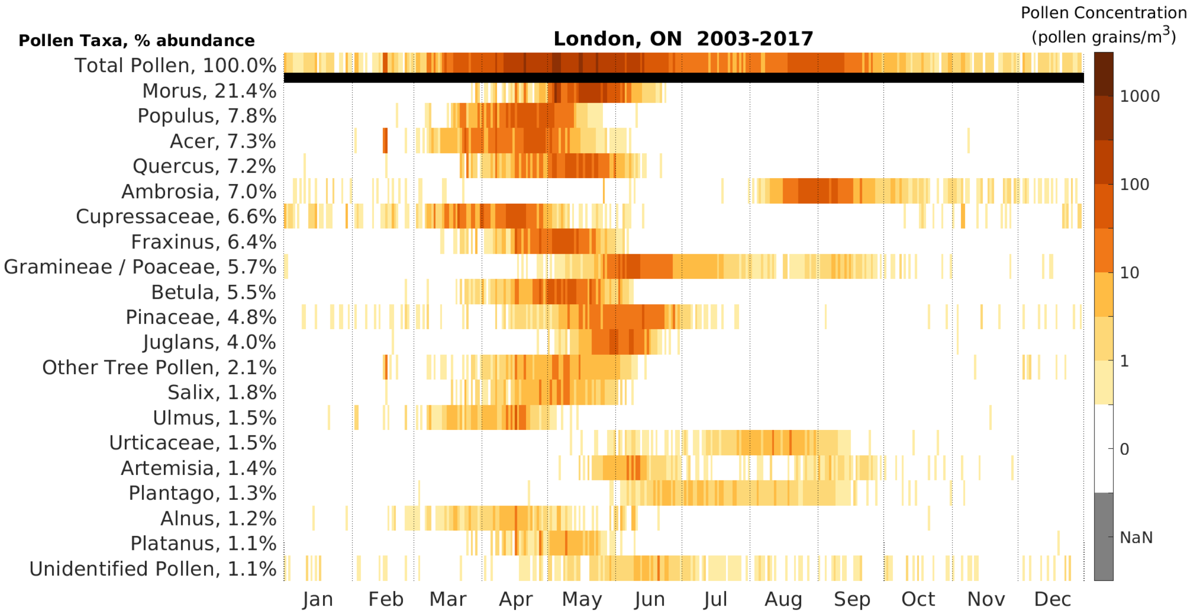

Pollen Taxa, % abundance
London, ON 2003-2017
Pollen Concentration (pollen grains/m^3)
Total Pollen, 100.0%
Morus, 21.4%
Populus, 7.8%
Acer, 7.3%
Quercus, 7.2%
Ambrosia, 7.0%
Cupressaceae, 6.6%
Fraxinus, 6.4%
Gramineae / Poaceae, 5.7%
Betula, 5.5%
Pinaceae, 4.8%
Juglans, 4.0%
Other Tree Pollen, 2.1%
Salix, 1.8%
Ulmus, 1.5%
Urticaceae, 1.5%
Artemisia, 1.4%
Plantago, 1.3%
Alnus, 1.2%
Platanus, 1.1%
Unidentified Pollen, 1.1%
Jan Feb Mar Apr May Jun Jul Aug Sep Oct Nov Dec
1000
100
10
1
0
NaN

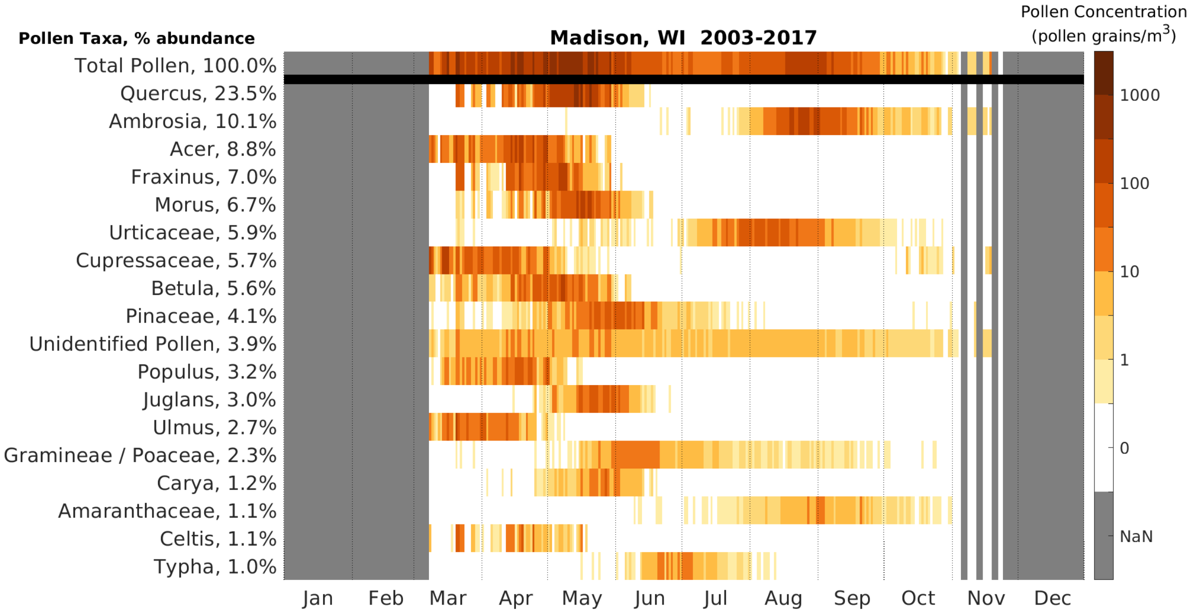

Madison, WI 2003-2017
Pollen Taxa, % abundance
Total Pollen, 100.0%
Quercus, 23.5%
Ambrosia, 10.1%
Acer, 8.8%
Fraxinus, 7.0%
Morus, 6.7%
Urticaceae, 5.9%
Cupressaceae, 5.7%
Betula, 5.6%
Pinaceae, 4.1%
Unidentified Pollen, 3.9%
Populus, 3.2%
Juglans, 3.0%
Ulmus, 2.7%
Gramineae / Poaceae, 2.3%
Carya, 1.2%
Amaranthaceae, 1.1%
Celtis, 1.1%
Typha, 1.0%
Jan Feb Mar Apr May Jun Jul Aug Sep Oct Nov Dec
Pollen Concentration (pollen grains/m³)
1000
100
10
1
0
NaN

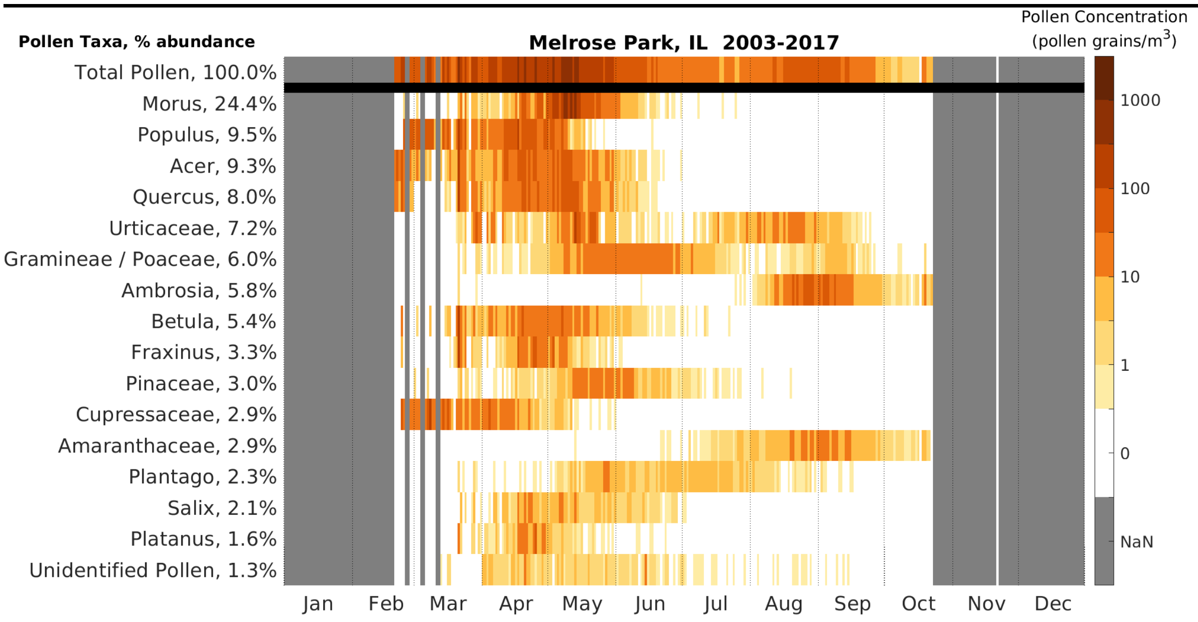

Melrose Park, IL 2003-2017
Pollen Taxa, % abundance
Total Pollen, 100.0%
Morus, 24.4%
Populus, 9.5%
Acer, 9.3%
Quercus, 8.0%
Urticaceae, 7.2%
Gramineae / Poaceae, 6.0%
Ambrosia, 5.8%
Betula, 5.4%
Fraxinus, 3.3%
Pinaceae, 3.0%
Cupressaceae, 2.9%
Amaranthaceae, 2.9%
Plantago, 2.3%
Salix, 2.1%
Platanus, 1.6%
Unidentified Pollen, 1.3%
Jan Feb Mar Apr May Jun Jul Aug Sep Oct Nov Dec
Pollen Concentration (pollen grains/m³)
1000
100
10
1
0
NaN

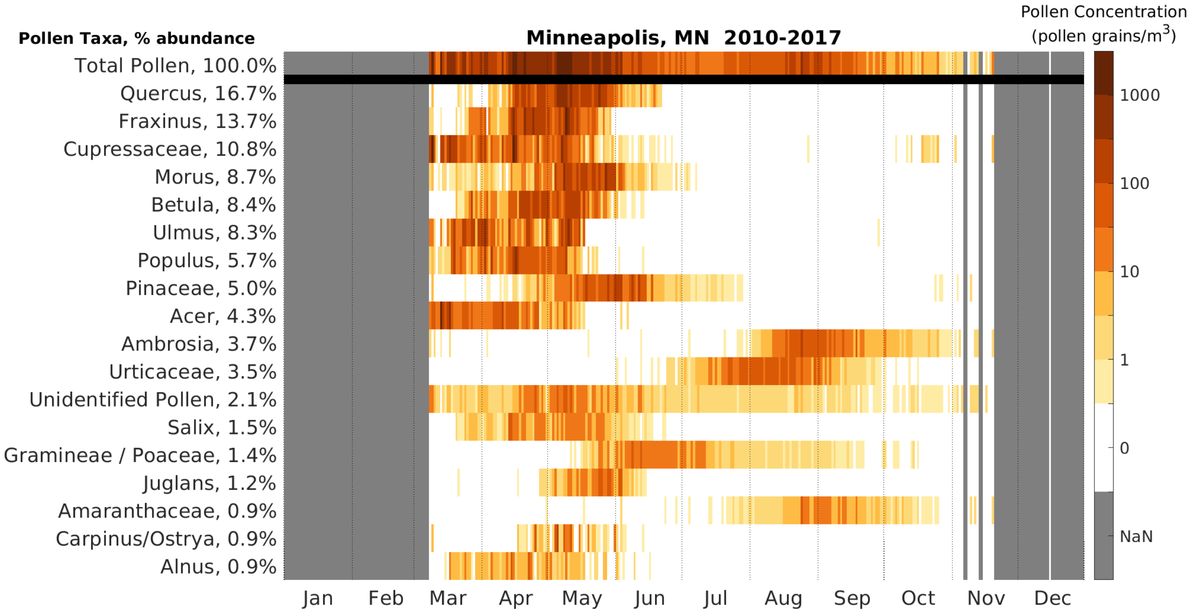
Minneapolis, MN 2010-2017
Pollen Taxa, % abundance
Total Pollen, 100.0%
Quercus, 16.7%
Fraxinus, 13.7%
Cupressaceae, 10.8%
Morus, 8.7%
Betula, 8.4%
Ulmus, 8.3%
Populus, 5.7%
Pinaceae, 5.0%
Acer, 4.3%
Ambrosia, 3.7%
Urticaceae, 3.5%
Unidentified Pollen, 2.1%
Salix, 1.5%
Gramineae / Poaceae, 1.4%
Juglans, 1.2%
Amaranthaceae, 0.9%
Carpinus/Ostrya, 0.9%
Alnus, 0.9%
Jan
Feb
Mar
Apr
May
Jun
Jul
Aug
Sep
Oct
Nov
Dec
Pollen Concentration
(pollen grains/m3)
1000
100
10
1
0
NaN

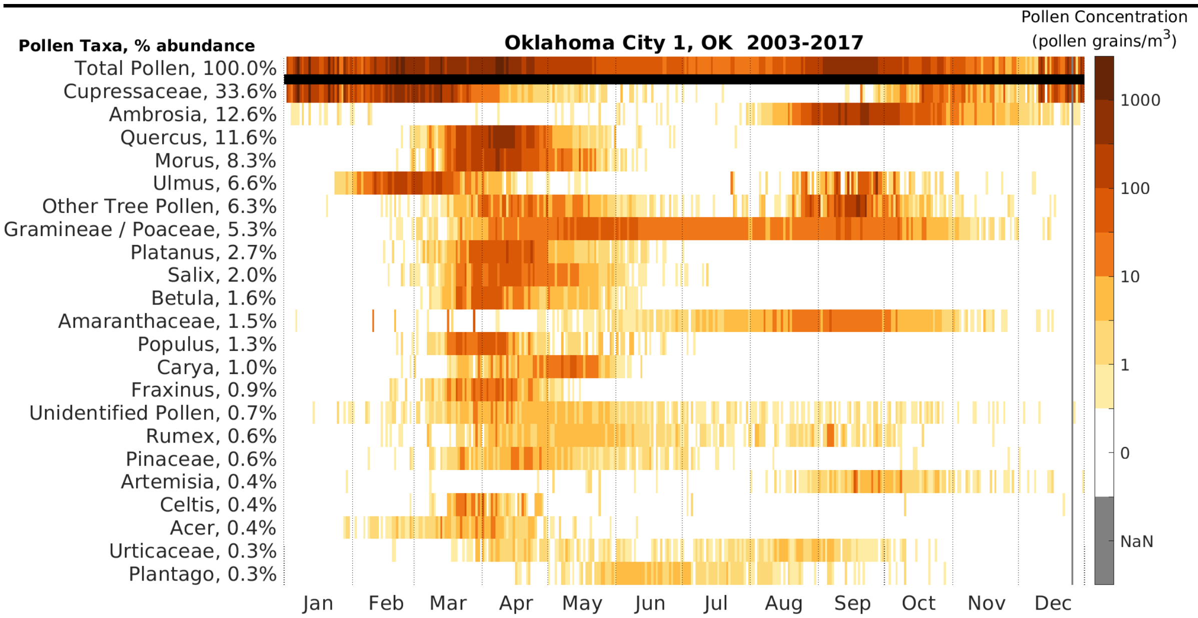
Oklahoma City 1, OK 2003-2017
Pollen Taxa, % abundance
Total Pollen, 100.0%
Cupressaceae, 33.6%
Ambrosia, 12.6%
Quercus, 11.6%
Morus, 8.3%
Ulmus, 6.6%
Other Tree Pollen, 6.3%
Gramineae / Poaceae, 5.3%
Platanus, 2.7%
Salix, 2.0%
Betula, 1.6%
Amaranthaceae, 1.5%
Populus, 1.3%
Carya, 1.0%
Fraxinus, 0.9%
Unidentified Pollen, 0.7%
Rumex, 0.6%
Pinaceae, 0.6%
Artemisia, 0.4%
Celtis, 0.4%
Acer, 0.4%
Urticaceae, 0.3%
Plantago, 0.3%
Jan
Feb
Mar
Apr
May
Jun
Jul
Aug
Sep
Oct
Nov
Dec
Pollen Concentration
(pollen grains/m3)
1000
100
10
1
0
NaN

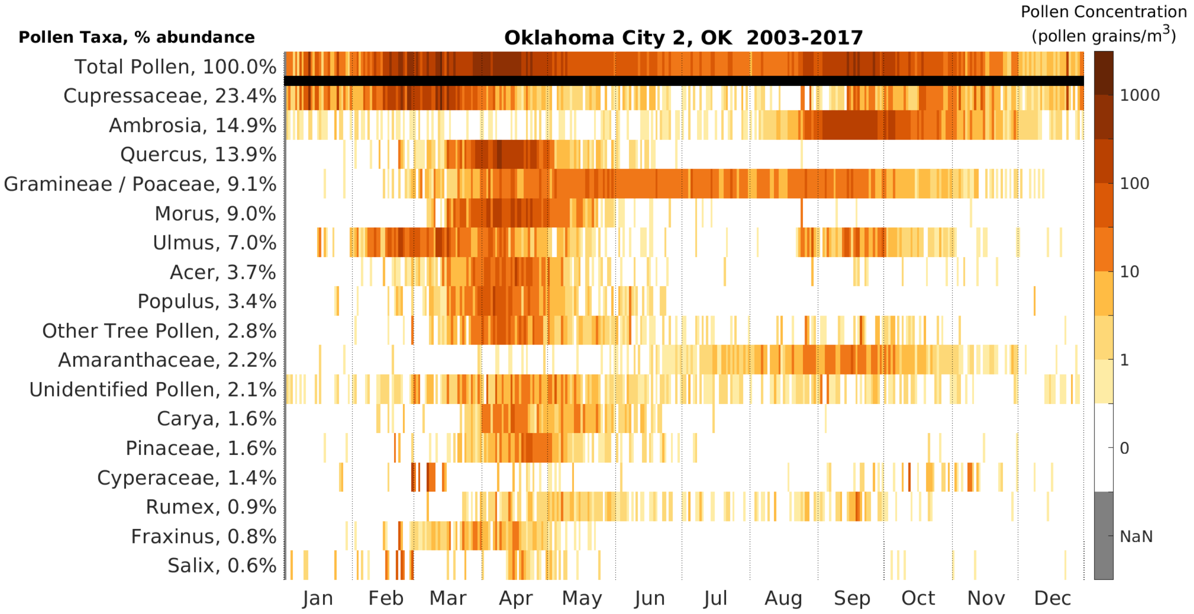
Pollen Taxa, % abundance
Oklahoma City 2, OK 2003-2017
Pollen Concentration
(pollen grains/m3)
Total Pollen, 100.0%
Cupressaceae, 23.4%
Ambrosia, 14.9%
Quercus, 13.9%
Gramineae / Poaceae, 9.1%
Morus, 9.0%
Ulmus, 7.0%
Acer, 3.7%
Populus, 3.4%
Other Tree Pollen, 2.8%
Amaranthaceae, 2.2%
Unidentified Pollen, 2.1%
Carya, 1.6%
Pinaceae, 1.6%
Cyperaceae, 1.4%
Rumex, 0.9%
Fraxinus, 0.8%
Salix, 0.6%
Jan Feb Mar Apr May Jun Jul Aug Sep Oct Nov Dec
1000
100
10
1
0
NaN

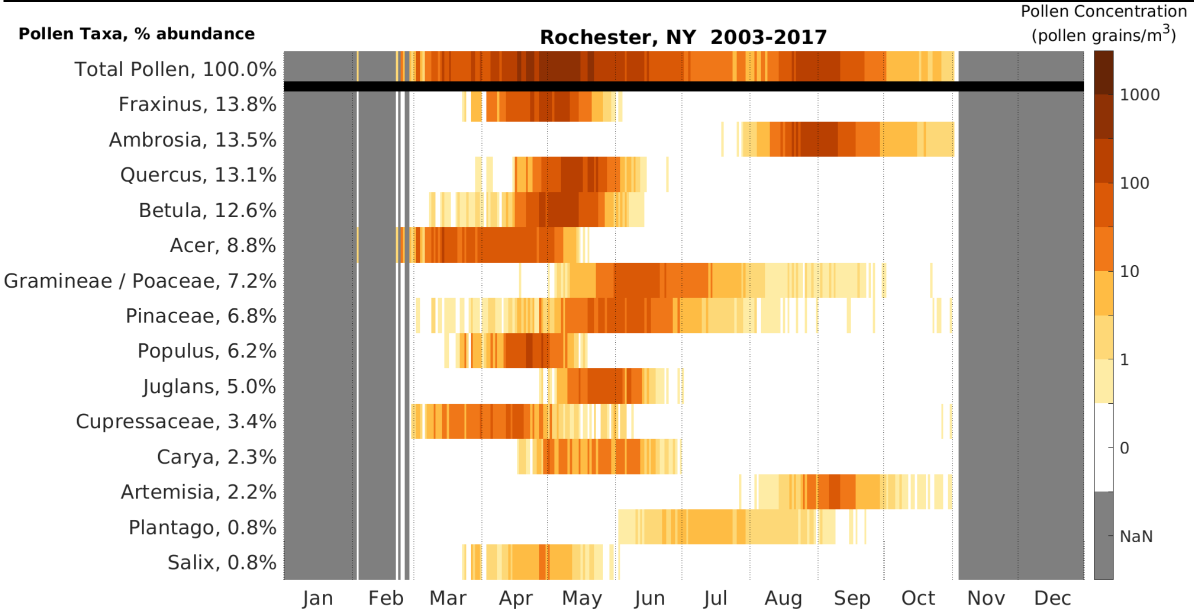
Pollen Taxa, % abundance
Rochester, NY 2003-2017
Pollen Concentration
(pollen grains/m3)
Total Pollen, 100.0%
Fraxinus, 13.8%
Ambrosia, 13.5%
Quercus, 13.1%
Betula, 12.6%
Acer, 8.8%
Gramineae / Poaceae, 7.2%
Pinaceae, 6.8%
Populus, 6.2%
Juglans, 5.0%
Cupressaceae, 3.4%
Carya, 2.3%
Artemisia, 2.2%
Plantago, 0.8%
Salix, 0.8%
Jan Feb Mar Apr May Jun Jul Aug Sep Oct Nov Dec
1000
100
10
1
0
NaN

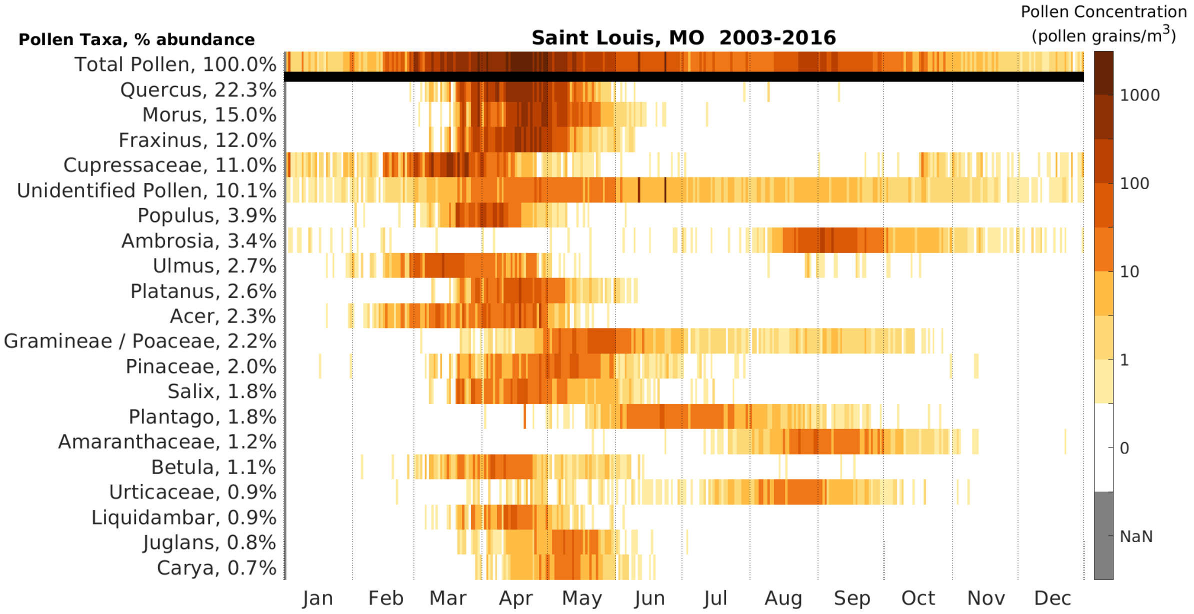
Pollen Taxa, % abundance
Saint Louis, MO 2003-2016
Pollen Concentration
(pollen grains/m3)
Total Pollen, 100.0%
Quercus, 22.3%
Morus, 15.0%
Fraxinus, 12.0%
Cupressaceae, 11.0%
Unidentified Pollen, 10.1%
Populus, 3.9%
Ambrosia, 3.4%
Ulmus, 2.7%
Platanus, 2.6%
Acer, 2.3%
Gramineae / Poaceae, 2.2%
Pinaceae, 2.0%
Salix, 1.8%
Plantago, 1.8%
Amaranthaceae, 1.2%
Betula, 1.1%
Urticaceae, 0.9%
Liquidambar, 0.9%
Juglans, 0.8%
Carya, 0.7%
Jan
Feb
Mar
Apr
May
Jun
Jul
Aug
Sep
Oct
Nov
Dec
1000
100
10
1
0
NaN

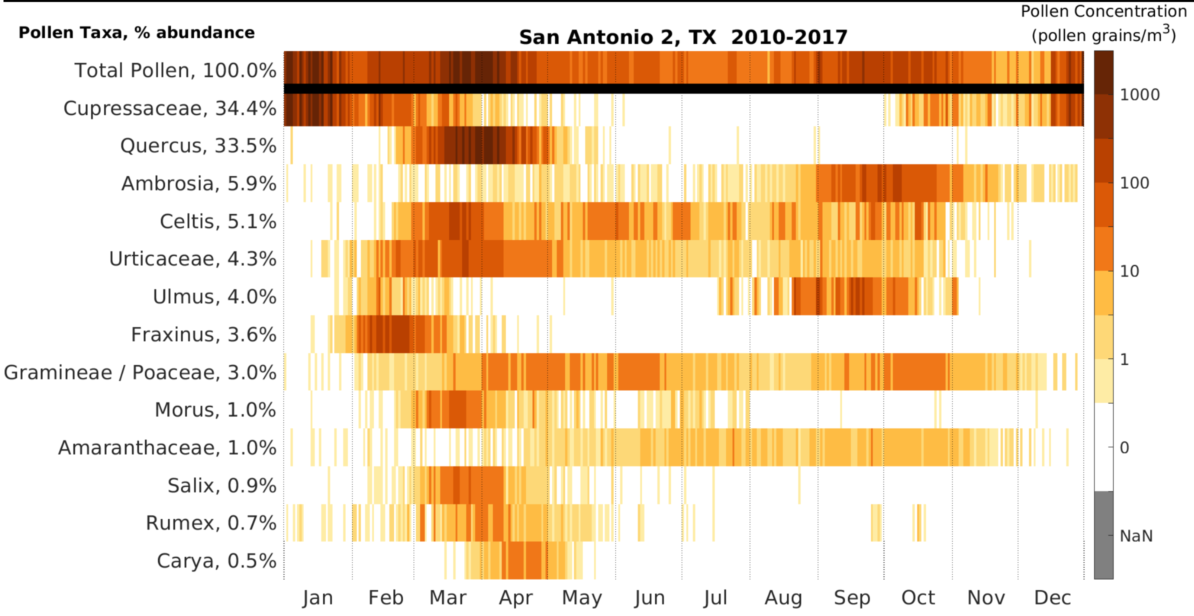
Pollen Taxa, % abundance
San Antonio 2, TX 2010-2017
Pollen Concentration
(pollen grains/m3)
Total Pollen, 100.0%
Cupressaceae, 34.4%
Quercus, 33.5%
Ambrosia, 5.9%
Celtis, 5.1%
Urticaceae, 4.3%
Ulmus, 4.0%
Fraxinus, 3.6%
Gramineae / Poaceae, 3.0%
Morus, 1.0%
Amaranthaceae, 1.0%
Salix, 0.9%
Rumex, 0.7%
Carya, 0.5%
Jan
Feb
Mar
Apr
May
Jun
Jul
Aug
Sep
Oct
Nov
Dec
1000
100
10
1
0
NaN

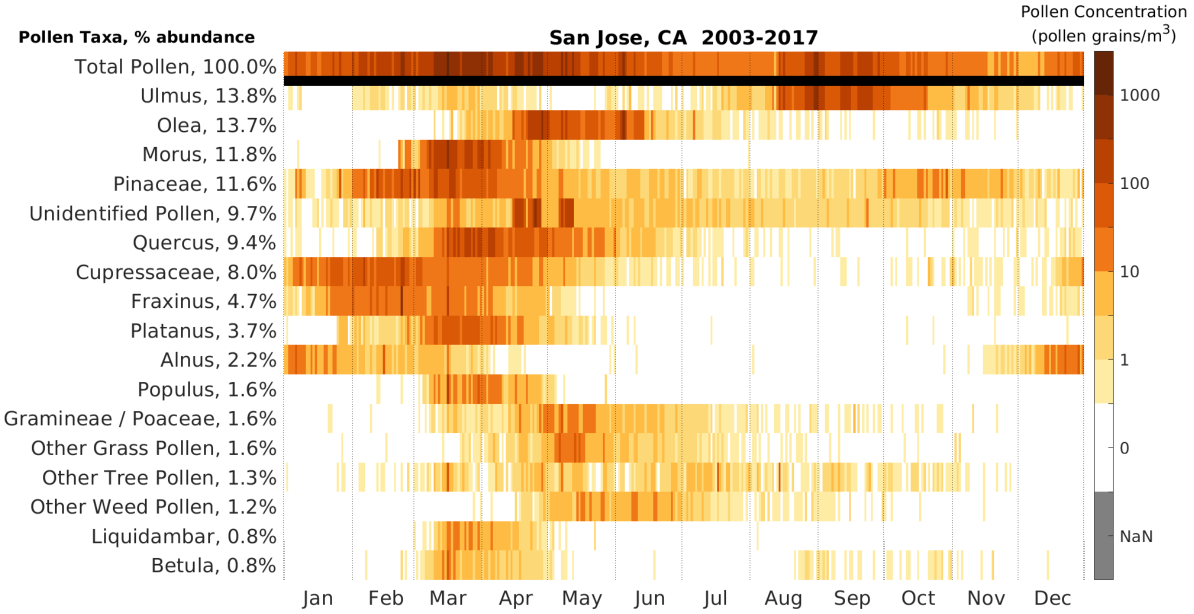
Pollen Taxa, % abundance
San Jose, CA 2003-2017
Pollen Concentration (pollen grains/m3)
Total Pollen, 100.0%
Ulmus, 13.8%
Olea, 13.7%
Morus, 11.8%
Pinaceae, 11.6%
Unidentified Pollen, 9.7%
Quercus, 9.4%
Cupressaceae, 8.0%
Fraxinus, 4.7%
Platanus, 3.7%
Alnus, 2.2%
Populus, 1.6%
Gramineae / Poaceae, 1.6%
Other Grass Pollen, 1.6%
Other Tree Pollen, 1.3%
Other Weed Pollen, 1.2%
Liquidambar, 0.8%
Betula, 0.8%
Jan Feb Mar Apr May Jun Jul Aug Sep Oct Nov Dec
1000
100
10
1
0
NaN

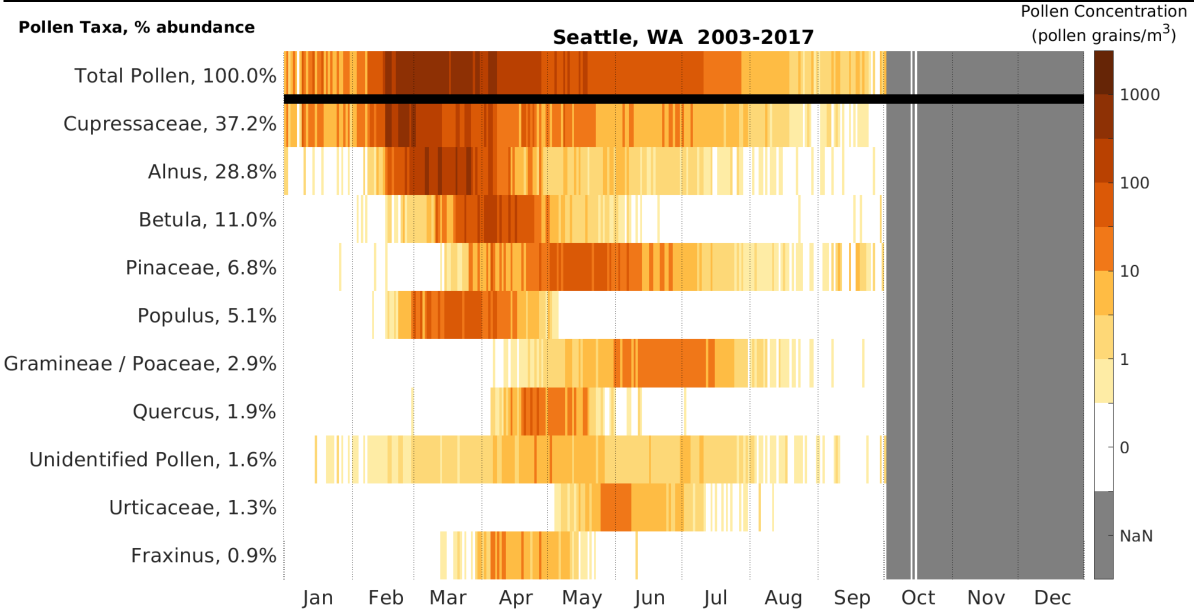
Pollen Taxa, % abundance
Seattle, WA 2003-2017
Pollen Concentration (pollen grains/m3)
Total Pollen, 100.0%
Cupressaceae, 37.2%
Alnus, 28.8%
Betula, 11.0%
Pinaceae, 6.8%
Populus, 5.1%
Gramineae / Poaceae, 2.9%
Quercus, 1.9%
Unidentified Pollen, 1.6%
Urticaceae, 1.3%
Fraxinus, 0.9%
Jan Feb Mar Apr May Jun Jul Aug Sep Oct Nov Dec
1000
100
10
1
0
NaN

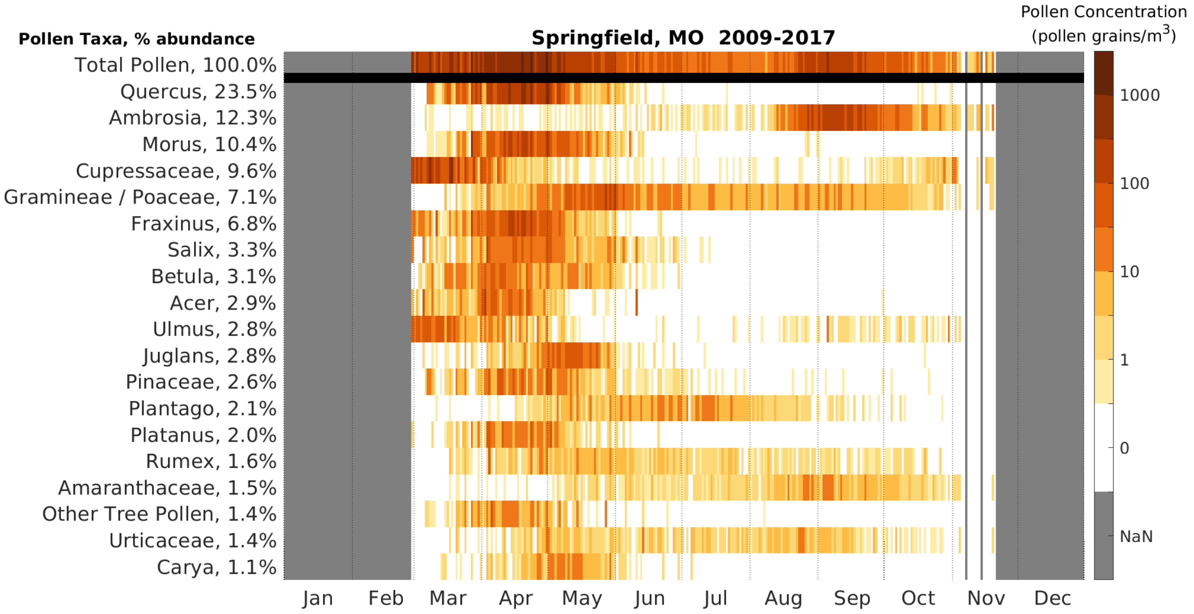
Pollen Taxa, % abundance
Springfield, MO 2009-2017
Pollen Concentration
(pollen grains/m3)
Total Pollen, 100.0%
Quercus, 23.5%
Ambrosia, 12.3%
Morus, 10.4%
Cupressaceae, 9.6%
Gramineae / Poaceae, 7.1%
Fraxinus, 6.8%
Salix, 3.3%
Betula, 3.1%
Acer, 2.9%
Ulmus, 2.8%
Juglans, 2.8%
Pinaceae, 2.6%
Plantago, 2.1%
Platanus, 2.0%
Rumex, 1.6%
Amaranthaceae, 1.5%
Other Tree Pollen, 1.4%
Urticaceae, 1.4%
Carya, 1.1%
Jan
Feb
Mar
Apr
May
Jun
Jul
Aug
Sep
Oct
Nov
Dec
1000
100
10
1
0
NaN

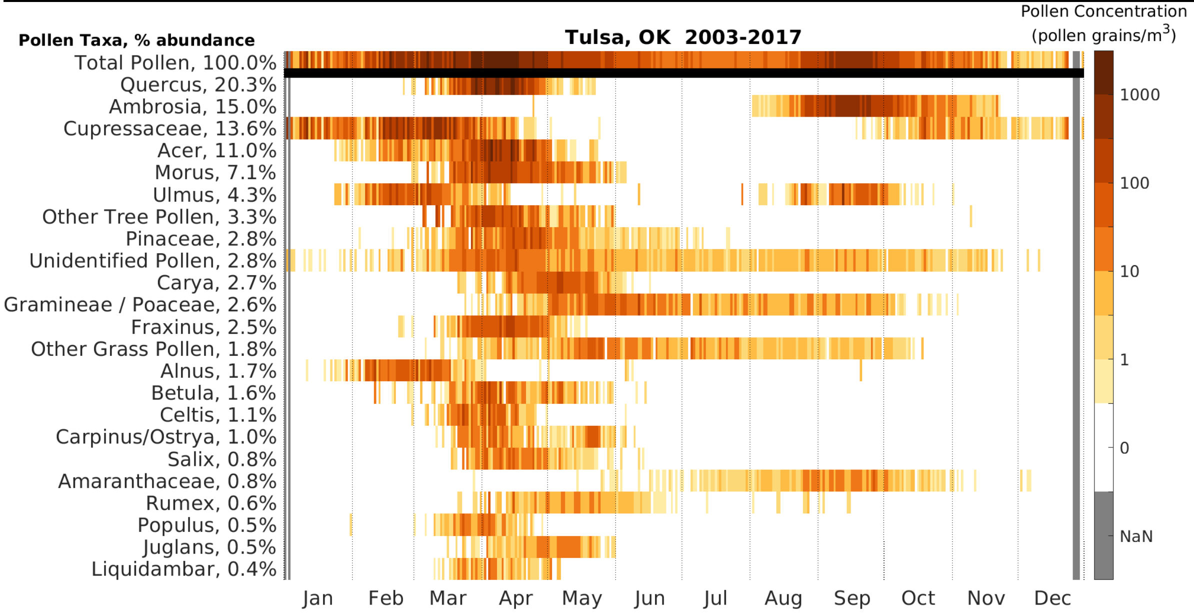
Pollen Taxa, % abundance
Tulsa, OK 2003-2017
Pollen Concentration
(pollen grains/m3)
Total Pollen, 100.0%
Quercus, 20.3%
Ambrosia, 15.0%
Cupressaceae, 13.6%
Acer, 11.0%
Morus, 7.1%
Ulmus, 4.3%
Other Tree Pollen, 3.3%
Pinaceae, 2.8%
Unidentified Pollen, 2.8%
Carya, 2.7%
Gramineae / Poaceae, 2.6%
Fraxinus, 2.5%
Other Grass Pollen, 1.8%
Alnus, 1.7%
Betula, 1.6%
Celtis, 1.1%
Carpinus/Ostrya, 1.0%
Salix, 0.8%
Amaranthaceae, 0.8%
Rumex, 0.6%
Populus, 0.5%
Juglans, 0.5%
Liquidambar, 0.4%
Jan
Feb
Mar
Apr
May
Jun
Jul
Aug
Sep
Oct
Nov
Dec
1000
100
10
1
0
NaN

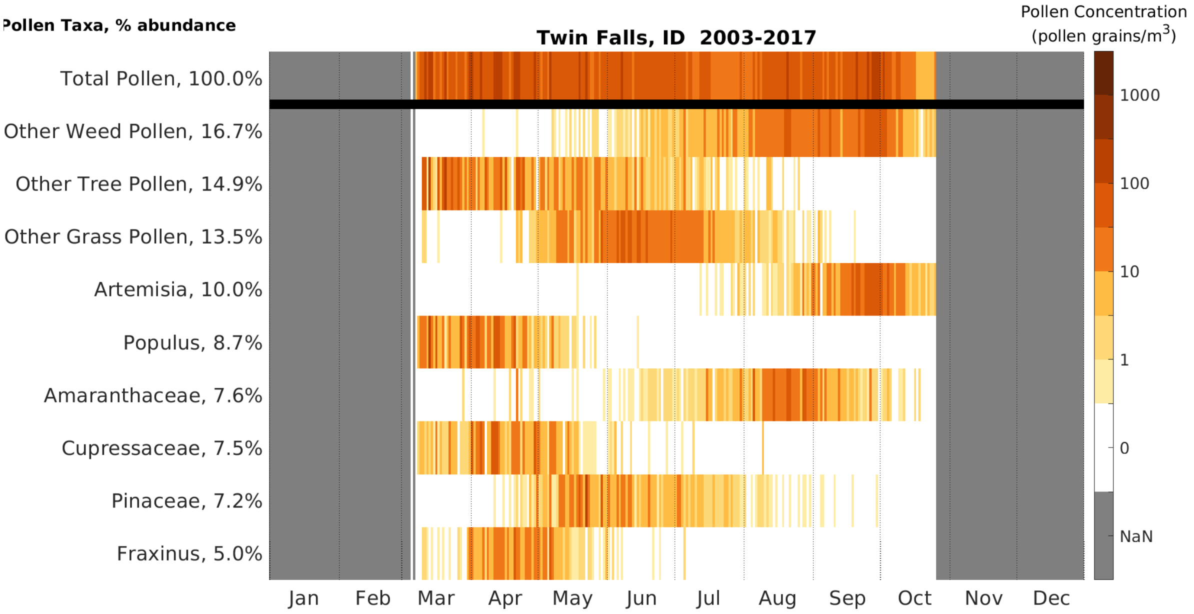
Pollen Taxa, % abundance
Twin Falls, ID 2003-2017
Pollen Concentration (pollen grains/m3)
Total Pollen, 100.0%
Other Weed Pollen, 16.7%
Other Tree Pollen, 14.9%
Other Grass Pollen, 13.5%
Artemisia, 10.0%
Populus, 8.7%
Amaranthaceae, 7.6%
Cupressaceae, 7.5%
Pinaceae, 7.2%
Fraxinus, 5.0%
Jan Feb Mar Apr May Jun Jul Aug Sep Oct Nov Dec
1000
100
10
1
0
NaN

33

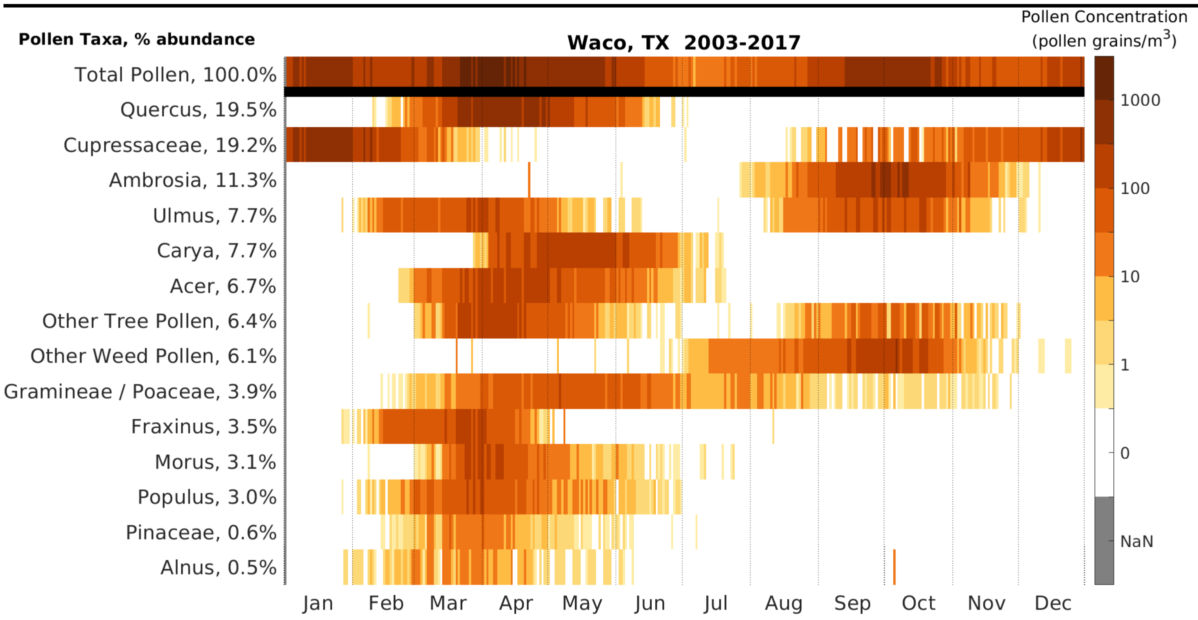
Pollen Taxa, % abundance
Waco, TX 2003-2017
Pollen Concentration (pollen grains/m3)
Total Pollen, 100.0%
Quercus, 19.5%
Cupressaceae, 19.2%
Ambrosia, 11.3%
Ulmus, 7.7%
Carya, 7.7%
Acer, 6.7%
Other Tree Pollen, 6.4%
Other Weed Pollen, 6.1%
Gramineae / Poaceae, 3.9%
Fraxinus, 3.5%
Morus, 3.1%
Populus, 3.0%
Pinaceae, 0.6%
Alnus, 0.5%
Jan Feb Mar Apr May Jun Jul Aug Sep Oct Nov Dec
1000
100
10
1
0
NaN

34

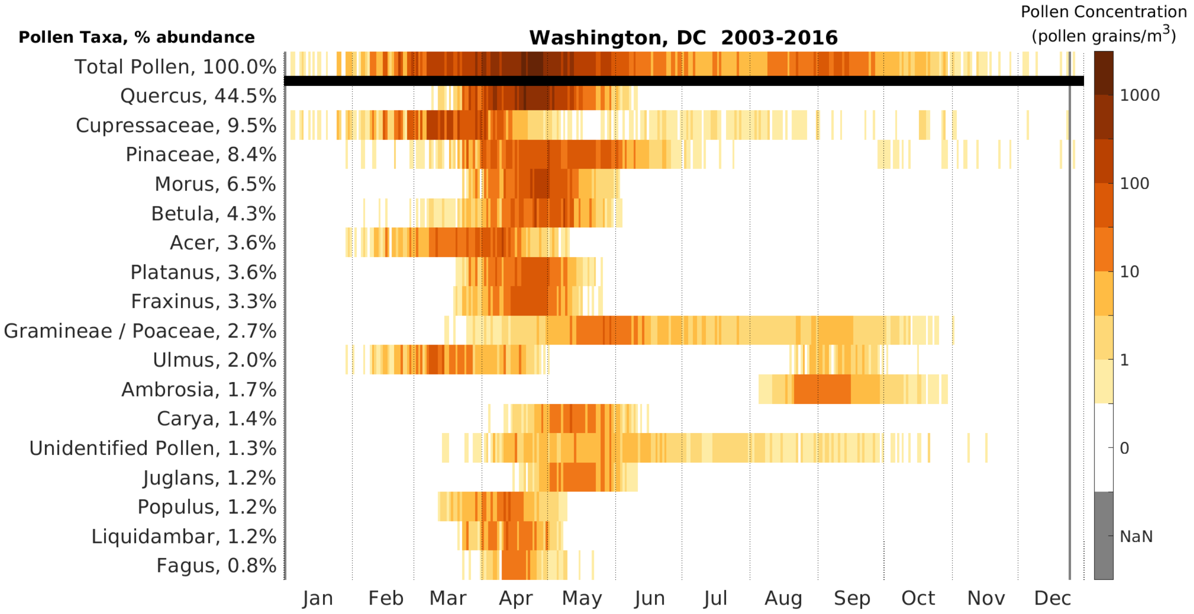

Pollen Taxa, % abundance
Washington, DC 2003-2016
Pollen Concentration (pollen grains/m3)
Total Pollen, 100.0%
Quercus, 44.5%
Cupressaceae, 9.5%
Pinaceae, 8.4%
Morus, 6.5%
Betula, 4.3%
Acer, 3.6%
Platanus, 3.6%
Fraxinus, 3.3%
Gramineae / Poaceae, 2.7%
Ulmus, 2.0%
Ambrosia, 1.7%
Carya, 1.4%
Unidentified Pollen, 1.3%
Juglans, 1.2%
Populus, 1.2%
Liquidambar, 1.2%
Fagus, 0.8%
Jan Feb Mar Apr May Jun Jul Aug Sep Oct Nov Dec
1000
100
10
1
0
NaN

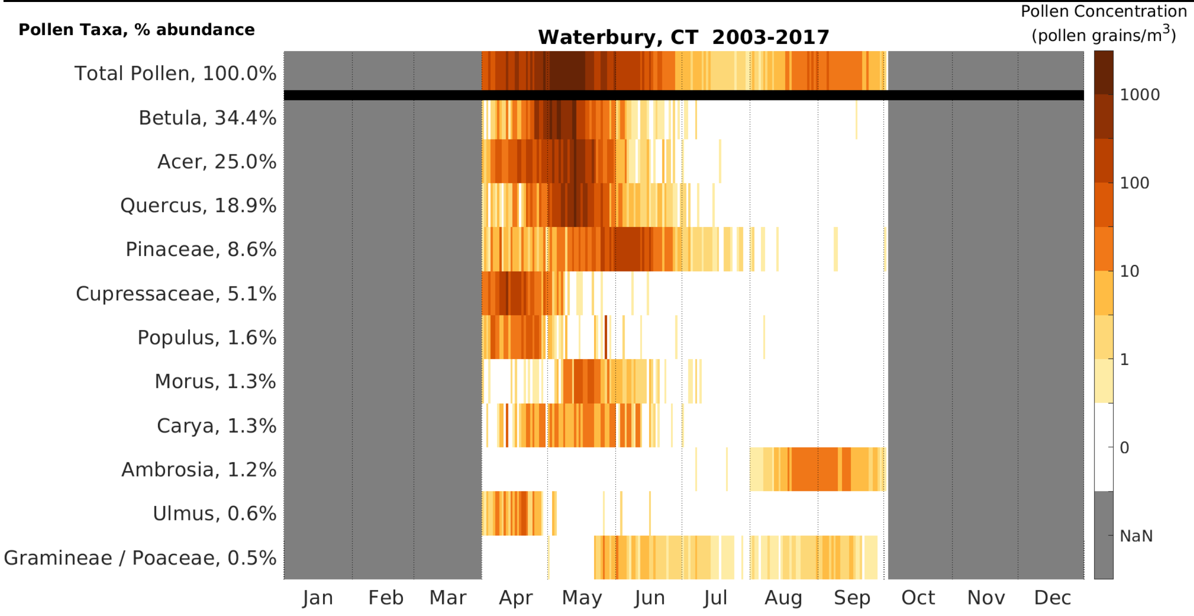

Pollen Taxa, % abundance
Waterbury, CT 2003-2017
Pollen Concentration (pollen grains/m3)
Total Pollen, 100.0%
Betula, 34.4%
Acer, 25.0%
Quercus, 18.9%
Pinaceae, 8.6%
Cupressaceae, 5.1%
Populus, 1.6%
Morus, 1.3%
Carya, 1.3%
Ambrosia, 1.2%
Ulmus, 0.6%
Gramineae / Poaceae, 0.5%
Jan Feb Mar Apr May Jun Jul Aug Sep Oct Nov Dec
1000
100
10
1
0
NaN

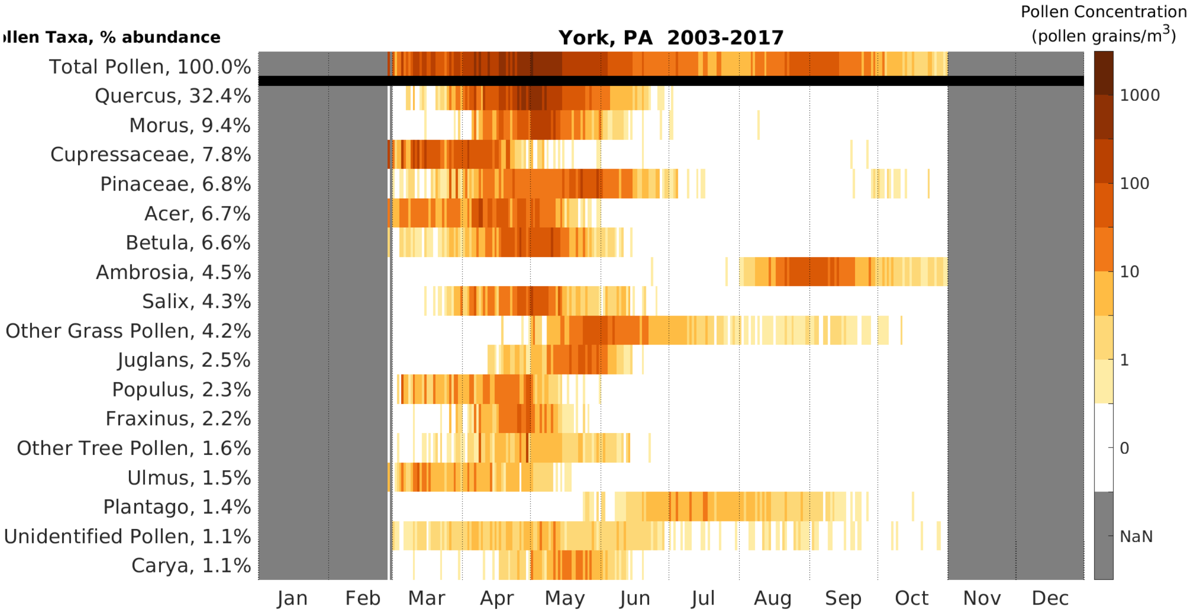
llen Taxa, % abundance
York, PA 2003-2017
Pollen Concentration
(pollen grains/m3)
Total Pollen, 100.0%
Quercus, 32.4%
Morus, 9.4%
Cupressaceae, 7.8%
Pinaceae, 6.8%
Acer, 6.7%
Betula, 6.6%
Ambrosia, 4.5%
Salix, 4.3%
Other Grass Pollen, 4.2%
Juglans, 2.5%
Populus, 2.3%
Fraxinus, 2.2%
Other Tree Pollen, 1.6%
Ulmus, 1.5%
Plantago, 1.4%
Unidentified Pollen, 1.1%
Carya, 1.1%
Jan
Feb
Mar
Apr
May
Jun
Jul
Aug
Sep
Oct
Nov
Dec
1000
100
10
1
0
NaN

Taxon-specific pollen calendars

Pollen calendars for pollen taxon listed in title. Daily mean climatology of pollen concentration, 2003-2017. NAB stations are ordered by latitude. Missing data are shaded grey and denoted NaN in the colorbar.

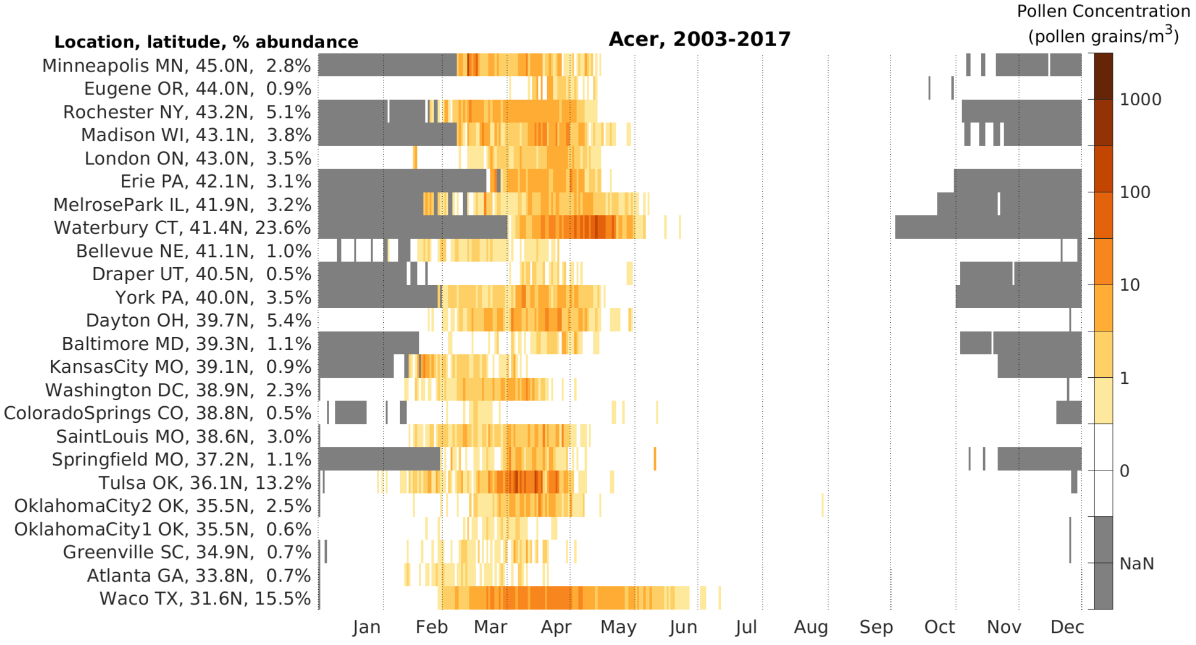

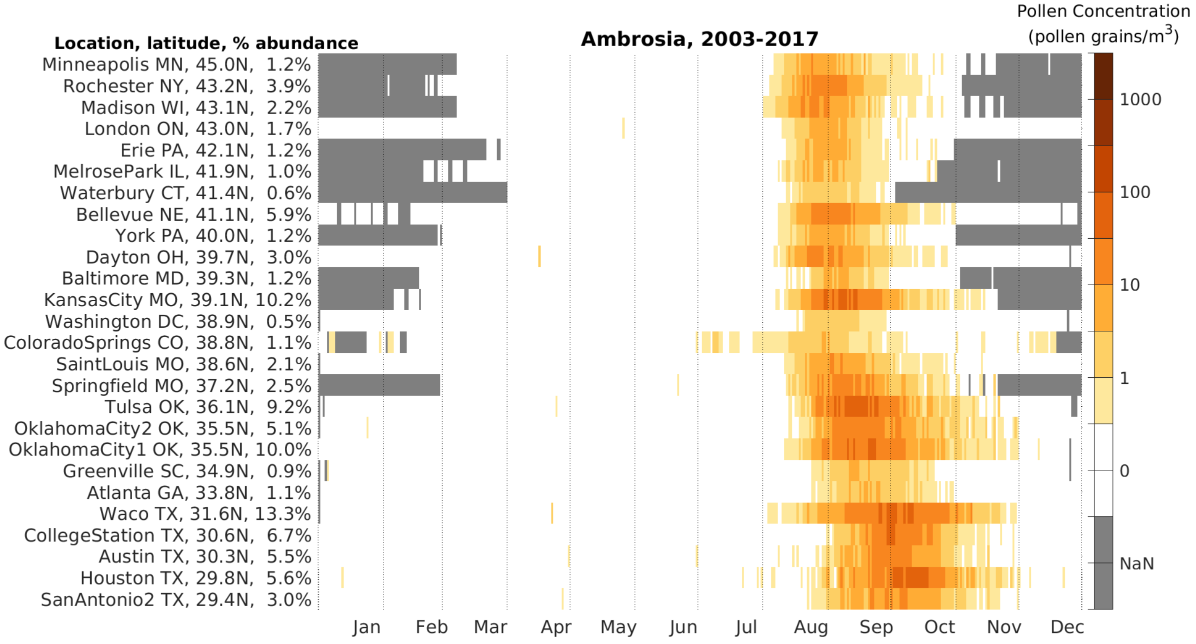

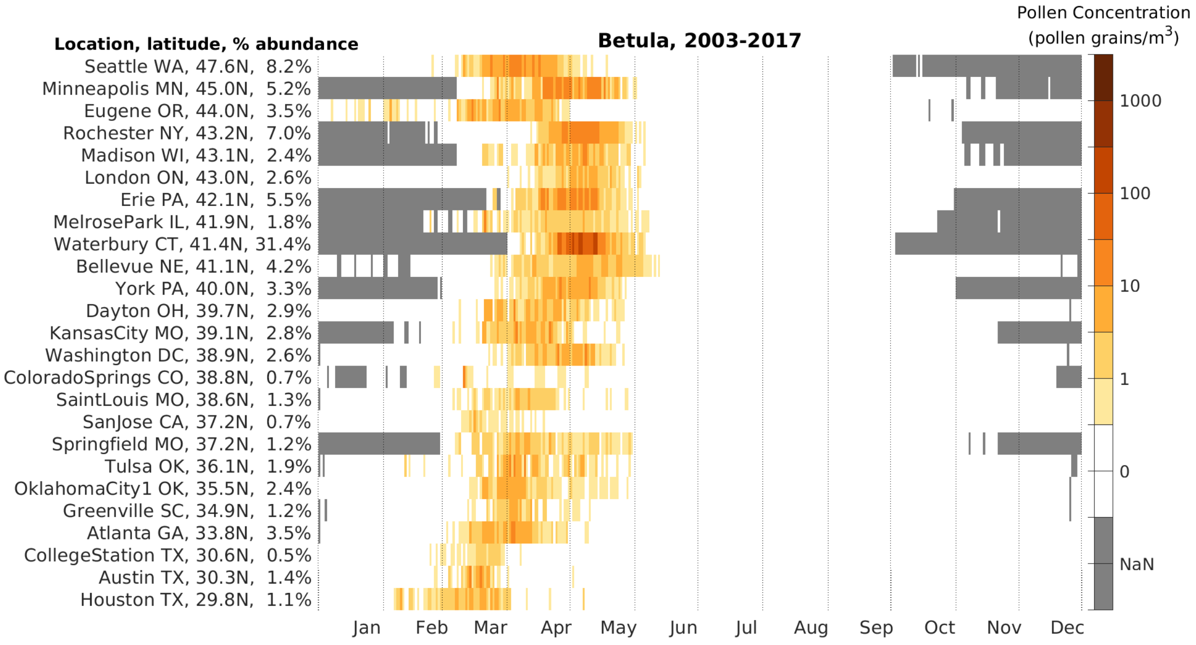
Location, latitude, % abundance
Betula, 2003-2017
Pollen Concentration
(pollen grains/m3)
Seattle WA, 47.6N, 8.2%
Minneapolis MN, 45.0N, 5.2%
Eugene OR, 44.0N, 3.5%
Rochester NY, 43.2N, 7.0%
Madison WI, 43.1N, 2.4%
London ON, 43.0N, 2.6%
Erie PA, 42.1N, 5.5%
MelrosePark IL, 41.9N, 1.8%
Waterbury CT, 41.4N, 31.4%
Bellevue NE, 41.1N, 4.2%
York PA, 40.0N, 3.3%
Dayton OH, 39.7N, 2.9%
KansasCity MO, 39.1N, 2.8%
Washington DC, 38.9N, 2.6%
ColoradoSprings CO, 38.8N, 0.7%
SaintLouis MO, 38.6N, 1.3%
SanJose CA, 37.2N, 0.7%
Springfield MO, 37.2N, 1.2%
Tulsa OK, 36.1N, 1.9%
OklahomaCity1 OK, 35.5N, 2.4%
Greenville SC, 34.9N, 1.2%
Atlanta GA, 33.8N, 3.5%
CollegeStation TX, 30.6N, 0.5%
Austin TX, 30.3N, 1.4%
Houston TX, 29.8N, 1.1%
Jan Feb Mar Apr May Jun Jul Aug Sep Oct Nov Dec
1000
100
10
1
0
NaN

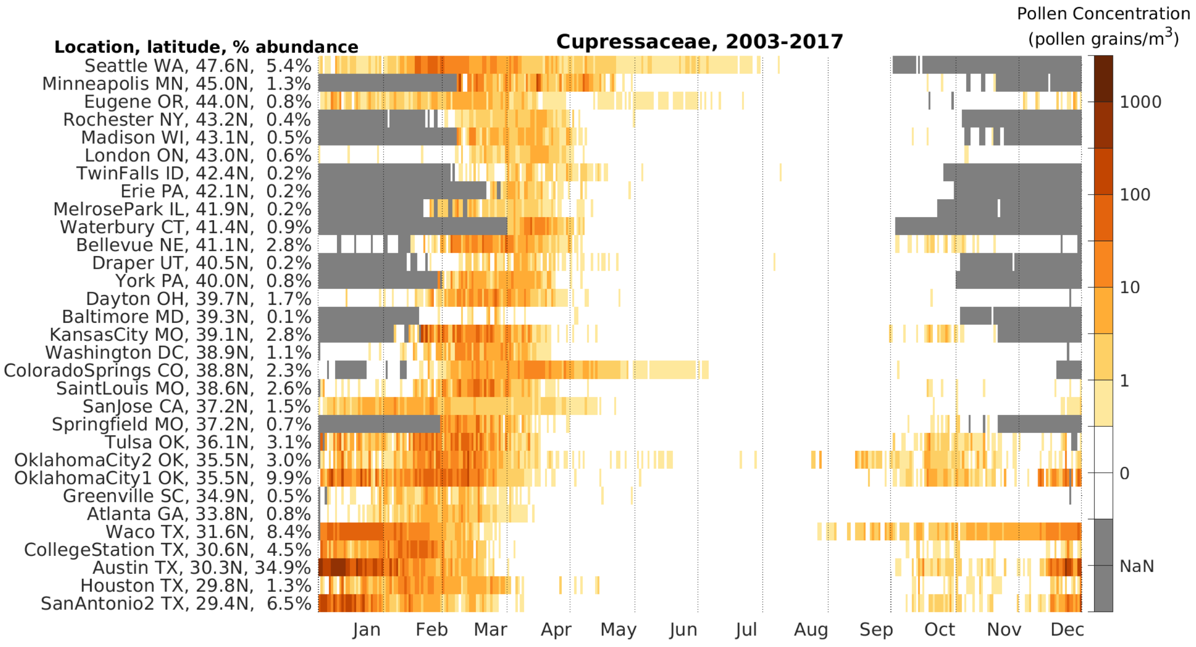
Location, latitude, % abundance
Cupressaceae, 2003-2017
Pollen Concentration
(pollen grains/m3)
Seattle WA, 47.6N, 5.4%
Minneapolis MN, 45.0N, 1.3%
Eugene OR, 44.0N, 0.8%
Rochester NY, 43.2N, 0.4%
Madison WI, 43.1N, 0.5%
London ON, 43.0N, 0.6%
TwinFalls ID, 42.4N, 0.2%
Erie PA, 42.1N, 0.2%
MelrosePark IL, 41.9N, 0.2%
Waterbury CT, 41.4N, 0.9%
Bellevue NE, 41.1N, 2.8%
Draper UT, 40.5N, 0.2%
York PA, 40.0N, 0.8%
Dayton OH, 39.7N, 1.7%
Baltimore MD, 39.3N, 0.1%
KansasCity MO, 39.1N, 2.8%
Washington DC, 38.9N, 1.1%
ColoradoSprings CO, 38.8N, 2.3%
SaintLouis MO, 38.6N, 2.6%
SanJose CA, 37.2N, 1.5%
Springfield MO, 37.2N, 0.7%
Tulsa OK, 36.1N, 3.1%
OklahomaCity2 OK, 35.5N, 3.0%
OklahomaCity1 OK, 35.5N, 9.9%
Greenville SC, 34.9N, 0.5%
Atlanta GA, 33.8N, 0.8%
Waco TX, 31.6N, 8.4%
CollegeStation TX, 30.6N, 4.5%
Austin TX, 30.3N, 34.9%
Houston TX, 29.8N, 1.3%
SanAntonio2 TX, 29.4N, 6.5%
Jan Feb Mar Apr May Jun Jul Aug Sep Oct Nov Dec
1000
100
10
1
0
NaN

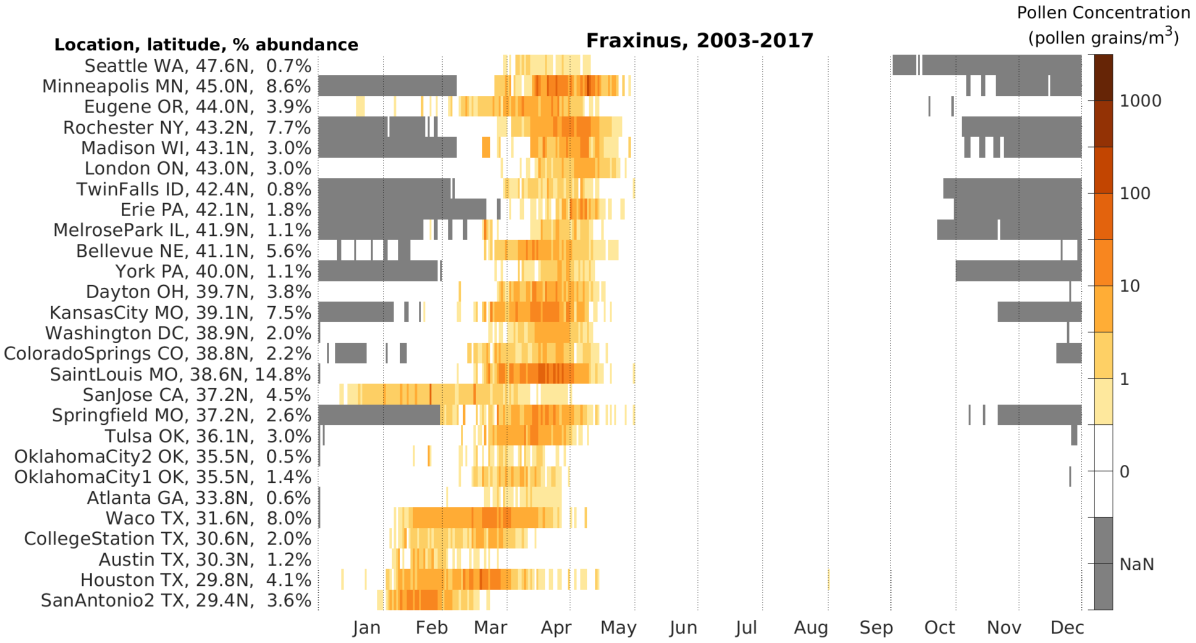
Fraxinus, 2003-2017
Location, latitude, % abundance
Seattle WA, 47.6N, 0.7%
Minneapolis MN, 45.0N, 8.6%
Eugene OR, 44.0N, 3.9%
Rochester NY, 43.2N, 7.7%
Madison WI, 43.1N, 3.0%
London ON, 43.0N, 3.0%
TwinFalls ID, 42.4N, 0.8%
Erie PA, 42.1N, 1.8%
MelrosePark IL, 41.9N, 1.1%
Bellevue NE, 41.1N, 5.6%
York PA, 40.0N, 1.1%
Dayton OH, 39.7N, 3.8%
KansasCity MO, 39.1N, 7.5%
Washington DC, 38.9N, 2.0%
ColoradoSprings CO, 38.8N, 2.2%
SaintLouis MO, 38.6N, 14.8%
SanJose CA, 37.2N, 4.5%
Springfield MO, 37.2N, 2.6%
Tulsa OK, 36.1N, 3.0%
OklahomaCity2 OK, 35.5N, 0.5%
OklahomaCity1 OK, 35.5N, 1.4%
Atlanta GA, 33.8N, 0.6%
Waco TX, 31.6N, 8.0%
CollegeStation TX, 30.6N, 2.0%
Austin TX, 30.3N, 1.2%
Houston TX, 29.8N, 4.1%
SanAntonio2 TX, 29.4N, 3.6%
Jan Feb Mar Apr May Jun Jul Aug Sep Oct Nov Dec
Pollen Concentration
(pollen grains/m3)
1000
100
10
1
0
NaN

52

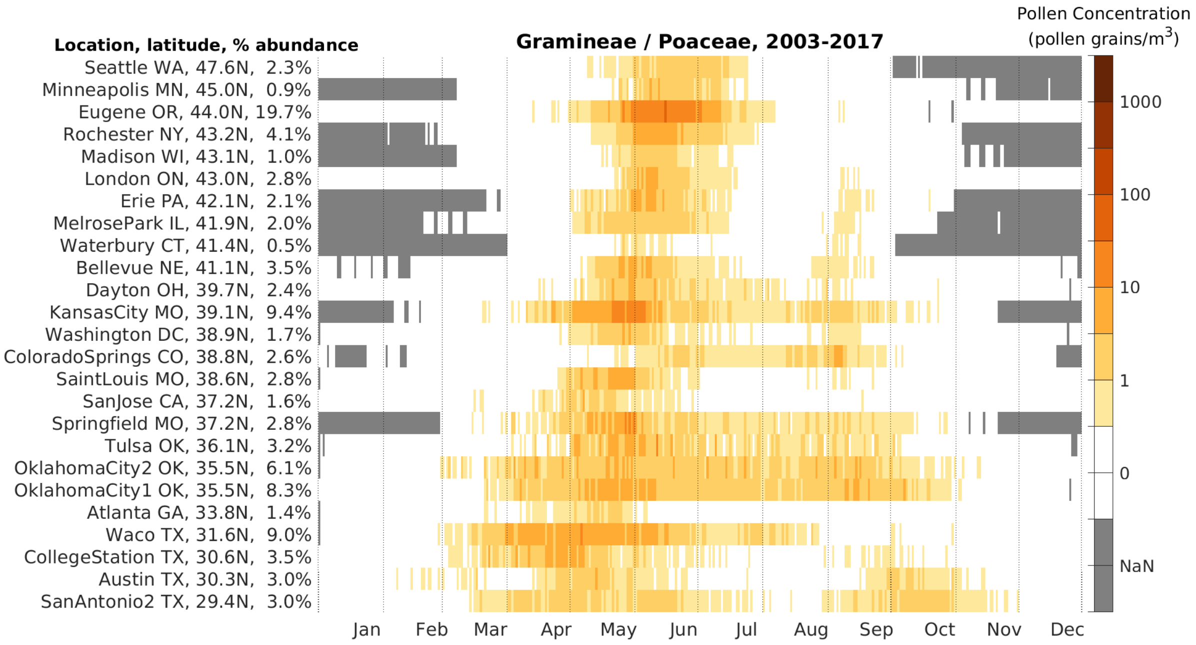
Gramineae / Poaceae, 2003-2017
Location, latitude, % abundance
Seattle WA, 47.6N, 2.3%
Minneapolis MN, 45.0N, 0.9%
Eugene OR, 44.0N, 19.7%
Rochester NY, 43.2N, 4.1%
Madison WI, 43.1N, 1.0%
London ON, 43.0N, 2.8%
Erie PA, 42.1N, 2.1%
MelrosePark IL, 41.9N, 2.0%
Waterbury CT, 41.4N, 0.5%
Bellevue NE, 41.1N, 3.5%
Dayton OH, 39.7N, 2.4%
KansasCity MO, 39.1N, 9.4%
Washington DC, 38.9N, 1.7%
ColoradoSprings CO, 38.8N, 2.6%
SaintLouis MO, 38.6N, 2.8%
SanJose CA, 37.2N, 1.6%
Springfield MO, 37.2N, 2.8%
Tulsa OK, 36.1N, 3.2%
OklahomaCity2 OK, 35.5N, 6.1%
OklahomaCity1 OK, 35.5N, 8.3%
Atlanta GA, 33.8N, 1.4%
Waco TX, 31.6N, 9.0%
CollegeStation TX, 30.6N, 3.5%
Austin TX, 30.3N, 3.0%
SanAntonio2 TX, 29.4N, 3.0%
Jan Feb Mar Apr May Jun Jul Aug Sep Oct Nov Dec
Pollen Concentration
(pollen grains/m3)
1000
100
10
1
0
NaN

53

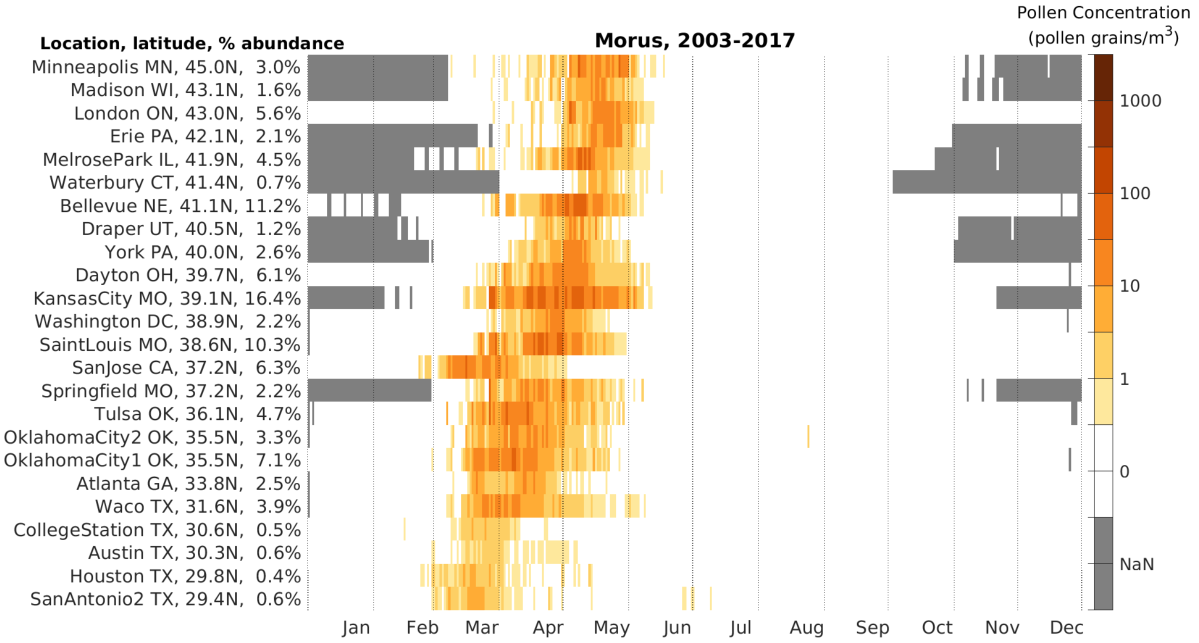
Pollen Concentration
(pollen grains/m3)
Location, latitude, % abundance
Morus, 2003-2017
Minneapolis MN, 45.0N, 3.0%
Madison WI, 43.1N, 1.6%
London ON, 43.0N, 5.6%
Erie PA, 42.1N, 2.1%
MelrosePark IL, 41.9N, 4.5%
Waterbury CT, 41.4N, 0.7%
Bellevue NE, 41.1N, 11.2%
Draper UT, 40.5N, 1.2%
York PA, 40.0N, 2.6%
Dayton OH, 39.7N, 6.1%
KansasCity MO, 39.1N, 16.4%
Washington DC, 38.9N, 2.2%
SaintLouis MO, 38.6N, 10.3%
SanJose CA, 37.2N, 6.3%
Springfield MO, 37.2N, 2.2%
Tulsa OK, 36.1N, 4.7%
OklahomaCity2 OK, 35.5N, 3.3%
OklahomaCity1 OK, 35.5N, 7.1%
Atlanta GA, 33.8N, 2.5%
Waco TX, 31.6N, 3.9%
CollegeStation TX, 30.6N, 0.5%
Austin TX, 30.3N, 0.6%
Houston TX, 29.8N, 0.4%
SanAntonio2 TX, 29.4N, 0.6%
Jan Feb Mar Apr May Jun Jul Aug Sep Oct Nov Dec
1000
100
10
1
0
NaN

54

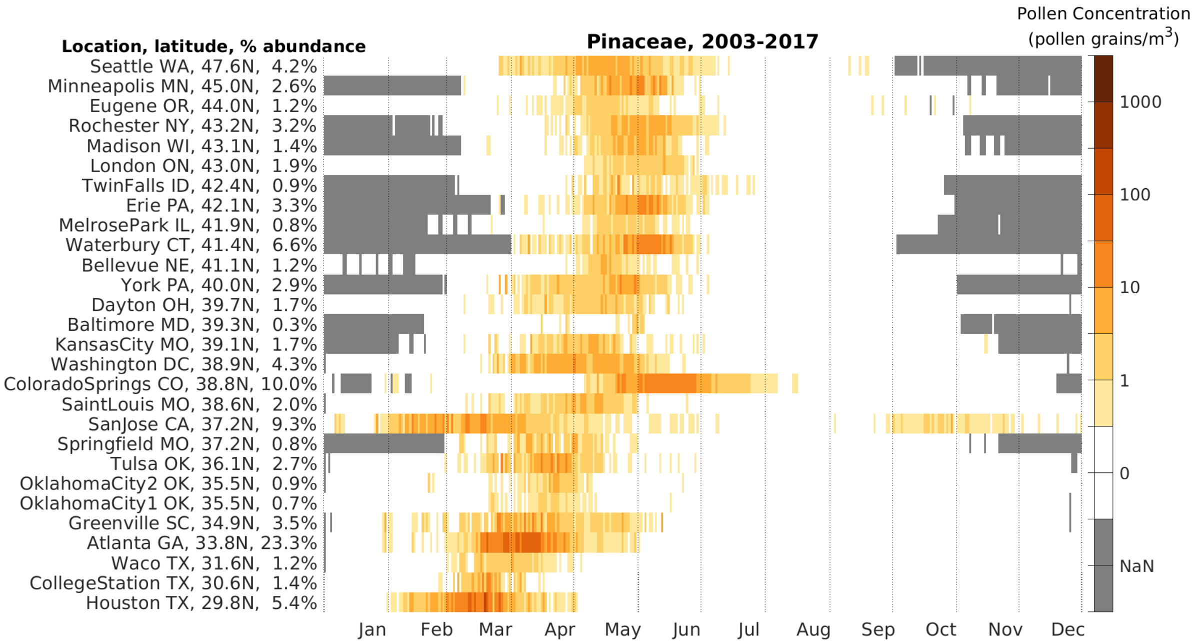
Pollen Concentration
(pollen grains/m3)
Location, latitude, % abundance
Pinaceae, 2003-2017
Seattle WA, 47.6N, 4.2%
Minneapolis MN, 45.0N, 2.6%
Eugene OR, 44.0N, 1.2%
Rochester NY, 43.2N, 3.2%
Madison WI, 43.1N, 1.4%
London ON, 43.0N, 1.9%
TwinFalls ID, 42.4N, 0.9%
Erie PA, 42.1N, 3.3%
MelrosePark IL, 41.9N, 0.8%
Waterbury CT, 41.4N, 6.6%
Bellevue NE, 41.1N, 1.2%
York PA, 40.0N, 2.9%
Dayton OH, 39.7N, 1.7%
Baltimore MD, 39.3N, 0.3%
KansasCity MO, 39.1N, 1.7%
Washington DC, 38.9N, 4.3%
ColoradoSprings CO, 38.8N, 10.0%
SaintLouis MO, 38.6N, 2.0%
SanJose CA, 37.2N, 9.3%
Springfield MO, 37.2N, 0.8%
Tulsa OK, 36.1N, 2.7%
OklahomaCity2 OK, 35.5N, 0.9%
OklahomaCity1 OK, 35.5N, 0.7%
Greenville SC, 34.9N, 3.5%
Atlanta GA, 33.8N, 23.3%
Waco TX, 31.6N, 1.2%
CollegeStation TX, 30.6N, 1.4%
Houston TX, 29.8N, 5.4%
Jan Feb Mar Apr May Jun Jul Aug Sep Oct Nov Dec
1000
100
10
1
0
NaN

55

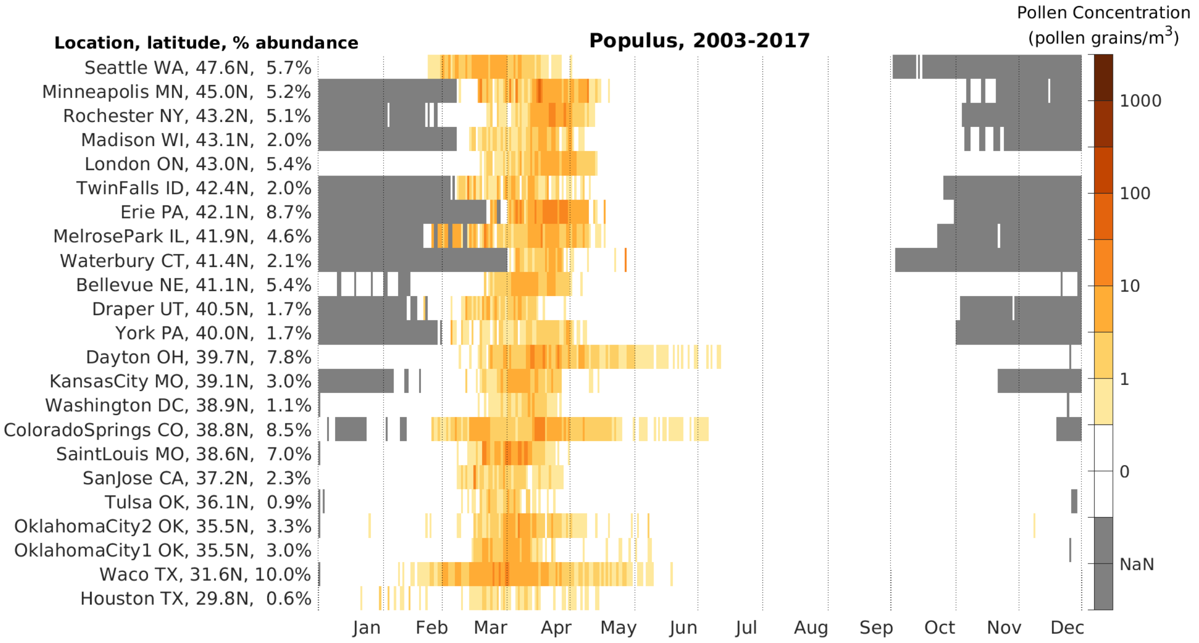
Location, latitude, % abundance
Populus, 2003-2017
Pollen Concentration
(pollen grains/m3)
Seattle WA, 47.6N, 5.7%
Minneapolis MN, 45.0N, 5.2%
Rochester NY, 43.2N, 5.1%
Madison WI, 43.1N, 2.0%
London ON, 43.0N, 5.4%
TwinFalls ID, 42.4N, 2.0%
Erie PA, 42.1N, 8.7%
MelrosePark IL, 41.9N, 4.6%
Waterbury CT, 41.4N, 2.1%
Bellevue NE, 41.1N, 5.4%
Draper UT, 40.5N, 1.7%
York PA, 40.0N, 1.7%
Dayton OH, 39.7N, 7.8%
KansasCity MO, 39.1N, 3.0%
Washington DC, 38.9N, 1.1%
ColoradoSprings CO, 38.8N, 8.5%
SaintLouis MO, 38.6N, 7.0%
SanJose CA, 37.2N, 2.3%
Tulsa OK, 36.1N, 0.9%
OklahomaCity2 OK, 35.5N, 3.3%
OklahomaCity1 OK, 35.5N, 3.0%
Waco TX, 31.6N, 10.0%
Houston TX, 29.8N, 0.6%
Jan Feb Mar Apr May Jun Jul Aug Sep Oct Nov Dec
1000
100
10
1
0
NaN

56

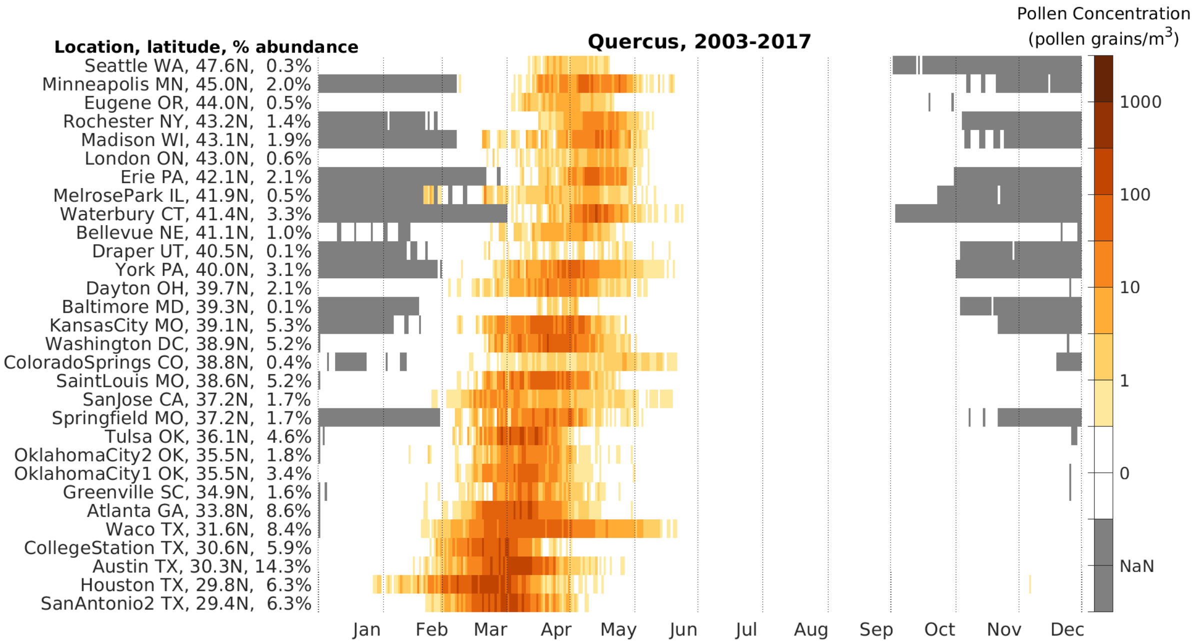
Location, latitude, % abundance
Quercus, 2003-2017
Pollen Concentration
(pollen grains/m3)
Seattle WA, 47.6N, 0.3%
Minneapolis MN, 45.0N, 2.0%
Eugene OR, 44.0N, 0.5%
Rochester NY, 43.2N, 1.4%
Madison WI, 43.1N, 1.9%
London ON, 43.0N, 0.6%
Erie PA, 42.1N, 2.1%
MelrosePark IL, 41.9N, 0.5%
Waterbury CT, 41.4N, 3.3%
Bellevue NE, 41.1N, 1.0%
Draper UT, 40.5N, 0.1%
York PA, 40.0N, 3.1%
Dayton OH, 39.7N, 2.1%
Baltimore MD, 39.3N, 0.1%
KansasCity MO, 39.1N, 5.3%
Washington DC, 38.9N, 5.2%
ColoradoSprings CO, 38.8N, 0.4%
SaintLouis MO, 38.6N, 5.2%
SanJose CA, 37.2N, 1.7%
Springfield MO, 37.2N, 1.7%
Tulsa OK, 36.1N, 4.6%
OklahomaCity2 OK, 35.5N, 1.8%
OklahomaCity1 OK, 35.5N, 3.4%
Greenville SC, 34.9N, 1.6%
Atlanta GA, 33.8N, 8.6%
Waco TX, 31.6N, 8.4%
CollegeStation TX, 30.6N, 5.9%
Austin TX, 30.3N, 14.3%
Houston TX, 29.8N, 6.3%
SanAntonio2 TX, 29.4N, 6.3%
Jan Feb Mar Apr May Jun Jul Aug Sep Oct Nov Dec
1000
100
10
1
0
NaN

57

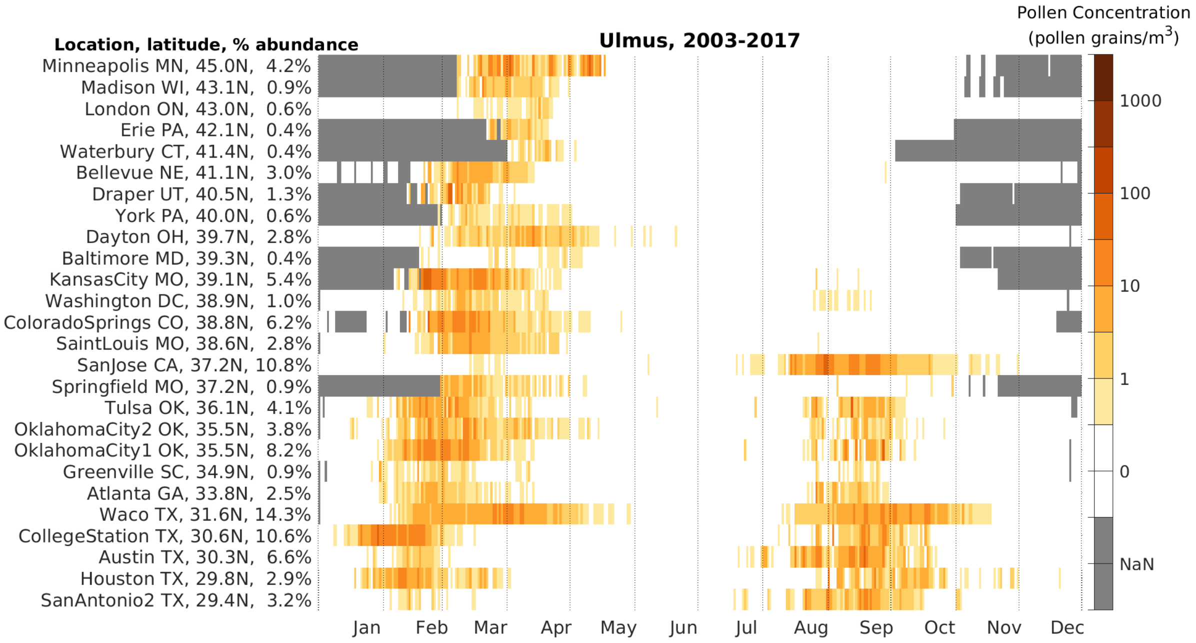
Location, latitude, % abundance
Ulmus, 2003-2017
Pollen Concentration
(pollen grains/m3)
Minneapolis MN, 45.0N, 4.2%
Madison WI, 43.1N, 0.9%
London ON, 43.0N, 0.6%
Erie PA, 42.1N, 0.4%
Waterbury CT, 41.4N, 0.4%
Bellevue NE, 41.1N, 3.0%
Draper UT, 40.5N, 1.3%
York PA, 40.0N, 0.6%
Dayton OH, 39.7N, 2.8%
Baltimore MD, 39.3N, 0.4%
KansasCity MO, 39.1N, 5.4%
Washington DC, 38.9N, 1.0%
ColoradoSprings CO, 38.8N, 6.2%
SaintLouis MO, 38.6N, 2.8%
SanJose CA, 37.2N, 10.8%
Springfield MO, 37.2N, 0.9%
Tulsa OK, 36.1N, 4.1%
OklahomaCity2 OK, 35.5N, 3.8%
OklahomaCity1 OK, 35.5N, 8.2%
Greenville SC, 34.9N, 0.9%
Atlanta GA, 33.8N, 2.5%
Waco TX, 31.6N, 14.3%
CollegeStation TX, 30.6N, 10.6%
Austin TX, 30.3N, 6.6%
Houston TX, 29.8N, 2.9%
SanAntonio2 TX, 29.4N, 3.2%
Jan
Feb
Mar
Apr
May
Jun
Jul
Aug
Sep
Oct
Nov
Dec
1000
100
10
1
0
NaN

Radar Charts

Radar charts for the station at the city and state or province listed in title of figure. Percent total API for the station for the 11 allergenic pollen taxa and the combined category of "Other" which is sum of all the other sampled allergenic pollen taxa.

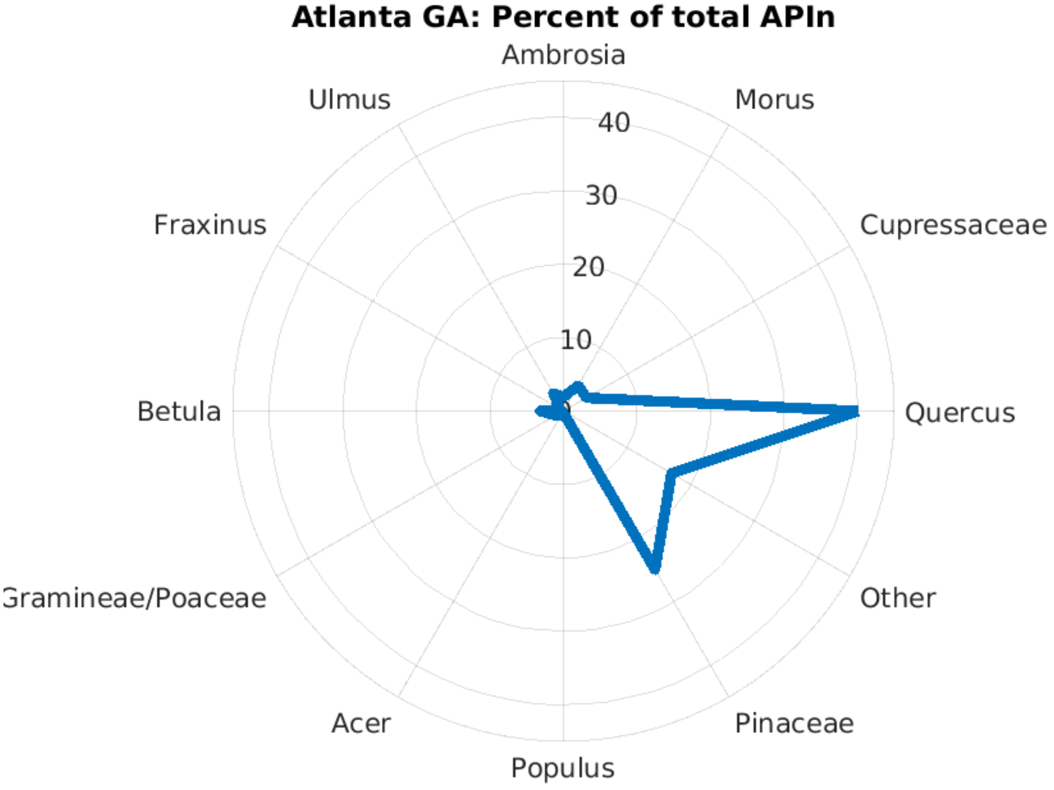

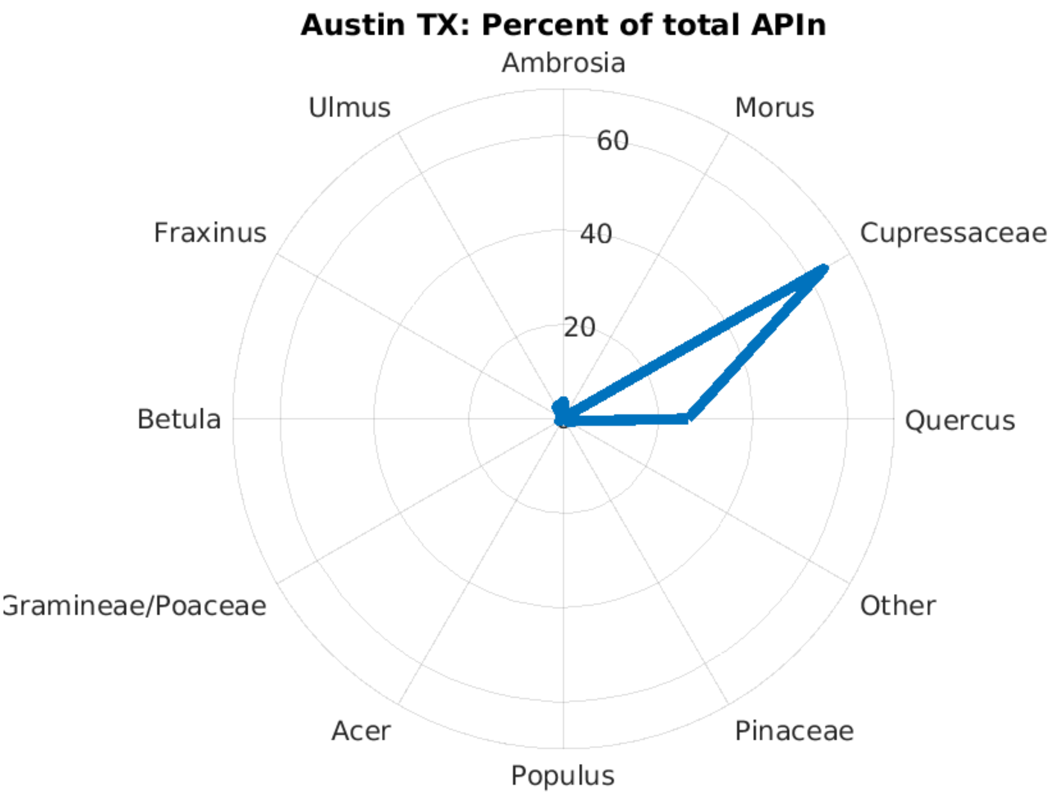
Austin TX: Percent of total APIn
Ambrosia
Morus
Cupressaceae
Quercus
Other
Pinaceae
Populus
Acer
Gramineae/Poaceae
Betula
Fraxinus
Ulmus
20
40
60

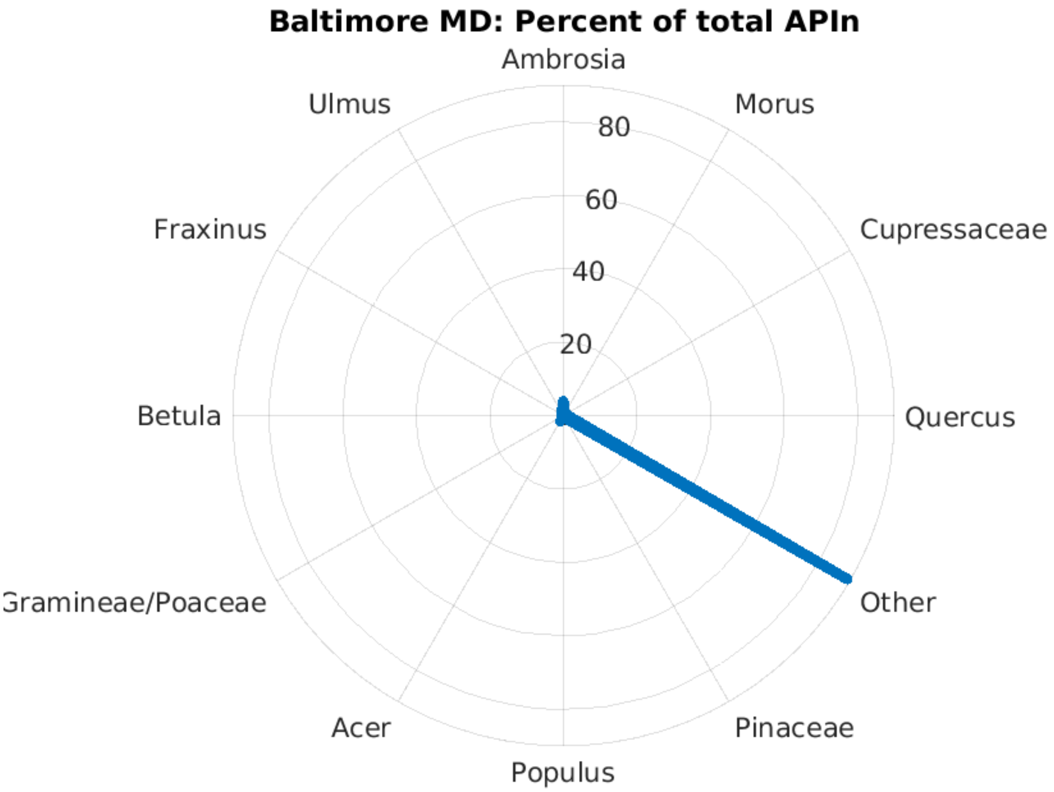
Baltimore MD: Percent of total APIn
Ambrosia
Morus
Cupressaceae
Quercus
Other
Pinaceae
Populus
Acer
Gramineae/Poaceae
Betula
Fraxinus
Ulmus
20
40
60
80

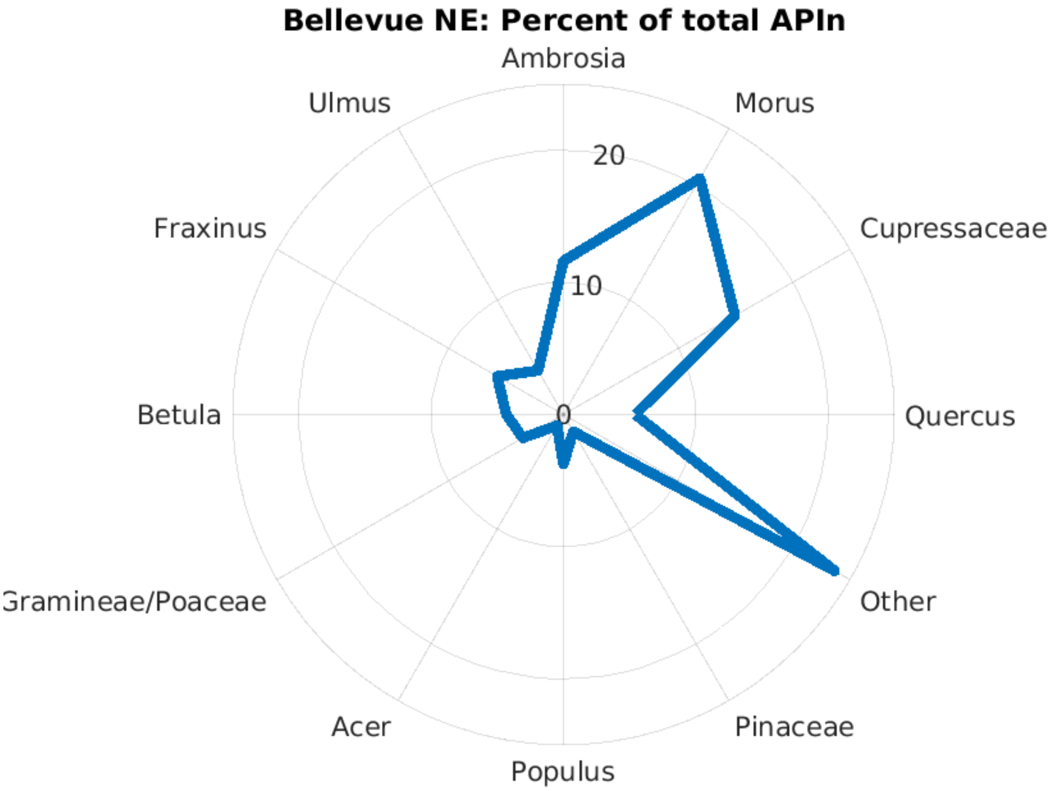
Bellevue NE: Percent of total APIn
Ambrosia
Morus
Cupressaceae
Quercus
Other
Pinaceae
Populus
Acer
Gramineae/Poaceae
Betula
Fraxinus
Ulmus
20
10
0

93

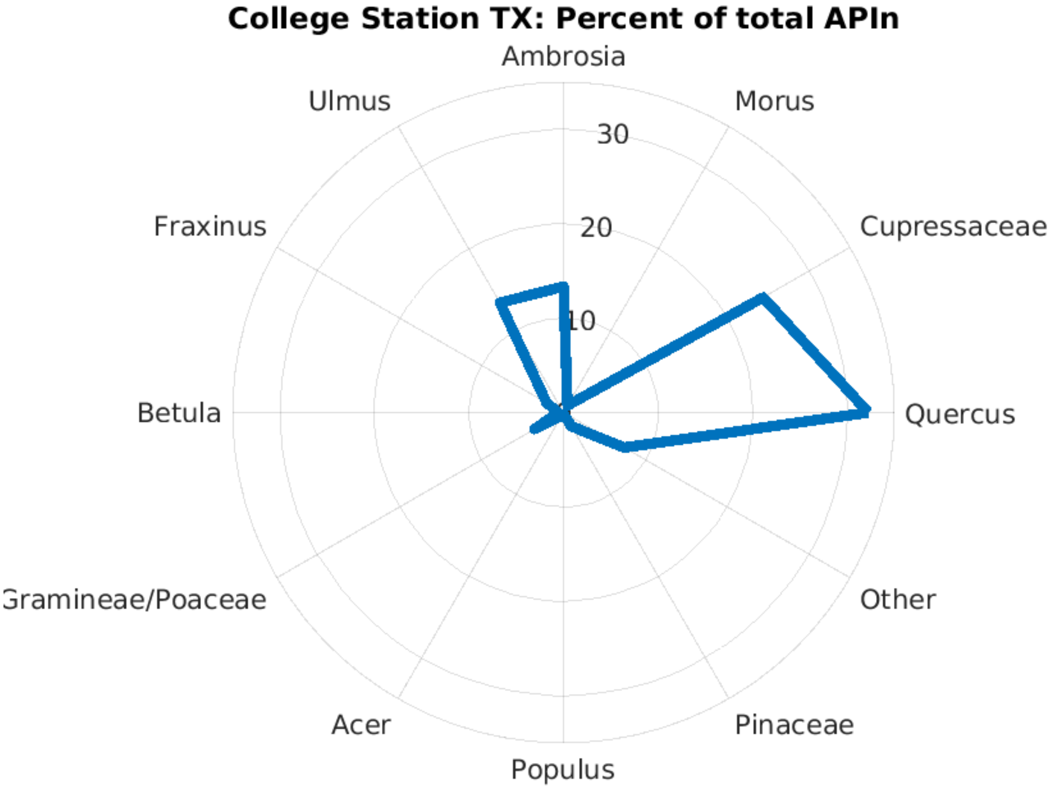
College Station TX: Percent of total APIn
Ambrosia
Morus
Cupressaceae
Quercus
Other
Pinaceae
Populus
Acer
Gramineae/Poaceae
Betula
Fraxinus
Ulmus
30
20
10

94

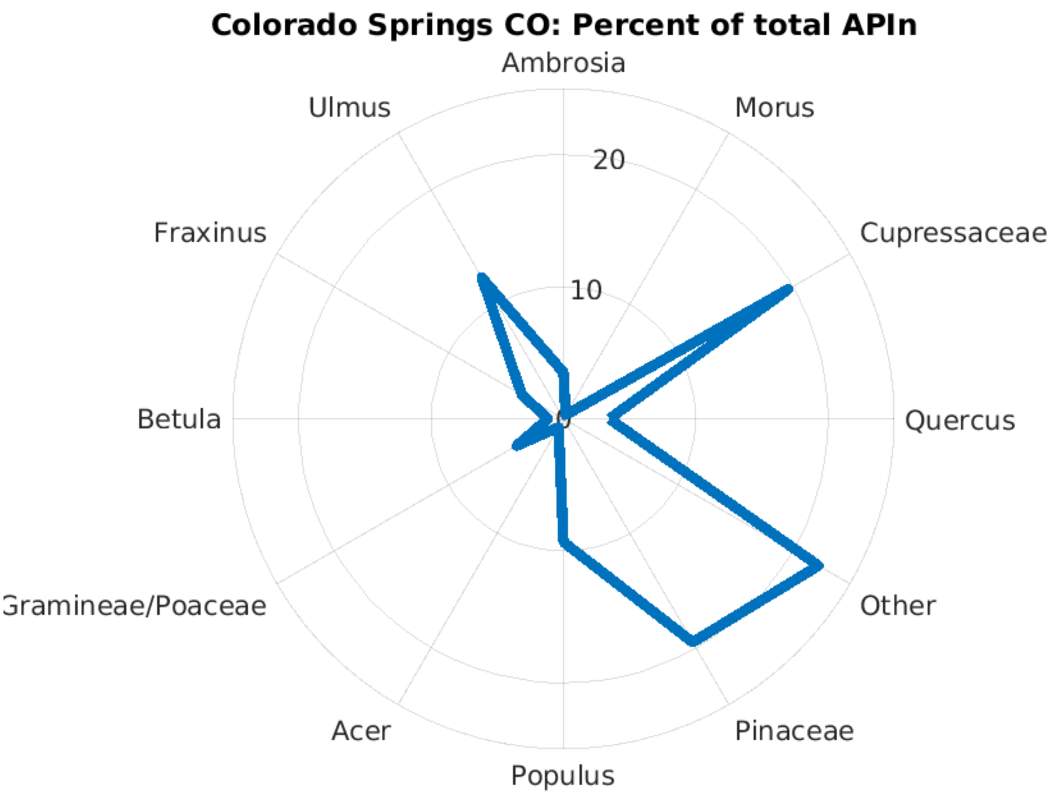
Colorado Springs CO: Percent of total APIn
Ambrosia
Morus
Cupressaceae
Quercus
Other
Pinaceae
Populus
Acer
Gramineae/Poaceae
Betula
Fraxinus
Ulmus
20
10
0

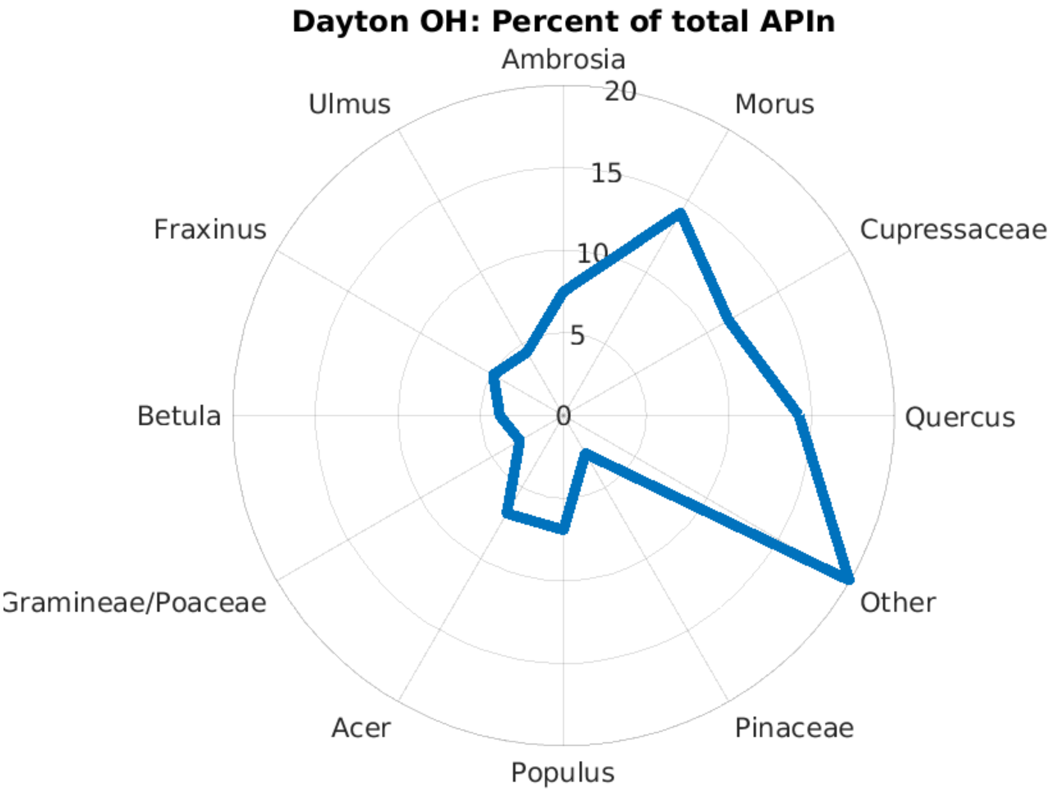
Dayton OH: Percent of total APIn
Ambrosia
Morus
Cupressaceae
Quercus
Other
Pinaceae
Populus
Acer
Gramineae/Poaceae
Betula
Fraxinus
Ulmus
20
15
10
5
0

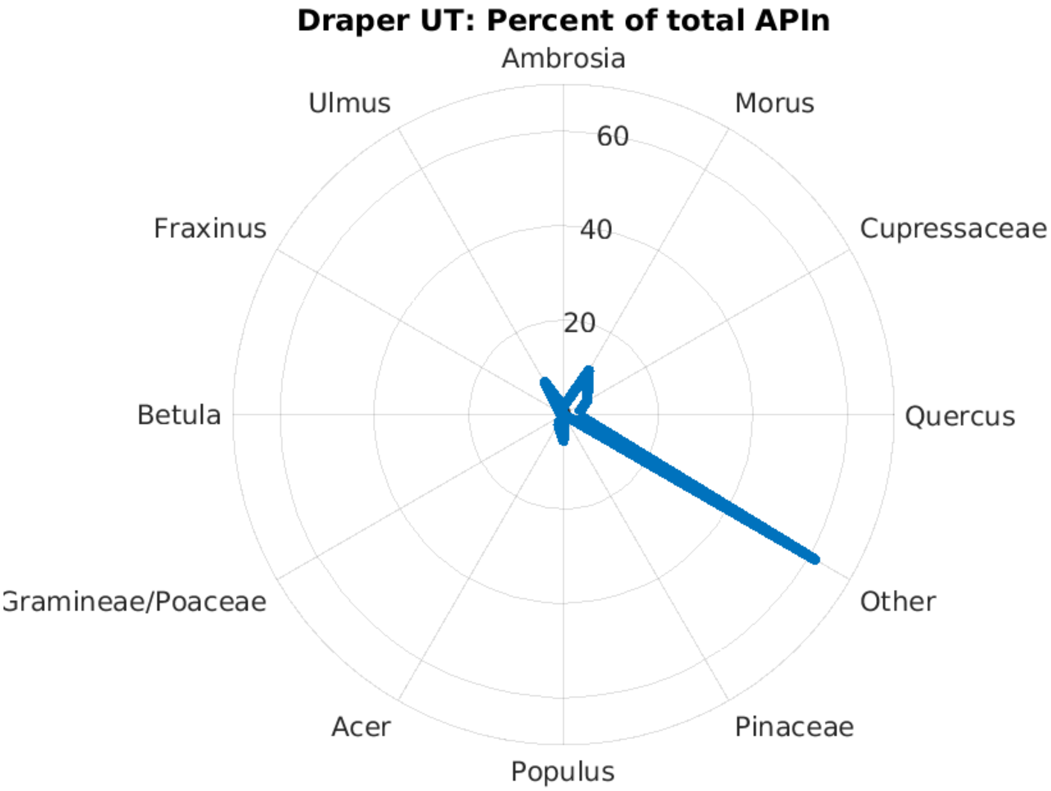
Draper UT: Percent of total APIn
Ambrosia
Morus
Cupressaceae
Quercus
Other
Pinaceae
Populus
Acer
Gramineae/Poaceae
Betula
Fraxinus
Ulmus
20
40
60

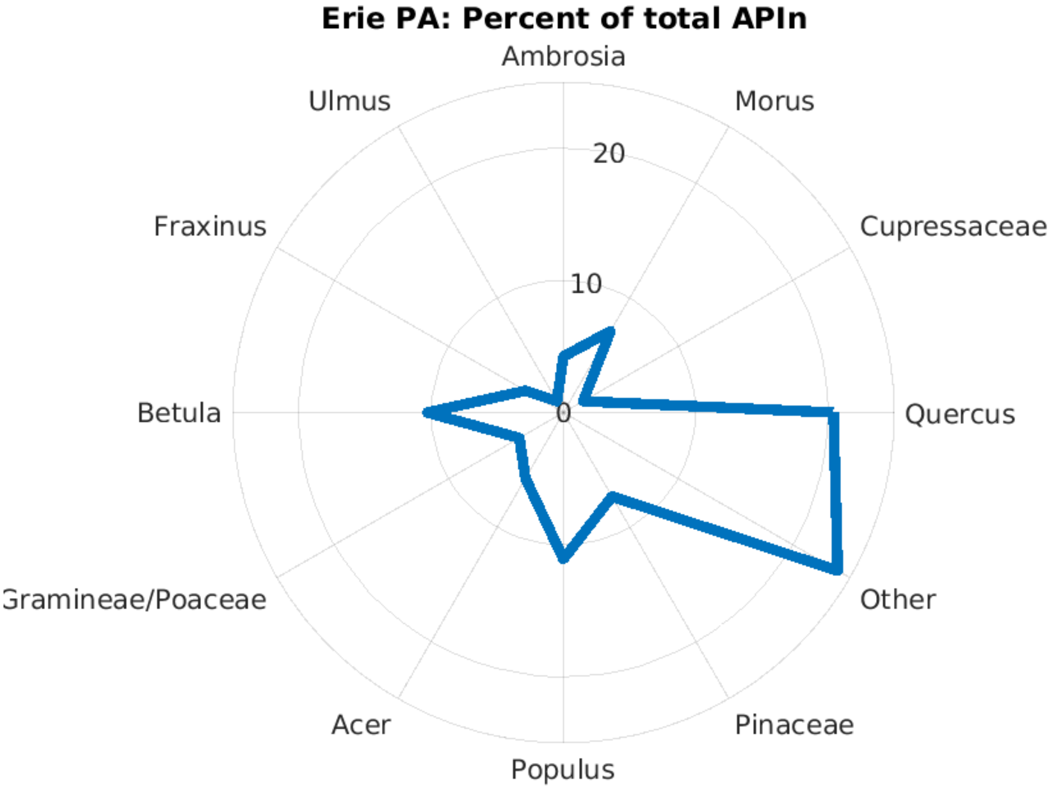
Erie PA: Percent of total APIn
Ambrosia
Morus
Cupressaceae
Quercus
Other
Pinaceae
Populus
Acer
Gramineae/Poaceae
Betula
Fraxinus
Ulmus
0
10
20

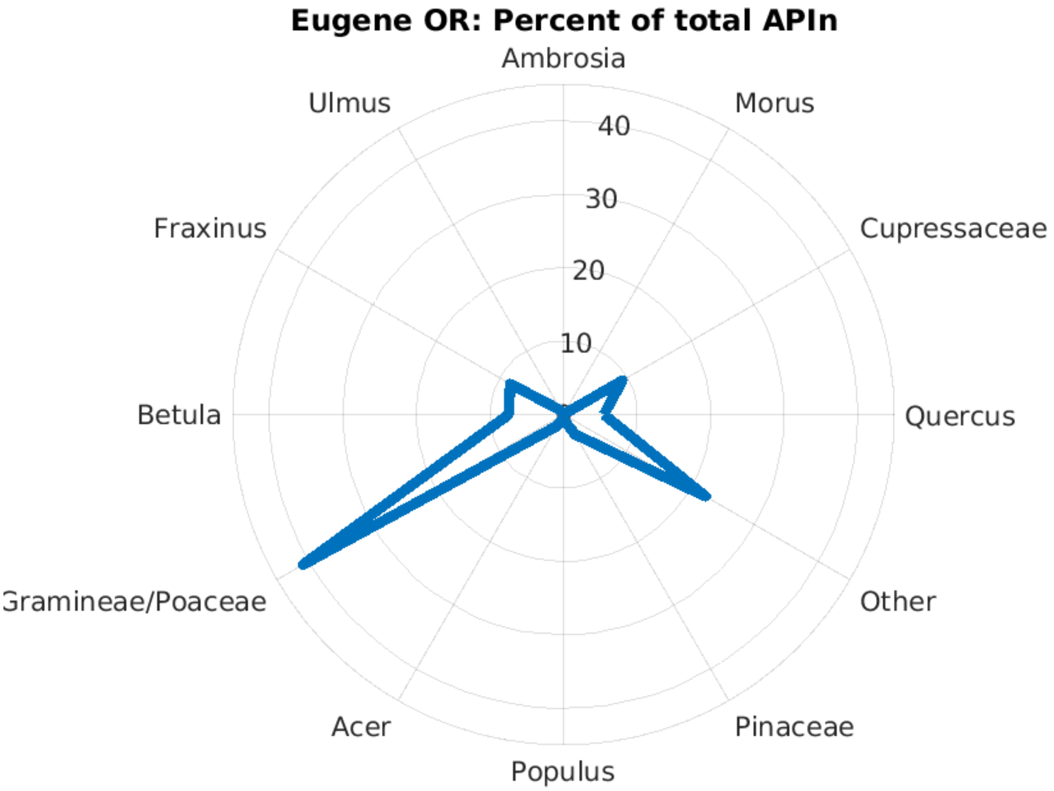

Eugene OR: Percent of total APIn
Ambrosia
Morus
Cupressaceae
Quercus
Other
Pinaceae
Populus
Acer
Gramineae/Poaceae
Betula
Fraxinus
Ulmus
10
20
30
40

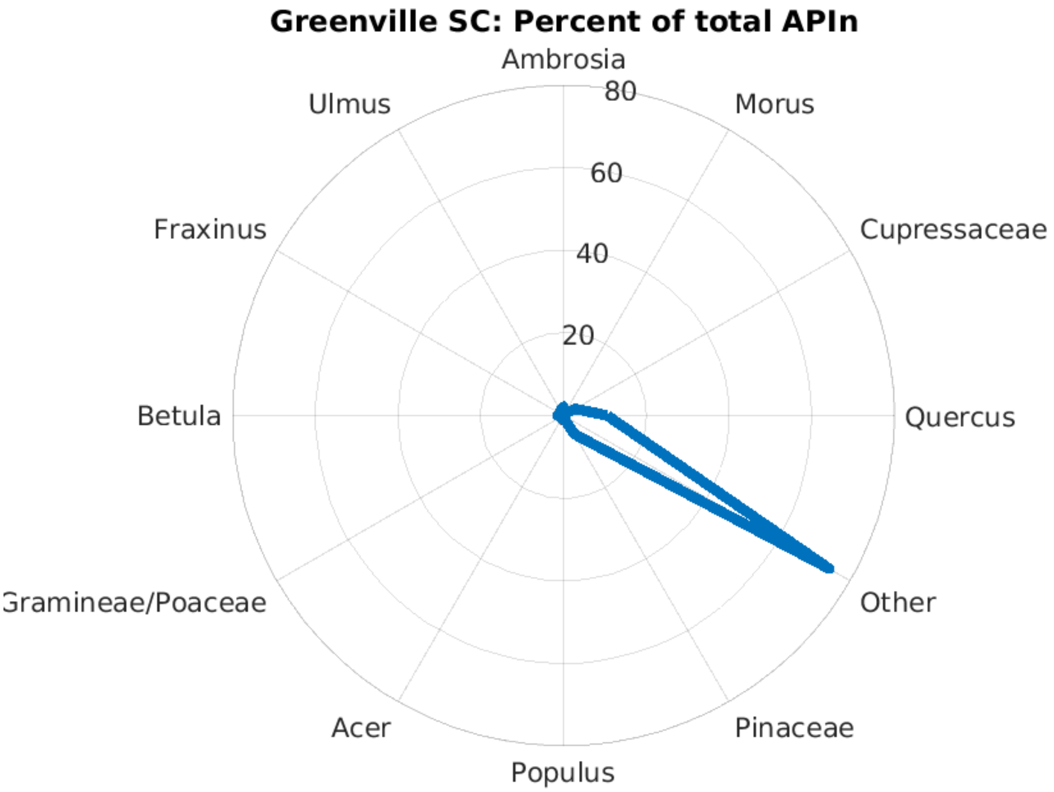

Greenville SC: Percent of total APIn
Ambrosia
Morus
Cupressaceae
Quercus
Other
Pinaceae
Populus
Acer
Gramineae/Poaceae
Betula
Fraxinus
Ulmus
20
40
60
80

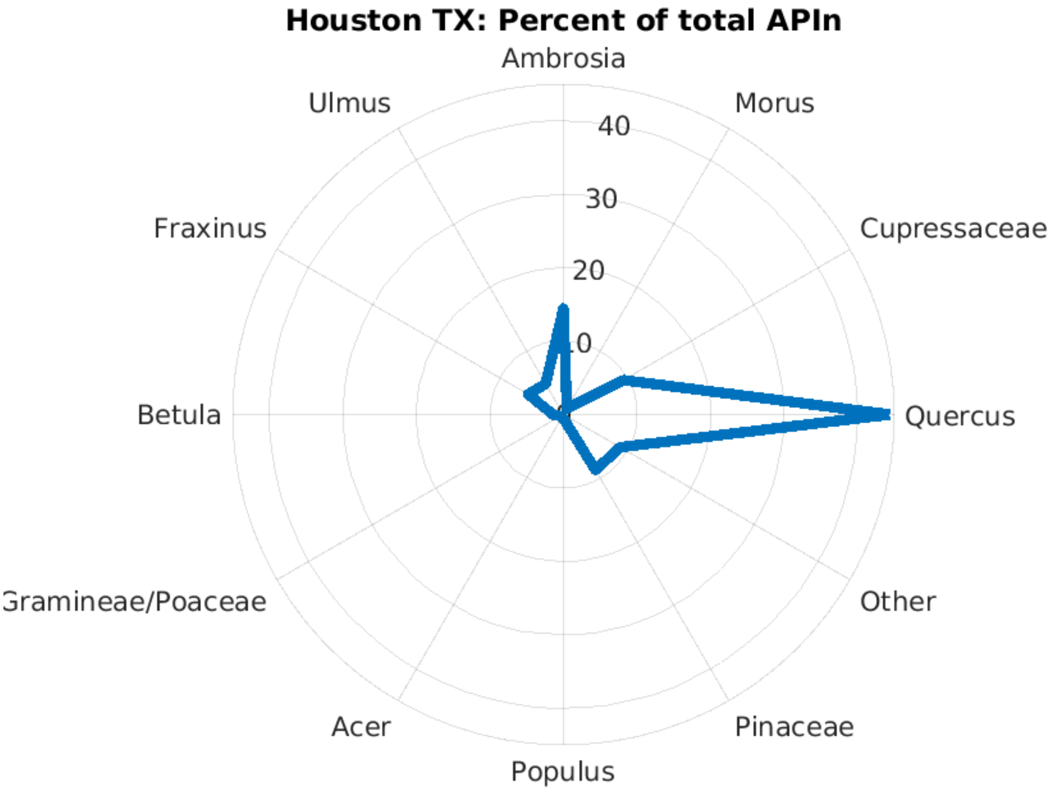
Houston TX: Percent of total APIn
Ambrosia
Morus
Cupressaceae
Quercus
Other
Pinaceae
Populus
Acer
ramineae/Poaceae
Betula
Fraxinus
Ulmus
40
30
20

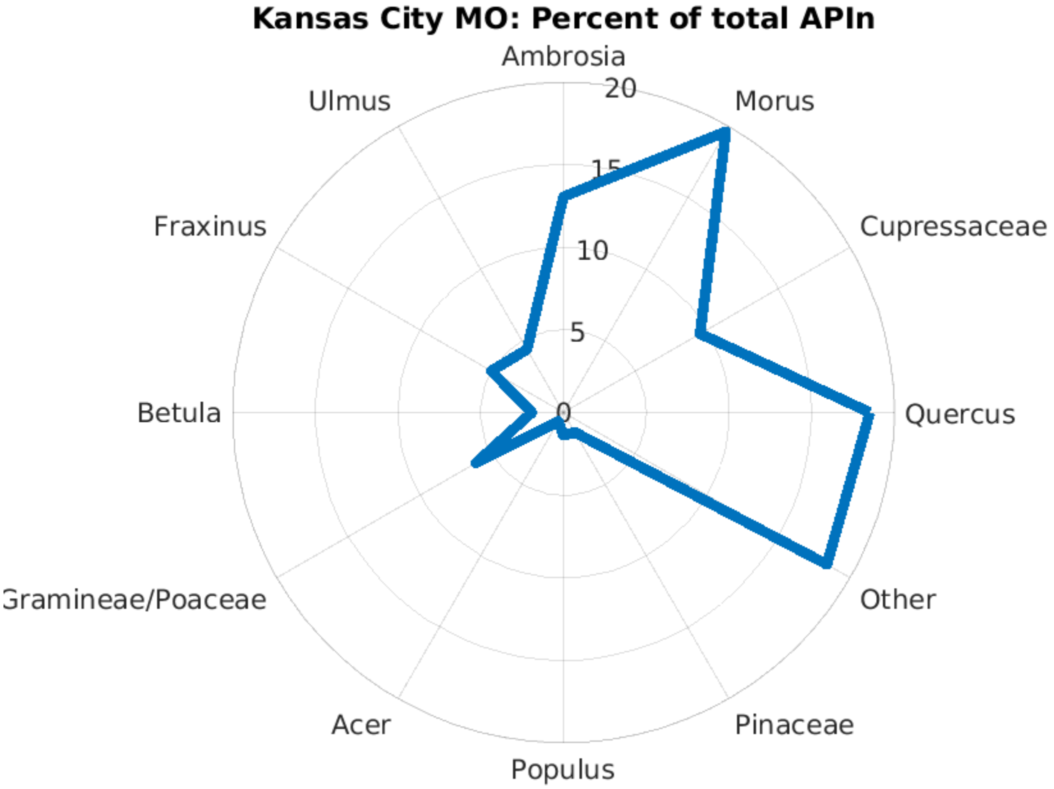
Kansas City MO: Percent of total APIn
Ambrosia
Morus
Cupressaceae
Quercus
Other
Pinaceae
Populus
Acer
ramineae/Poaceae
Betula
Fraxinus
Ulmus
20
10
5
0

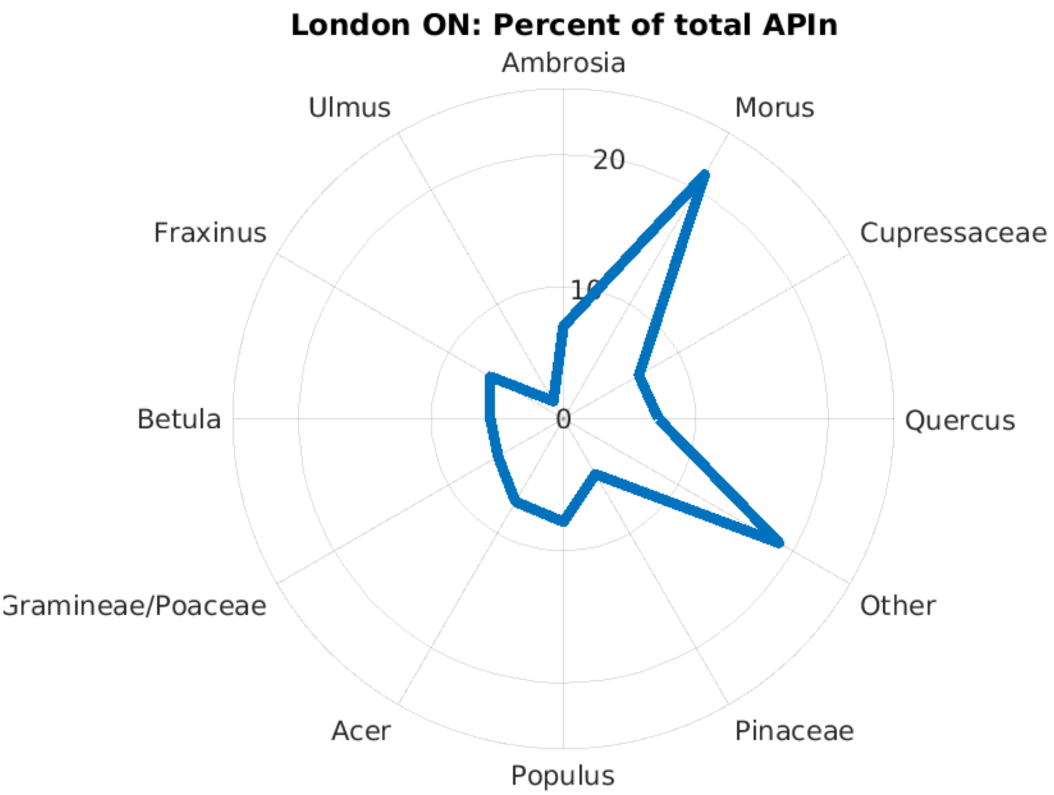
London ON: Percent of total APIn
Ambrosia
Morus
Cupressaceae
Quercus
Other
Pinaceae
Populus
Acer
Gramineae/Poaceae
Betula
Fraxinus
Ulmus
0
10
20

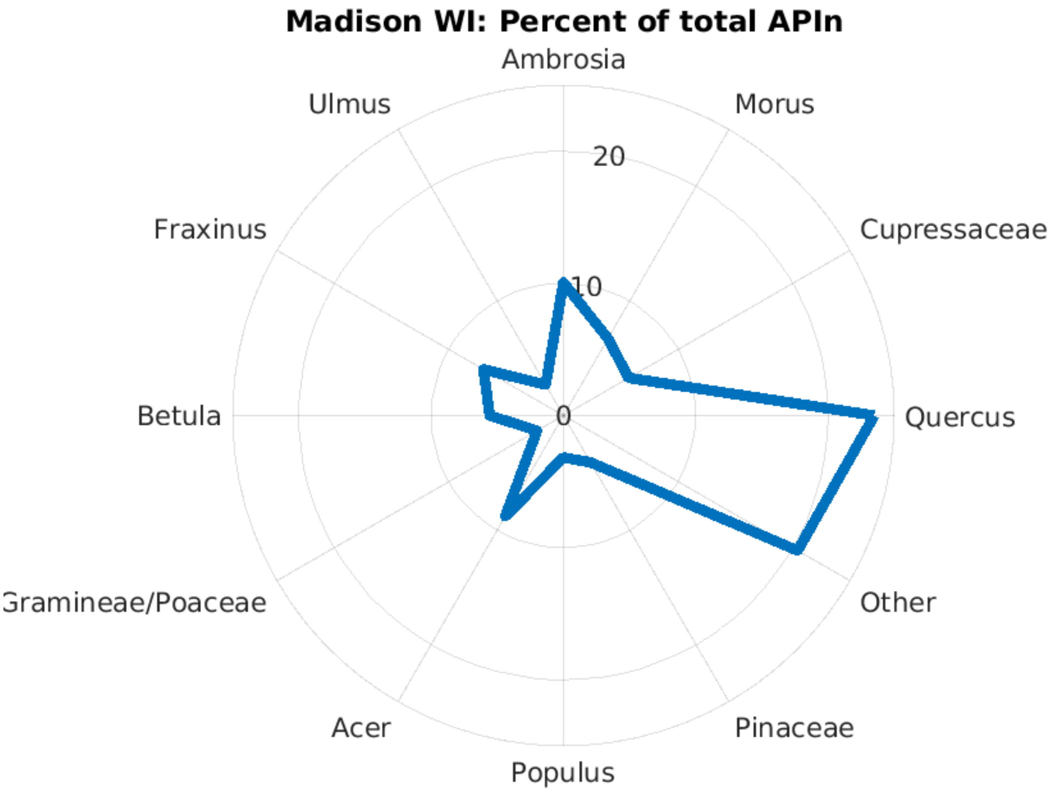
Madison WI: Percent of total APIn
Ambrosia
Morus
Cupressaceae
Quercus
Other
Pinaceae
Populus
Acer
Gramineae/Poaceae
Betula
Fraxinus
Ulmus
0
10
20

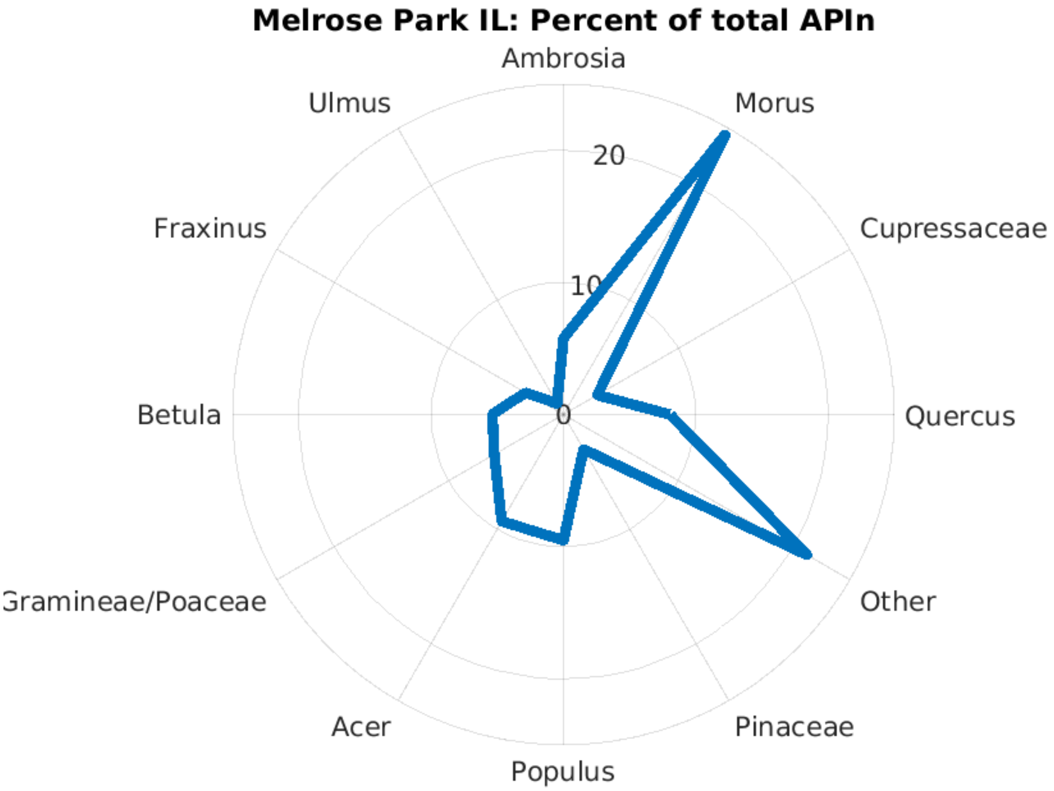
Melrose Park IL: Percent of total APIn
Ambrosia
Morus
Cupressaceae
Quercus
Other
Pinaceae
Populus
Acer
Gramineae/Poaceae
Betula
Fraxinus
Ulmus
0
10
20

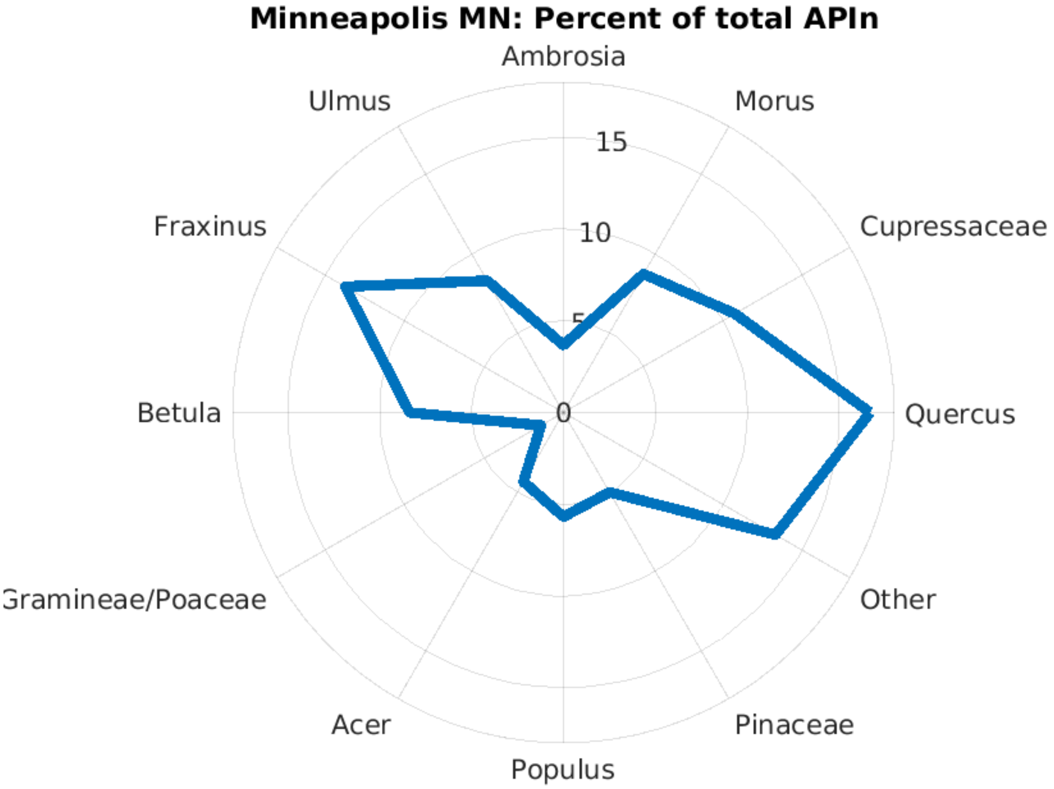
Minneapolis MN: Percent of total APIn
Ambrosia
Morus
Cupressaceae
Quercus
Other
Pinaceae
Populus
Acer
Gramineae/Poaceae
Betula
Fraxinus
Ulmus
0
10
15

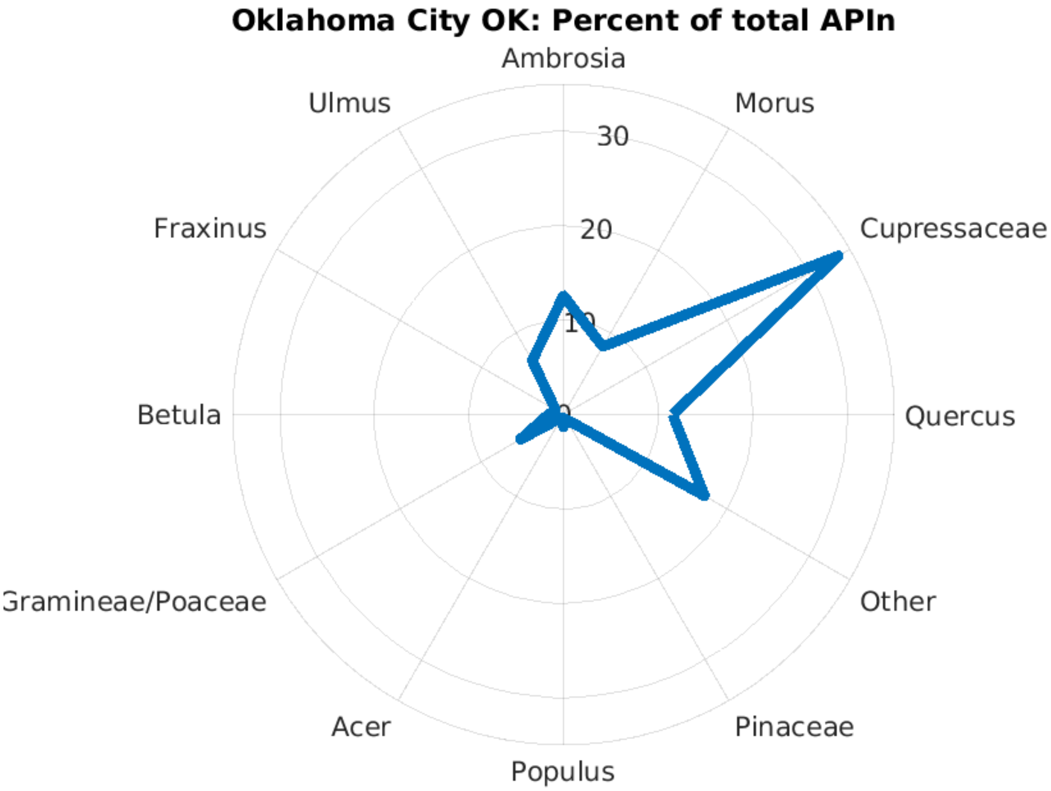
Oklahoma City OK: Percent of total APIn
Ambrosia
Morus
Cupressaceae
Quercus
Other
Pinaceae
Populus
Acer
Gramineae/Poaceae
Betula
Fraxinus
Ulmus
30
20
10
0

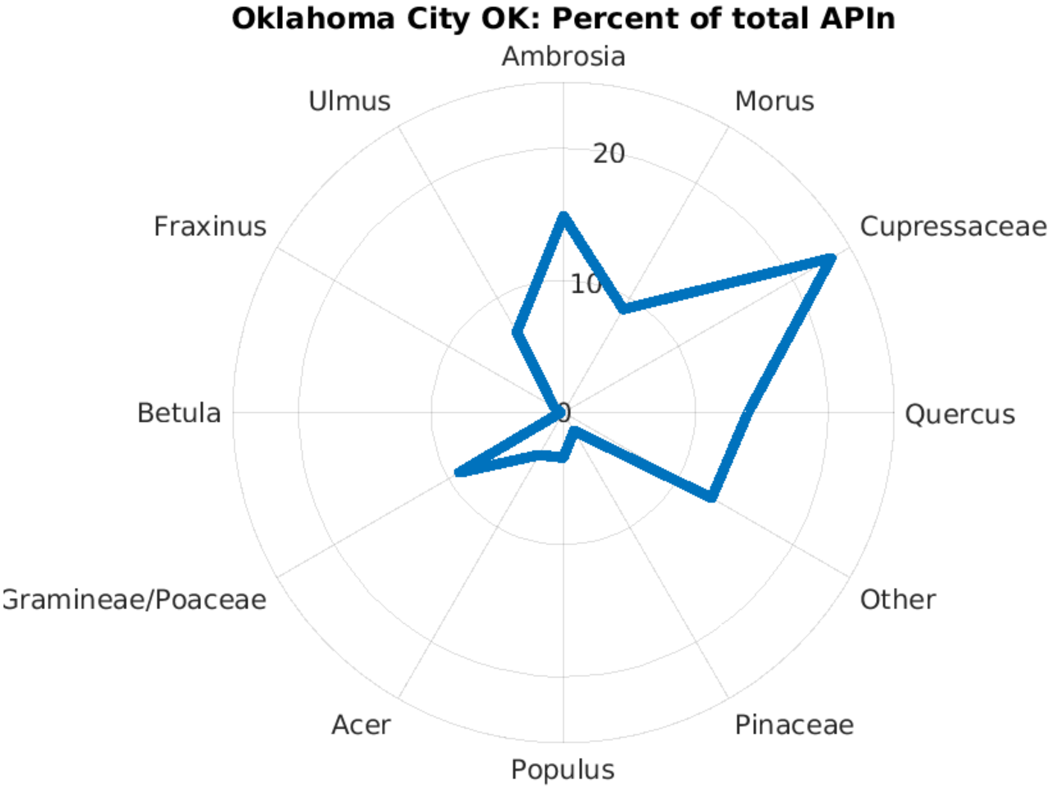
Oklahoma City OK: Percent of total APIn
Ambrosia
Morus
Cupressaceae
Quercus
Other
Pinaceae
Populus
Acer
Gramineae/Poaceae
Betula
Fraxinus
Ulmus
20
10
0

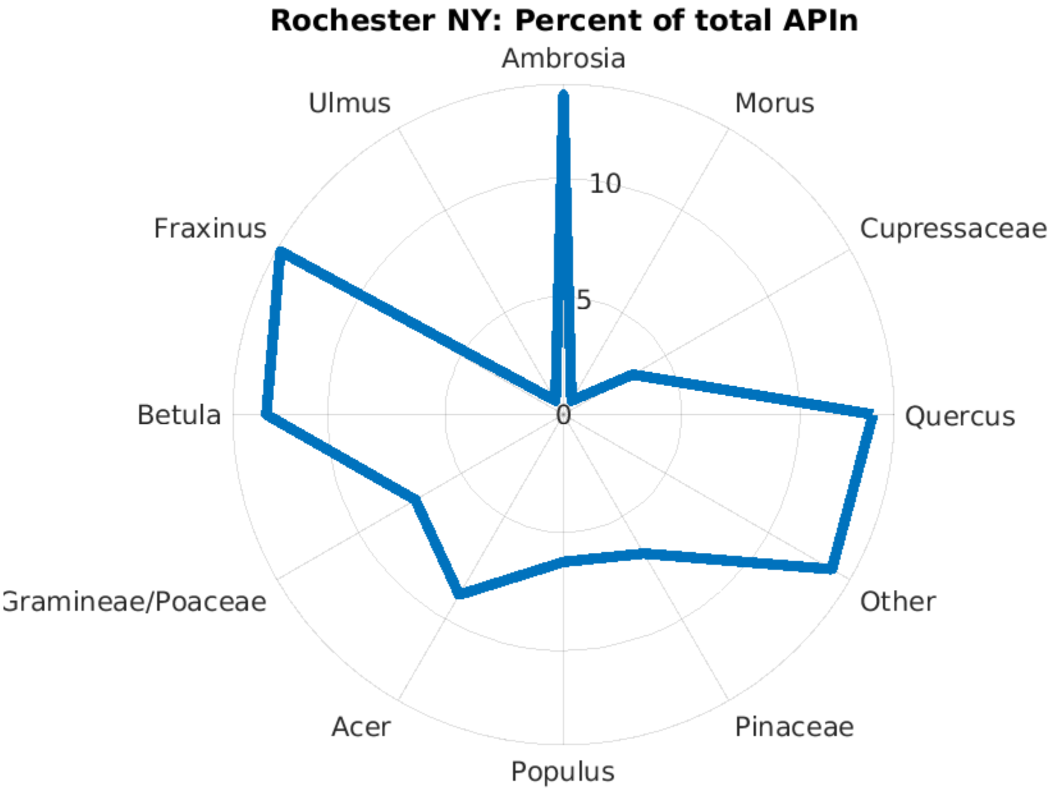
Rochester NY: Percent of total APIn
Ambrosia
Morus
Cupressaceae
Quercus
Other
Pinaceae
Populus
Acer
Gramineae/Poaceae
Betula
Fraxinus
Ulmus
10
5
0

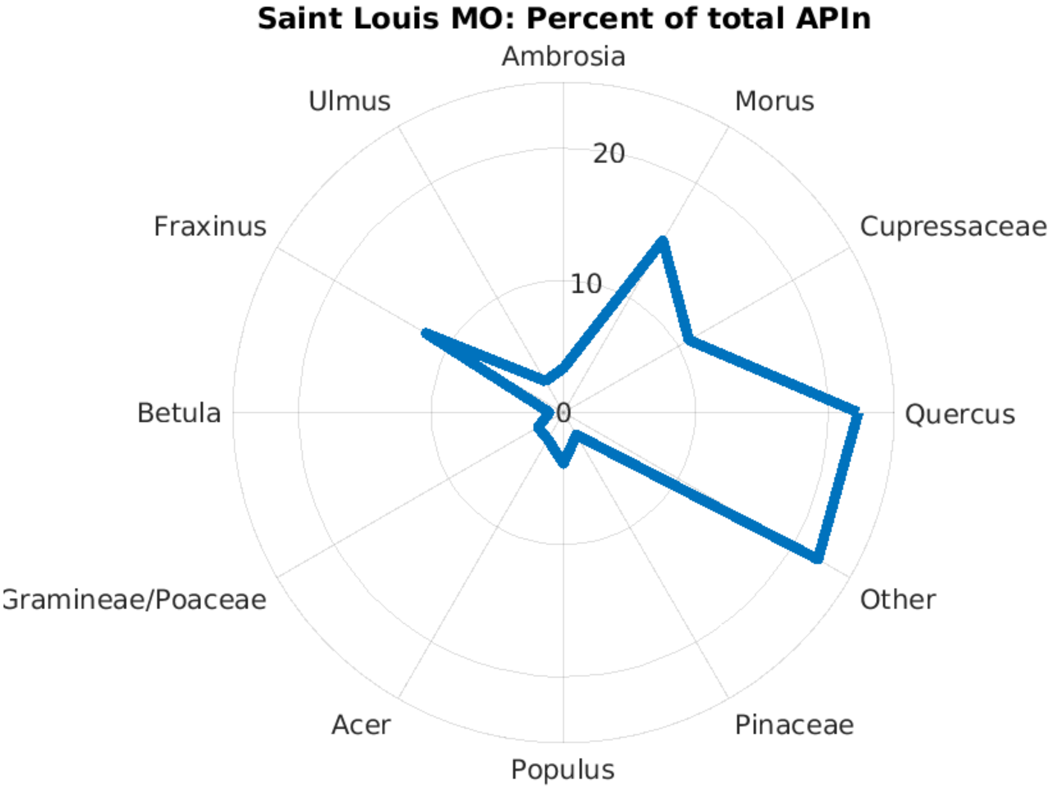
Saint Louis MO: Percent of total APIn
Ambrosia
Morus
Cupressaceae
Quercus
Other
Pinaceae
Populus
Acer
Gramineae/Poaceae
Betula
Fraxinus
Ulmus
20
10
0

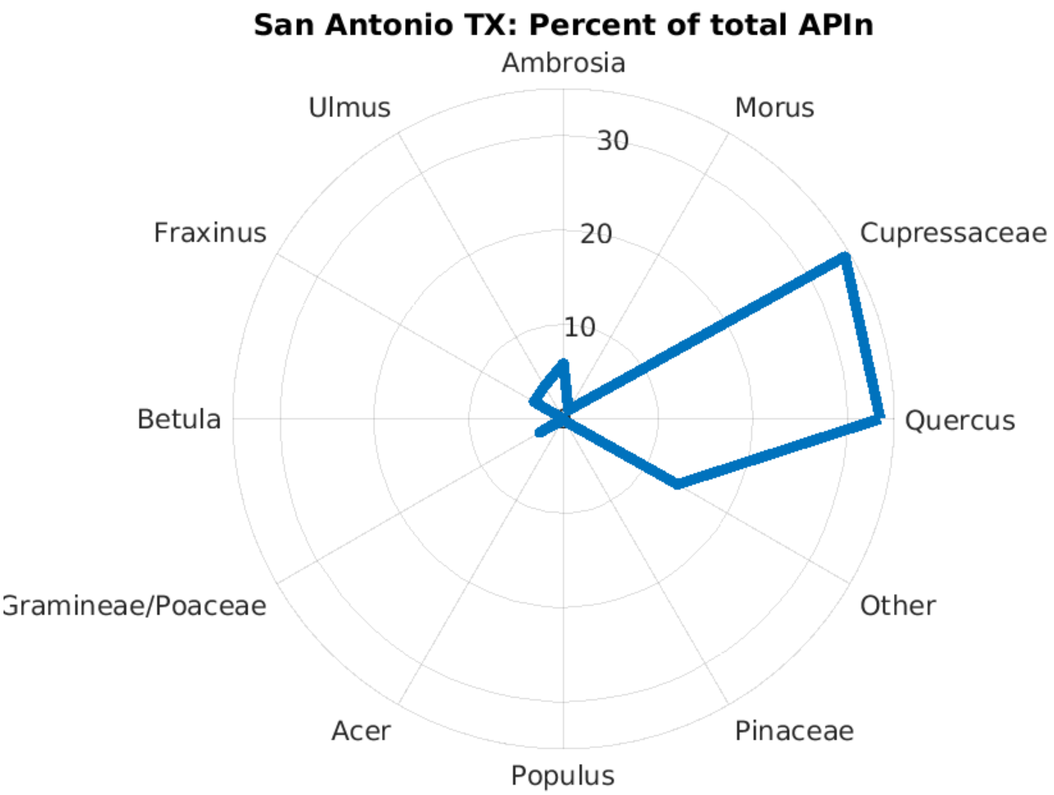
San Antonio TX: Percent of total APIn
Ambrosia
Morus
Cupressaceae
Quercus
Other
Pinaceae
Populus
Acer
Gramineae/Poaceae
Betula
Fraxinus
Ulmus
10
20
30

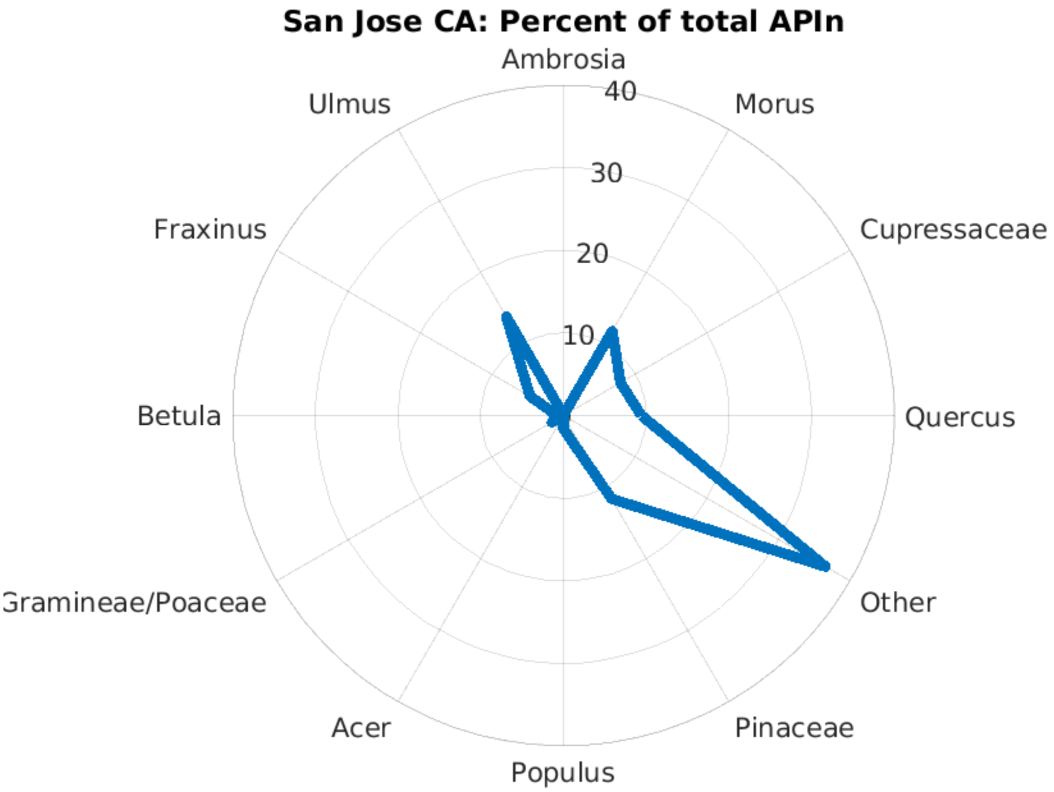
San Jose CA: Percent of total APIn
Ambrosia
Morus
Cupressaceae
Quercus
Other
Pinaceae
Populus
Acer
Gramineae/Poaceae
Betula
Fraxinus
Ulmus
10
20
30
40

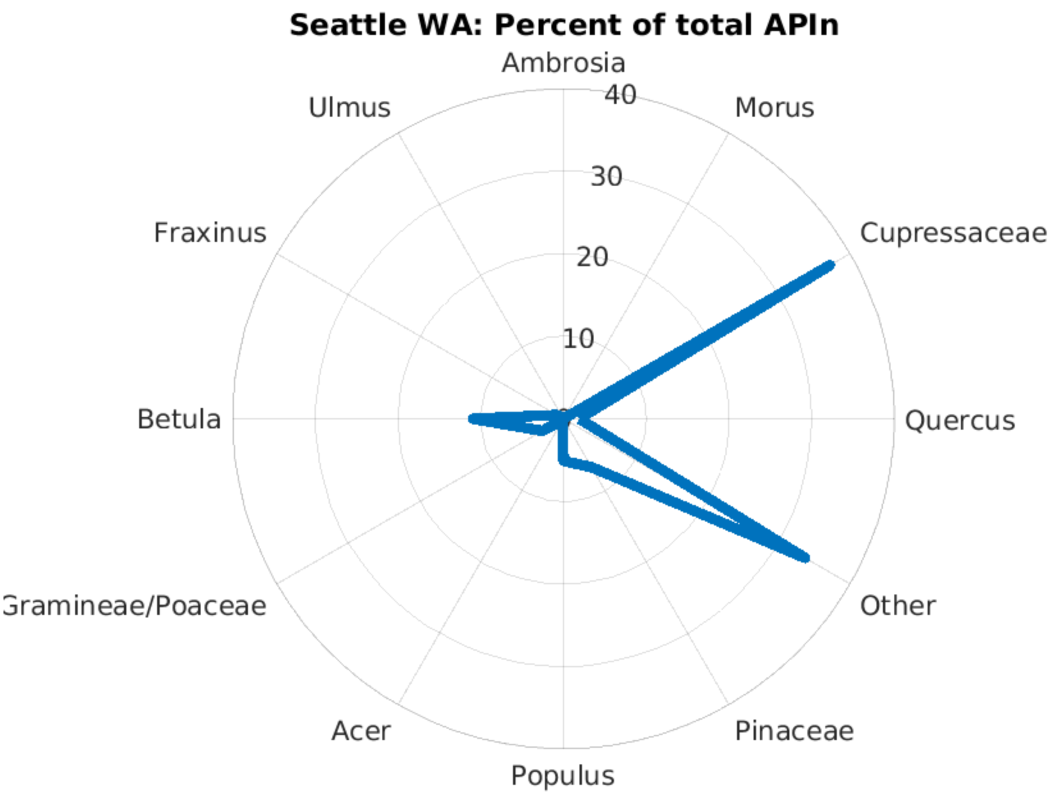
Seattle WA: Percent of total APIn
Ambrosia
Morus
Cupressaceae
Quercus
Other
Pinaceae
Populus
Acer
ramineae/Poaceae
Betula
Fraxinus
Ulmus
40
30
20
10

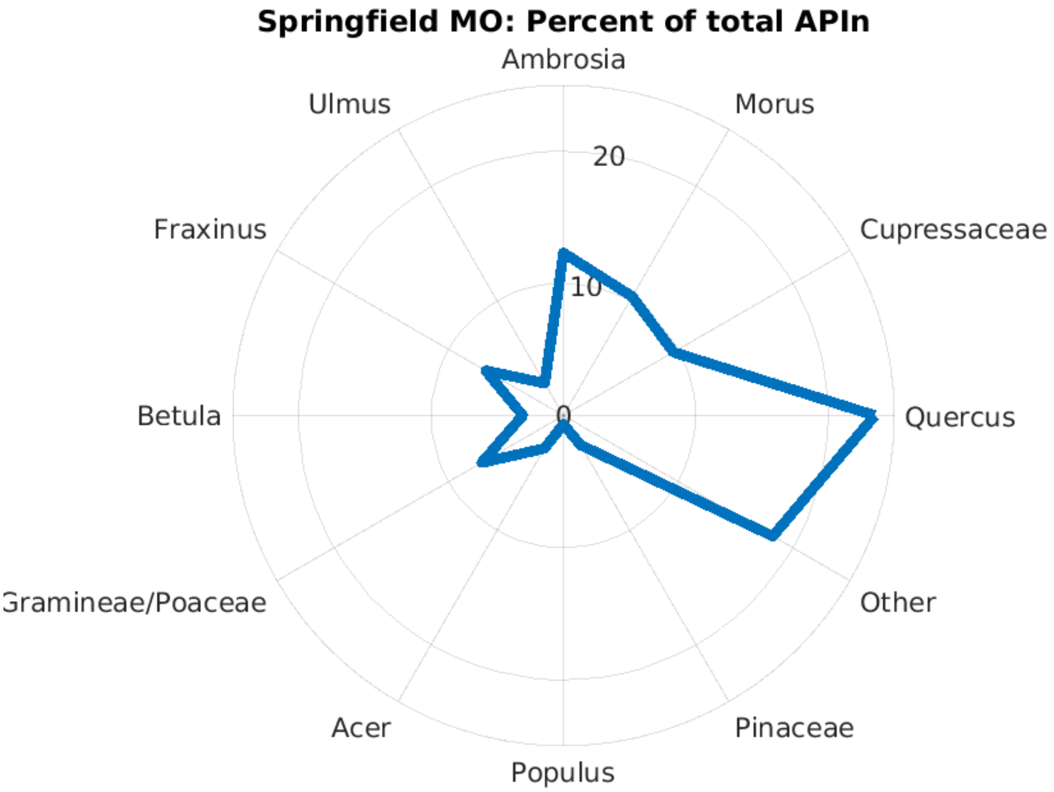
Springfield MO: Percent of total APIn
Ambrosia
Morus
Cupressaceae
Quercus
Other
Pinaceae
Populus
Acer
ramineae/Poaceae
Betula
Fraxinus
Ulmus
20
10
0

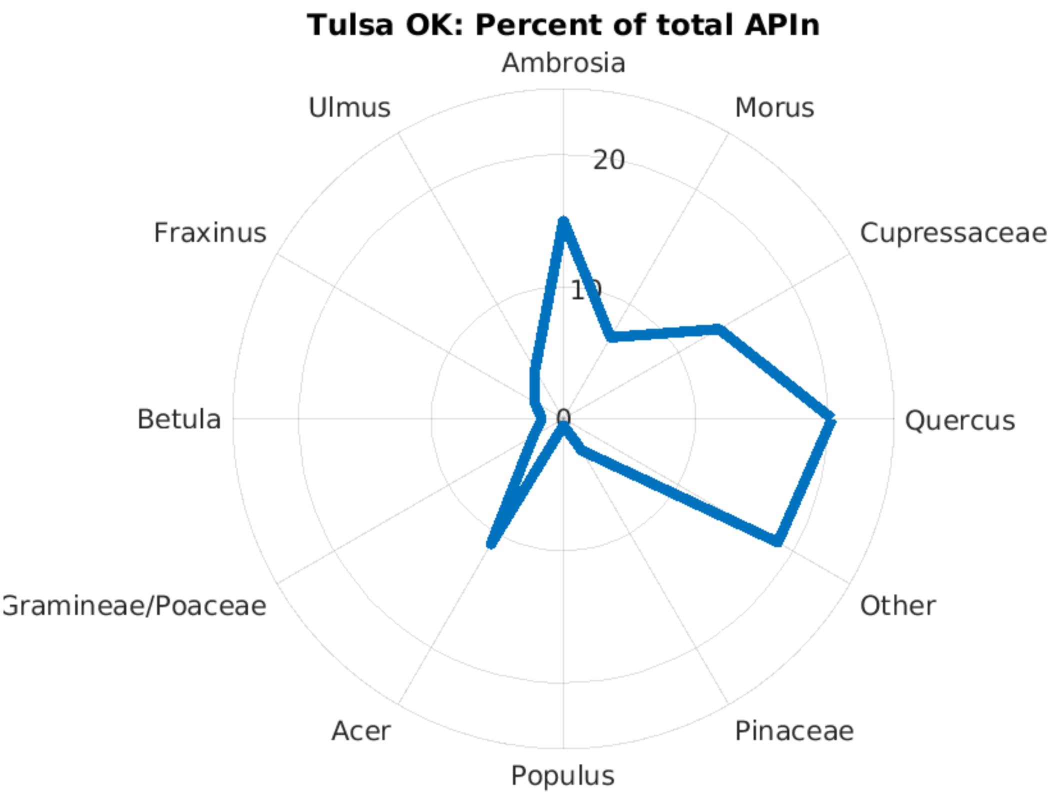
Tulsa OK: Percent of total APIn
Ambrosia
Morus
Cupressaceae
Quercus
Other
Pinaceae
Populus
Acer
Gramineae/Poaceae
Betula
Fraxinus
Ulmus
20
0

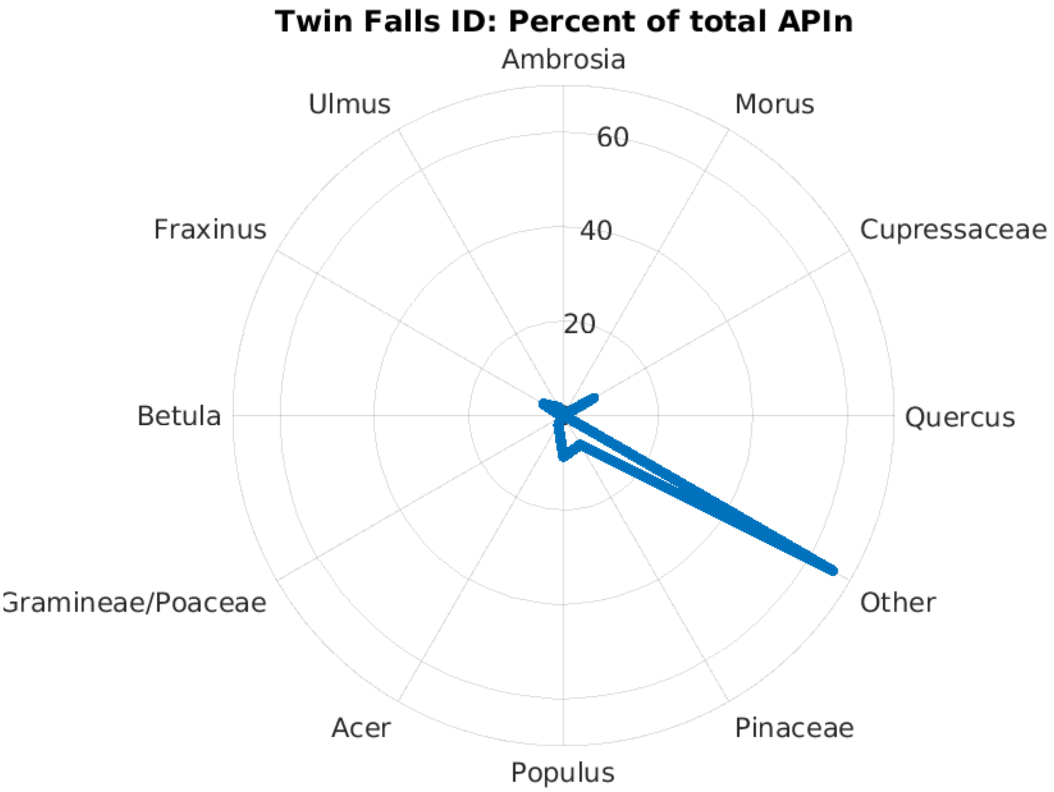
Twin Falls ID: Percent of total APIn
Ambrosia
Morus
Cupressaceae
Quercus
Other
Pinaceae
Populus
Acer
Gramineae/Poaceae
Betula
Fraxinus
Ulmus
60
40
20

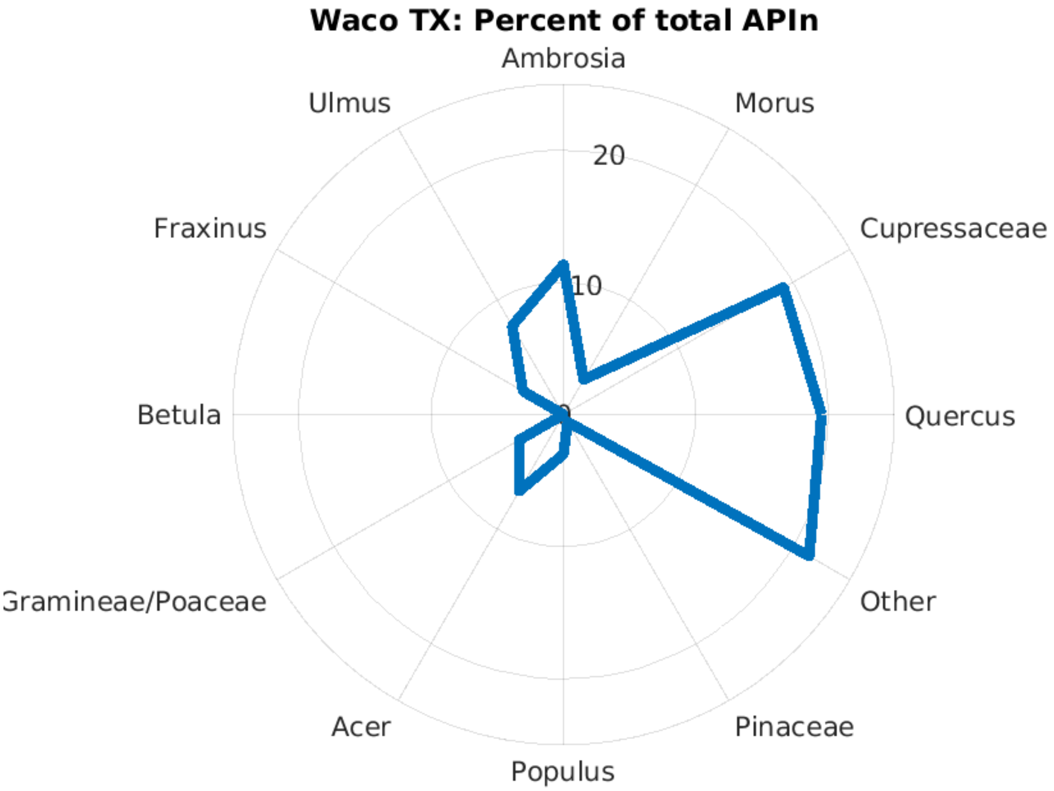
Waco TX: Percent of total APIn
Ambrosia
Morus
Cupressaceae
Quercus
Other
Pinaceae
Populus
Acer
ramineae/Poaceae
Betula
Fraxinus
Ulmus
20
10
0

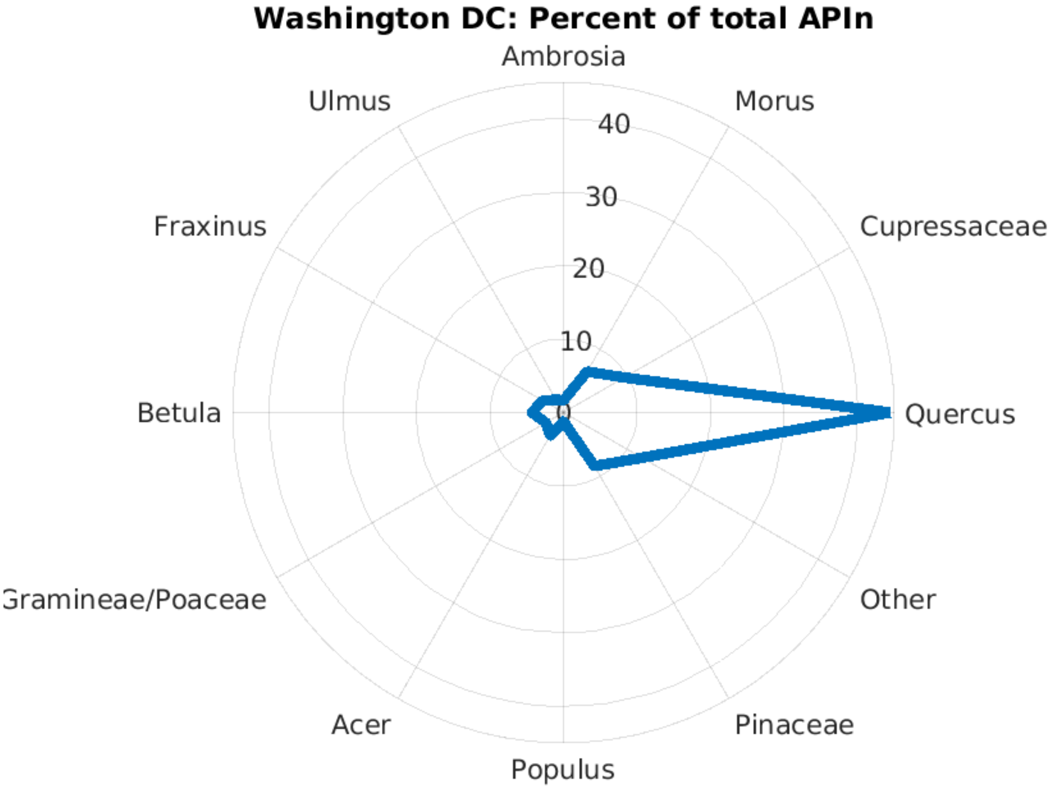
Washington DC: Percent of total APIn
Ambrosia
Morus
Cupressaceae
Quercus
Other
Pinaceae
Populus
Acer
ramineae/Poaceae
Betula
Fraxinus
Ulmus
40
30
20
10
0

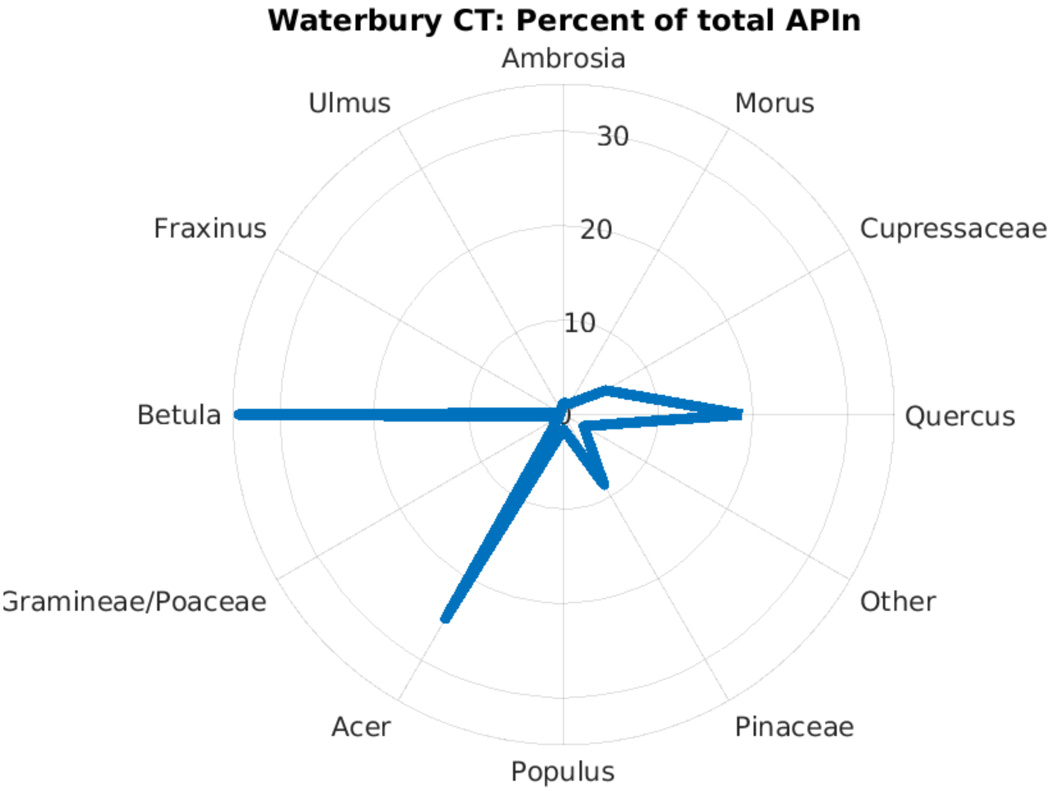
Waterbury CT: Percent of total APIn
Ambrosia
Morus
Cupressaceae
Quercus
Other
Pinaceae
Populus
Acer
Gramineae/Poaceae
Betula
Fraxinus
Ulmus
30
20
10
0

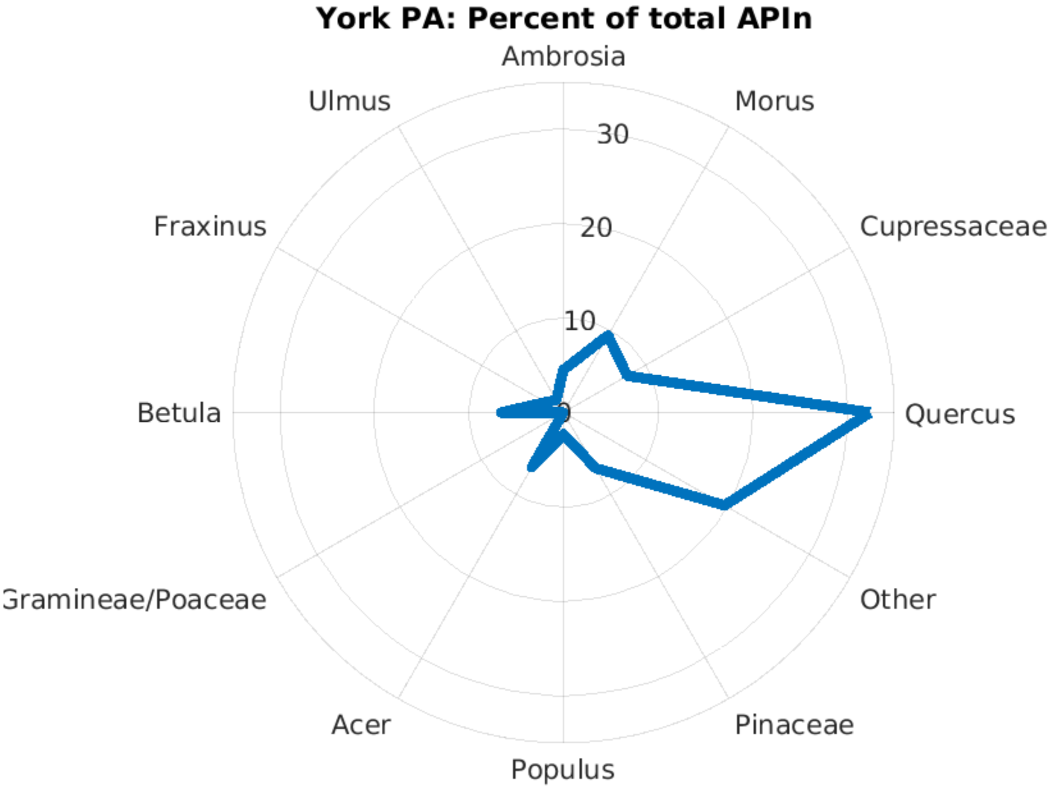
York PA: Percent of total APIn
Ambrosia
Morus
Cupressaceae
Quercus
Other
Pinaceae
Populus
Acer
Gramineae/Poaceae
Betula
Fraxinus
Ulmus
30
20
10
0
